# Mobile Application–Based Communication Facilitation Platform for Family Members of Critically Ill Patients: A Randomized Clinical Trial

**DOI:** 10.1001/jamanetworkopen.2023.49666

**Published:** 2024-01-04

**Authors:** Christopher E. Cox, Deepshikha C. Ashana, Isaretta L. Riley, Maren K. Olsen, David Casarett, Krista L. Haines, Yasmin Ali O’Keefe, Mashael Al-Hegelan, Robert W. Harrison, Colleen Naglee, Jason N. Katz, Hongqiu Yang, Elias H. Pratt, Jessie Gu, Katelyn Dempsey, Sharron L. Docherty, Kimberly S. Johnson

**Affiliations:** 1 Department of Medicine, Division of Pulmonary and Critical Care Medicine, Duke University, Durham, North Carolina; 2 Program to Support People and Enhance Recovery (ProSPER), Duke University, Durham, North Carolina; 3 Department of Biostatistics and Bioinformatics, Duke University, Durham, North Carolina; 4 Durham Center of Innovation to Accelerate Discovery and Practice Transformation, Durham Veterans Affairs Health Care System, Durham, North Carolina; 5 Department of Medicine, Section of Palliative Care and Hospice Medicine, Duke University, Durham, North Carolina; 6 Department of Surgery, Division of Trauma and Critical Care and Acute Care Surgery, Duke University, Durham, North Carolina; 7 Department of Anesthesiology, Duke University, Durham, North Carolina; 8 Department of Medicine, Division of Cardiology, Duke University, Durham, North Carolina; 9 Duke Clinical Research Institute, Duke University, Durham, North Carolina; 10 School of Nursing, Duke University, Durham, North Carolina; 11 Department of Medicine, Division of Geriatrics, Duke University, Durham, North Carolina; 12 Geriatrics Research, Education, and Clinical Center, Veterans Affairs Health Care System, Durham, North Carolina

## Abstract

**Importance:**

Unmet and racially disparate palliative care needs are common in intensive care unit (ICU) settings.

**Objective:**

To test the effect of a primary palliative care intervention vs usual care control both overall and by family member race.

**Design, Setting, and Participants:**

This cluster randomized clinical trial was conducted at 6 adult medical and surgical ICUs in 2 academic and community hospitals in North Carolina between April 2019 and May 2022 with physician-level randomization and sequential clusters of 2 Black patient–family member dyads and 2 White patient–family member dyads enrolled under each physician. Eligible participants included consecutive patients receiving mechanical ventilation, their family members, and their attending ICU physicians. Data analysis was conducted from June 2022 to May 2023.

**Intervention:**

A mobile application (ICUconnect) that displayed family-reported needs over time and provided ICU attending physicians with automated timeline-driven communication advice on how to address individual needs.

**Main Outcomes and Measures:**

The primary outcome was change in the family-reported Needs at the End-of-Life Screening Tool (NEST; range 0-130, with higher scores reflecting greater need) score between study days 1 and 3. Secondary outcomes included family-reported quality of communication and symptoms of depression, anxiety, and posttraumatic stress disorder at 3 months.

**Results:**

A total of 111 (51% of those approached) family members (mean [SD] age, 51 [15] years; 96 women [86%]; 15 men [14%]; 47 Black family members [42%]; 64 White family members [58%]) and 111 patients (mean [SD] age, 55 [16] years; 66 male patients [59%]; 45 Black patients [41%]; 65 White patients [59%]; 1 American Indian or Alaska Native patient [1%]) were enrolled under 37 physicians randomized to intervention (19 physicians and 55 patient–family member dyads) or control (18 physicians and 56 patient–family member dyads). Compared with control, there was greater improvement in NEST scores among intervention recipients between baseline and both day 3 (estimated mean difference, −6.6 points; 95% CI, −11.9 to −1.3 points; *P* = .01) and day 7 (estimated mean difference, −5.4 points; 95% CI, −10.7 to 0.0 points; *P* = .05). There were no treatment group differences at 3 months in psychological distress symptoms. White family members experienced a greater reduction in NEST scores compared with Black family members at day 3 (estimated mean difference, −12.5 points; 95% CI, −18.9 to −6.1 points; *P* < .001 vs estimated mean difference, −0.3 points; 95% CI, −9.3 to 8.8 points; P = .96) and day 7 (estimated mean difference, −9.5 points; 95% CI, −16.1 to −3.0 points; *P* = .005 vs estimated mean difference, −1.4 points; 95% CI, −10.7 to 7.8; *P* = .76).

**Conclusions and Relevance:**

In this study of ICU patients and family members, a primary palliative care intervention using a mobile application reduced unmet palliative care needs compared with usual care without an effect on psychological distress symptoms at 3 months; there was a greater intervention effect among White family members compared with Black family members. These findings suggest that a mobile application–based intervention is a promising primary palliative care intervention for ICU clinicians that directly addresses the limited supply of palliative care specialists.

**Trial Registration:**

ClinicalTrials.gov Identifier: NCT03506438

## Key Points

**Question:**

Can a mobile application–based primary palliative care intervention targeting intensive care unit (ICU) physicians and family members improve unmet palliative care needs overall, and are there different outcomes by race?

**Findings:**

In this cluster randomized clinical trial of 111 patient–family member dyads, users of the application intervention experienced significant improvements in need severity compared with the usual care control group. There was a significant intervention effect among White family members but not Black family members; Black family members in intervention and control groups experienced a similar improvement in needs.

**Meaning:**

These findings suggest that a mobile application is a promising primary palliative care intervention for ICU clinicians that directly addresses the limited supply of palliative care specialists.

## Teaser

This randomized clinical trial analyzes the efficacy of a mobile application communication platform for addressing the needs of family members of critically ill patients.

## Introduction

Critical illness and its intensive care unit (ICU) management are deeply challenging for those who experience it. Patients commonly experience burdensome symptoms and many experience impersonalized deaths in a procedure-focused setting.^1,2,3^ Their family members often struggle with prognostic and decisional uncertainty as surrogate decision makers in an intimidating environment staffed by large teams of unfamiliar clinicians.^4,5,6^ Palliative care is, therefore, highly relevant in ICU settings because it aims to alleviate symptoms, improve or maintain quality of life, and provide family support.^7^

Although the awareness of palliative care principles has steadily increased in ICU settings, palliative care delivery remains highly variable.^8,9,10^ Palliative care specialists are too few and too geographically concentrated to address the palliative care needs of the nearly 5 million critically ill patients and their family members managed in 5300 US ICUs annually.^11,12^ Experts have proposed addressing this supply-demand mismatch by training the comparably larger ICU clinician workforce to deliver primary palliative care.^13,14^ However, there are few interventions designed to support intensivists in this role.

An additional challenge is the presence of racial disparities in several core elements of ICU-based palliative care.^15,16^ However, a lack of ICU-based palliative care trials that have included racially diverse populations or have examined treatment effects by participant race limit insight about how to address these disparities. Therefore, the aims of this trial were to compare the effect of a novel ICU-based primary palliative care intervention on unmet palliative care needs with usual care, as well as to determine if a differential effect was present among Black family members compared with White family members.

## Methods

### Study Design, Setting, Oversight, and Participants

This parallel-group, randomized clinical trial was approved by the Duke University institutional review board and followed the Consolidated Standards of Reporting Trials (CONSORT) reporting guideline. This trial was conducted with physician-level clustering and 3-month follow-up between April 2019 and May 2022 and included ICU patients, their family members, and their physicians (the trial protocol and statistical analysis plan are shown in Supplement 1).^17^ All participants or their legal representative provided written informed consent.

All attending ICU physicians from 6 adult medical or surgical ICUs in academic hospitals and large community hospitals were eligible for electronic randomization stratified by gender, duration of practice (<10 vs ≥10 years), and primary discipline (medicine vs surgery). To ensure adequate numbers for comparisons by race, we aimed to enroll clusters of 4 family member–patient dyads (2 non-Hispanic Black dyads and 2 non-Hispanic White dyads) under each physician.^18^ Patient inclusion criteria were age (≥18 years) and mechanical ventilation for 48 hours or more. Patients were excluded if death was expected within 24 hours, comfort care was planned, family was unavailable, they were imprisoned, or they received ICU care for more than 1 week. We enrolled 1 family member per patient who self-identified as the individual most involved in the patient’s care. Family members were excluded if they lacked sufficient English knowledge to complete study tasks or if they had low needs at baseline (Needs at the End-of-Life Screening Tool [NEST] scale score <15)^19^ or if the patient regained decision-making capacity, had care assumed by either a nonstudy physician or physician from a different treatment group, or died before the day 3 survey (eAppendix 1 in Supplement 2).

### Intervention

ICUconnect was designed as a pragmatic, automated, timeline-driven, mobile application–based, communication facilitation, digital infrastructure that uses text messages and emails to direct family members and physicians to perform timeline-driven tasks (eg, surveys, content review, and family meetings) across a 7- to 10-day intervention period (eFigure 1 and eFigure 2 in Supplement 2). By providing a standardized process of needs reporting by family members and priming physician behaviors with scripted need-specific prompts, it was intended to address disparities in ICU communication among Black family members who report worse quality of communication than White family members.^20,21,22,23,24,25^ Family member features included a self-report of needs, a list of suggested questions they could use in family meetings to discuss each reported need (eAppendix 2 in Supplement 2), and explanatory videos about basic palliative care concepts. Physician content included data visualizations of all family-reported needs over time (eFigure 3 in Supplement 2), scripted language and tips for addressing each need, an explanatory video, and a text alert if needs increase or remain high (ie, NEST scaled score ≥30) after the first family meeting. The semistructured design of the 2 required family meetings (between study days 1 and 3 to discuss baseline needs and between study days 3 and 7 to discuss needs reported on day 3) was based on expert guidelines.^26,27^ See eAppendix 3 and eAppendix 4 in Supplement 2 for intervention training and family meeting details. Procedures were established for managing changes in ICU attending physicians during the intervention time period for family members (eAppendix 5 in Supplement 2).

### Usual Care

Control family caregivers received usual ICU care without protocolized family meetings. Their reported needs were not visible to physicians.

### Data Collection and Outcomes

Family members and physicians completed surveys electronically. The surveys were sent through secure weblinks automatically texted or emailed from the study data system.

#### Family-Reported Measures

The primary outcome was the NEST scale.^19,28^ We previously adapted the NEST for the ICU setting and conducted a series of evaluations supporting its validity and responsiveness among family member proxies.^29,30,31,32^ The adapted NEST (13 items; total score range: 0 [no need] to 130 [highest need]) was administered on study days 1, 3, and 7 to assess needs across the 8 core domains of palliative care quality, including physical symptoms, structure and processes of care, perceived social support, psychological and emotional symptoms, spiritual and cultural aspects of care, end-of-life care, and ethical aspects of care (see eAppendix 6 in Supplement 2).^33^

Secondary outcomes were assessed at baseline and study days 3 and 7, and included goal concordance of care,^34^ the Quality of Communication Scale^35^ summary item (range: 0-10, with a higher score indicating higher quality communication) and select items on the Interpersonal Processes of Care (IPC) scales,^36^ including Patient-Centeredness of Decision Making, Eliciting Concerns, and Discrimination (scale score range: 0-5, with higher scores indicating higher frequency of the interpersonal process). At baseline and 3 months, family members reported symptoms of depression with the Patient Health Questionnaire-9 scale^37^ (PHQ-9; score range: 0-27, with a higher score being more indicative of depression), anxiety with the Generalized Anxiety Disorder 7 scale^38^ (GAD-7; score range: 0-21, with cutoff scores for mild [5], moderate [10], and severe [15] levels of anxiety symptoms). and posttraumatic stress disorder (PTSD) with the Posttraumatic Stress Syndrome Inventory^39^ (PTSS; score range: 10-70, with a higher score being more indicative of PTSD).

#### Clinician-Reported Variables

ICU clinicians reported their sociodemographics and palliative care attitudes. Sociodemographic information included gender and race and ethnicity. Race categories included American Indian or Alaska Native, Asian, Black or African American, Native Hawaiian or Other Pacific Islander, White, or other (any other race not otherwise specified), and ethnicity categories included Hispanic or Latino and non-Hispanic or Latino. Those randomized to intervention also reported if they conducted study family meetings.

#### Clinical Variables

Family members self-reported their race, ethnicity, and gender. Race categories included American Indian or Alaska Native, Asian, Black or African American, Native Hawaiian or Other Pacific Islander, White, other (any other race not otherwise specified), and not reported or unknown; ethnicity categories included Hispanic or Latino and non-Hispanic or Latino. Study staff recorded patient level data from the electronic medical record. Patient illness severity was measured with the Acute Physiology and Chronic Health Evaluation II,^40^ and patient comorbidities were measured with the Charlson Comorbidity Index.^41^

### Statistical Analysis

The primary hypothesis was that the intervention group would experience a greater reduction in NEST scores between study days 1 and 3 compared with usual care. Additional aims were to assess the intervention effect on Black and White family members separately, as well as the differential intervention effect on Black families compared with White families.

Sample size was based on the mean difference score using tests for 2 means in a cluster randomized design assuming a type I error of 5% and 80% power (PASS statistical software version 20.0.3 [NCSS]). According to preliminary studies, the SD of the change in NEST score was estimated as 12 points with a range of intraclass correlation coefficients (0.01-0.1). To detect differences of 5.4 points to 6.1 points overall and 7.7 points to 8.0 points for Black or White patients separately, we needed to randomize 40 physicians (20 per group) with a cluster size of 4 (2 Black patients and 2 White patients) for a total sample size of 160 families. Although the NEST minimal clinically important difference is unclear, such a targeted difference is significantly associated with clinically important changes in depression and anxiety symptoms, goal concordance, and quality of communication as well as an item threshold value in our recent latent class analyses of serious need.^29,30,31,32^

Hierarchical linear models were used to estimate the treatment effect on changes in NEST scores, as well as to account for the association of both family members’ repeated measurements over time and between family members with the same clinician (ie, clustering). Models were fit in SAS PROC MIXED software version 9.4 (SAS Institute), assuming a compound symmetry covariance structure for the repeated measures and a random variance component for the clustering (eAppendix 7 in Supplement 2). Similar models were fit similarly for the other continuous outcomes. Goal concordant care, IPC Patient-Centeredness of Decision Making (<5 vs 5), and IPC Discrimination (1 vs ≥1) were analyzed as longitudinal binary variables with intervention effects estimated in SAS PROC GENMOD software version 9.4 with generalized linear models with a logit link and robust variance to account for repeated measures correlation and clustering. All models included indicator variables for study day, the intervention by time indicators, and the randomization stratification variables. A 2-sided *P* < .05 was considered statistically significant. Data analysis occurred from June 2022 to May 2023.

## Results

### Participant Characteristics

**Figure 1.**
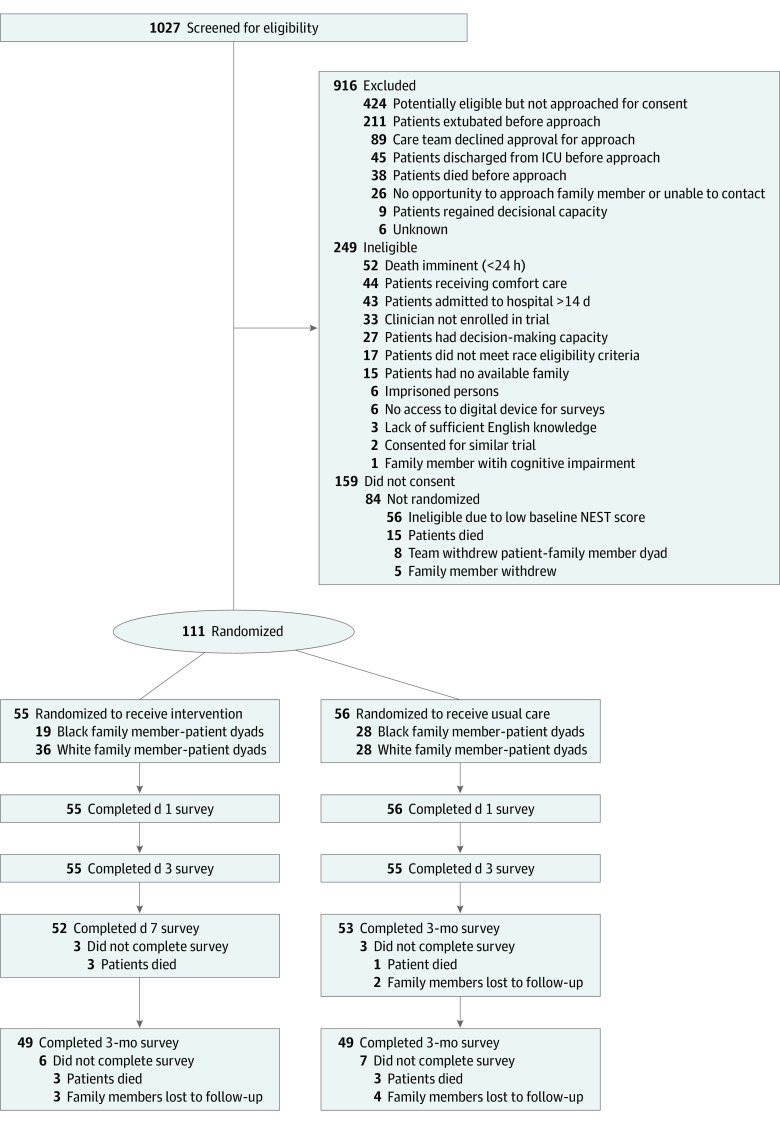
Screening, Randomization, and Analysis Flow Diagram This diagram shows the flow of all patient–family member dyads through the trial. ICU indicates intensive care unit; NEST, Needs at the End-of-Life Screening Tool.

From a total of 55 adult ICU physicians screened, 53 (96%) consented and 43 (78%) were randomized, of whom 37 (23 male physicians [62%]; 5 Asian physicians [14%]; 31 White physicians [62%]; 1 physician of another race [3%]) had patient–family dyads enrolled under their care (19 intervention physicians and 18 control physicians). Most of the physicians worked in a medical ICU (23 physicians [62%]) (eTable 1 in Supplement 2). Among 778 potentially eligible patients, 354 patient–family member dyads (46%) were approached for enrollment, 195 (55%) provided informed consent within a median (IQR) of 4.0 (3.0-6.0) days after ICU admission, and 111 family members with elevated NEST scores (57%; mean [SD] age, 51 [15] years; 96 women [86%]; 15 men [14%]; 47 Black family members [42%]; 64 White family members [58%]) were included in the intervention (55 family members) or control (56 family members) groups (Figure 1, Table 1, and eTable 2 in Supplement 2). Of the 111 patients (mean [SD] age, 55 [16] years; 66 male patients [59%]; 45 Black patients [41%]; 65 White patients [59%]; 1 American Indian or Alaska Native patient [1%]), 61 (55%) were cared for in medical ICUs (eTable 3 in Supplement 2). No patient–family member dyad received a palliative care consultation before enrollment. Follow-up was complete at 3 months for 98 participants (88%). See eTable 4 in Supplement 2 for more information on retention by treatment group and race.

**Table 1.** Patient and Family Member Characteristics

Characteristic	Patients or family members, No. (%)		
	Total (N = 111)	Intervention (n = 55)	Control (n = 56)
Family member characteristics			
Age, y			
Mean (SD)	51 (15)	50 (13)	52 (16)
Median (IQR)	52 (43-61)	51 (43-58)	54 (43-62)
Gender at birth			
Male	19 (17)	12 (22)	7 (13)
Female	92 (83)	43 (78)	49 (88)
Gender (self-described)			
Man	15 (14)	10 (18)	5 (9)
Woman	96 (86)	45 (82)	51 (91)
Race (self-described)			
Black or African American	47 (42)	19 (35)	28 (50)
White	64 (58)	36 (65)	28 (50)
Education			
Completed high school or below	20 (18)	12 (22)	8 (14)
Bachelor’s degree or other college-related education	68 (61)	35 (64)	33 (59)
Graduate or professional degree	23 (21)	8 (15)	15 (27)
Marital status			
Married or live with a partner	67 (60)	35 (64)	32 (57)
Separated or divorced	19 (17)	9 (16)	10 (18)
Widowed	4 (4)	1 (2)	3 (5)
Single	21 (19)	10 (18)	11 (20)
Financial distress			
After paying the bills, you still have enough money for special things that you want	45 (41)	19 (35)	26 (46)
You have enough money to pay the bills, but little spare money to buy extra or special things	44 (40)	24 (44)	20 (36)
You have money to pay the bills, but only because you have cut back on things	13 (12)	8 (15)	5 (9)
You are having difficulty paying the bills, no matter what you do	9 (8)	4 (7)	5 (9)
Relationship to patient			
Spouse or partner	50 (45)	24 (44)	26 (46)
Child	22 (20)	13 (24)	9 (16)
Parent	23 (21)	10 (18)	13 (23)
Brother or sister	11 (10)	6 (11)	5 (9)
Other	5 (5)	2 (4)	3 (5)
Employment			
Working full time or part time	74 (67)	33 (60)	41 (73)
Homemaker full time	7 (6)	4 (7)	3 (5)
Unemployed	4 (4)	2 (4)	2 (4)
Retired	19 (17)	10 (18)	9 (16)
Disabled	7 (6)	6 (11)	1 (2)
Insurance status			
Plan purchased via employer or union	57 (51)	23 (42)	34 (61)
Plan, self or family purchased	2 (2)	1 (2)	1 (2)
Medicare	22 (20)	11 (20)	11 (20)
Medicaid	12 (11)	8 (15)	4 (7)
TRICARE	7 (6)	7 (13)	0
Some other source	3 (3)	2 (4)	1 (2)
None	8 (7)	3 (5)	5 (9)
Relationship with ICU doctors			
Excellent	46 (41)	22 (40)	24 (43)
Good	43 (39)	21 (38)	22 (39)
Acceptable	16 (14)	9 (16)	7 (13)
Poor	6 (5)	3 (5)	3 (5)
ICU doctors discussed needs with family member in a formal meeting			
Yes	49 (44)	32 (58)	17 (30)
No	59 (53)	22 (40)	37 (66)
Missing	3 (3)	1 (2)	2 (4)
Expect hospital survival			
Almost certainly	56 (50)	29 (53)	27 (48)
Most likely	47 (42)	22 (40)	25 (45)
Probably or almost definitely not	8 (7)	4 (7)	4 (7)
Patient characteristics			
Age, y			
Mean (SD)	55 (16)	53 (15)	56 (16)
Median (IQR)	56.4 (44.3-65.8)	55.7 (44.0-62.9)	57.9 (46.0-67.6)
Gender			
Male	66 (59)	28 (51)	38 (68)
Female	45 (41)	27 (49)	18 (32)
Race			
American Indian or Alaska Native	1 (1)	1 (2)	0 (0)
Black or African American	45 (41)	18 (33)	27 (48)
White	65 (59)	36 (65)	29 (52)
Insurance status			
Medicare	41 (37)	19 (34)	22 (40)
Commercial	36 (32)	18 (33)	18 (32)
Medicaid	22 (20)	11 (20)	11 (20)
None	9 (8)	2 (4)	7 (13)
Veteran’s Administration	3 (3)	2 (4)	1 (2)
ICU			
Medical	61 (55)	34 (62)	27 (48)
Neurological	20 (18)	5 (9)	15 (27)
Surgical	17 (15)	10 (18)	7 (13)
Cardiac	13 (12)	6 (11)	7 (13)
ICU admission source			
Transfer from outside hospital	50 (45)	26 (47)	24 (43)
Emergency department	47 (42)	22 (40)	25 (45)
Hospital ward	9 (8)	5 (9)	4 (7)
Postoperative	4 (4)	2 (4)	2 (4)
Clinic	1 (1)	0	1 (2)
Primary ICU admission diagnosis			
Shock	16 (14)	11 (20)	5 (9)
Acute respiratory failure	53 (48)	25 (45)	28 (50)
Kidney failure	3 (2.7)	3 (5)	0 (0)
Liver failure	1 (0.9)	0	1 (2)
Acute neurological event or altered mental status	24 (22)	8 (15)	16 (29)
Trauma or postoperative	14 (13)	8 (15)	6 (11)
Acute Physiology and Chronic Health Evaluation II score, median (IQR) points	23.0 (1.0-28.0)	23.0 (18.0-28.0)	21.0 (16.0-27.0)
No. of chronic medical comorbidities, median (IQR)	1.0 (1.0-2.0)	1.0 (1.0-2.0)	1.0 (1.0-3.0)
Code status at ICU admission			
Full code	108 (97)	54 (98)	55 (98)
Do not attempt resuscitation	2 (2)	1 (2)	1 (2)
Missing	1 (1)	0	1 (2)
Palliative care trigger present			
No	72 (65)	32 (58)	40 (71)
Yes	39 (35)	23 (42)	16 (29)
Palliative care specialist consultation during hospitalization			
No	97 (87)	48 (87)	49 (88)
Yes	14 (13)	7 (13)	7 (13)
ICU physician reported discussing needs with family member in formal meeting			
Yes	NA	39 (35)	NA
No	NA	11 (10)	NA
Missing	NA	5 (9)	NA
Expected hospital survival by ICU physician, No			
Most likely	NA	30 (55)	NA
Almost certainly	NA	10 (18)	NA
Probably or almost definitely not	NA	10 (18)	NA
Missing	NA	5 (9)	NA
Relationship with family member reported by ICU physician			
Acceptable	NA	3 (5)	NA
Excellent	NA	17 (31)	NA
Good	NA	28 (51)	NA
Poor	NA	2 (4)	NA
Missing	NA	5 (9)	NA

Abbreviations: ICU, intensive care unit; NA, not applicable.

### Primary and Secondary Outcomes

#### Primary Outcome

**Figure 2.**
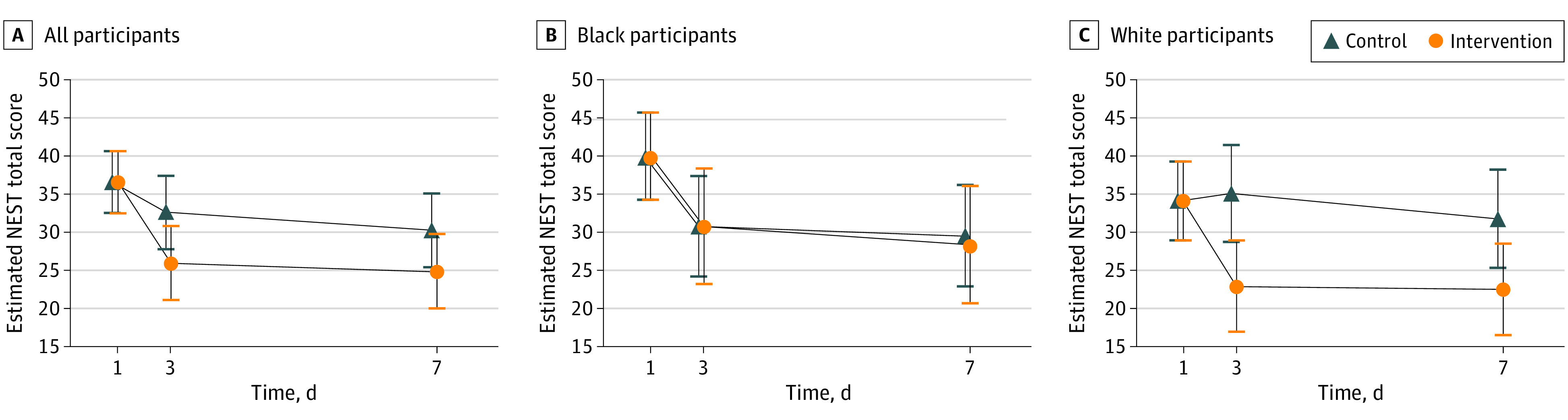
Primary Outcome by Treatment Group and Race Needs at End-of-Life Screening Tool (NEST) scores by group (vertical axis) are shown across time (horizontal axis). Circles and triangles represent mean estimated NEST scores, and vertical bars represent 95% CIs for the mean estimates. Estimated mean differences between intervention and control for the change in score between study days 1 and 3 and days 1 and 7 are as follows: all participants (A), control, −5.4 points (95% CI, −10.7 to 0.0 points; *P* = .05), and intervention, −6.6 points (95% CI, −11.9 to −1.3 points; *P* = .01); Black participants (B), control, −0.3 points (95% CI, −9.3 to 8.8 points; *P* = .96), and intervention, −1.4 points (95% CI, −10.7 to −7.8 points; *P* = .76); and White participants (C), control, −9.5 points (95% CI, −16.1 to −3.0 points; *P* = .005), and intervention, −12.5 points (95% CI, −18.9 to −6.1 points; *P* = .002).

Compared with usual care, the intervention group experienced a greater reduction in estimated mean NEST score at 3 days (estimated mean difference, −6.6 points; 95% CI, −11.9 to −1.3 points; *P* = .01) and 7 days (estimated mean difference, −5.4 points; 95% CI, −10.7 to 0.0 points; *P* = .05) (Table 2 and Figure 2). Additional details on NEST scores are shown by treatment group and physician (eFigure 4 in Supplement 2), by month of study (eFigure 5 in Supplement 2), by race (eFigure 6 in Supplement 2), and by individual NEST item (eFigure 7 in Supplement 2). A summary of needs reported by family members is available in eTable 5 in Supplement 2.

**Table 2.** Primary and Secondary Outcomes

Outcome	Score, mean (SD)		Intervention vs control, estimated difference, mean (95% CI)	*P* value
	Intervention (n = 55)	Control (n = 56)		
Primary				
Needs at End-of-Life Screening Tool score^a^				
Day 1	36.9 (19.0)	36.3 (17.4)	NA	NA
Day 3	26.5 (19.1)	32.3 (22.1)	NA	NA
Day 7	24.9 (19.5)	29.6 (20.2)	NA	NA
Change in score, day 3 vs day 1	NA	NA	−6.6 (−11.9 to −1.3)	.01
Change in score, day 7 vs day 1	NA	NA	−5.4 (−10.7 to 0.0)	.05
Secondary				
Patient Health Questionnaire-9 score (depression scale)				
Day 1	8.0 (3.5)	7.7 (3.6)	NA	NA
Day 3	6.9 (3.7)	6.4 (3.1)	NA	NA
Month 3	6.0 (3.4)	6.0 (3.4)	NA	NA
Change in score, day 3 vs day 1	NA	NA	0.3 (−0.5 to 1.1)	.48
Change in score, month 3 vs day 1	NA	NA	−0.2 (−1.4 to 1.1)	.79
Generalized Anxiety Disorder-7 score				
Day 1	9.2 (6.3)	8.4 (5.9)	NA	NA
Day 3	7.9 (6.1)	7.4 (5.4)	NA	NA
Month 3	6.1 (5.2)	5.3 (5.0)	NA	NA
Change in score, day 3 vs day 1	NA	NA	−0.2 (−1.4 to 1.0)	.73
Change in score, month 3 vs day 1	NA	NA	0.5 (−1.4 to 2.3)	.62
Posttraumatic Stress Syndrome Inventory score				
Day 1	26.2 (14.8)	24.5 (13.6)	NA	NA
Month 3	25.4 (15.7)	23.0 (13.9)	NA	NA
Change in score, month 3 vs day 1	NA	NA	0.6 (−1.0 to 2.1)	.47
Quality of Communication score				
Day 1	8.0 (2.5)	8.0 (2.4)	NA	NA
Day 3	8.9 (1.8)	8.4 (2.1)	NA	NA
Change in score, day 3 vs day 1	NA	NA	0.50 (−0.15 to 1.22)	.13
IPC scale: eliciting concerns score^b^				
Day 1, family members, No. (%)	46 (83.6)	47 (83.9)	NA	NA
Day 3, family members, No. (%)	49 (90.7)	44 (80.0)	NA	NA
Day 3 vs day 1, OR (95% CI)	NA	NA	2.53 (0.90 to 7.14)	.08
IPC scale: decision making score				
Day 1	3.6 (1.2)	3.7 (1.3)	NA	NA
Day 3	3.8 (1.1)	3.6 (1.3)	NA	NA
Change in score, day 3 vs day 1	NA	NA	0.3 (−0.1 to 0.7)	.15
IPC scale: discrimination score^c^				
Day 1, family members, No. (%)	4 (7.3)	7 (12.5)	NA	NA
Day 3, family members, No. (%)	3 (5.6)	7 (12.7)	NA	NA
Goal concordant care				
Day 1, No. (%)	37 (67.3)	50 (89.3)	NA	NA
Day 3, No. (%)	47 (85.5)	47 (83.9)	NA	NA
Day 3 vs day 1, OR (95% CI)	NA	NA	1.80 (0.60 to 5.20)	.29

Abbreviations: IPC, Interpersonal Processes of Care; NA, not applicable; OR, odds ratio.

^a^ Estimated from general linear model with repeated time, clinician random effect, constrained intercept, and centered stratification variables.

^b^ The IPC elicit concerns score was dichotomized at 5 vs less than 5.

^c^ The IPC discrimination score was dichotomized at 1 vs greater than or equal to 1. Due to the small number of events in each arm and time point, a longitudinal model was not fit to this outcome; no statistical test was conducted.

#### Secondary Outcomes

At baseline, family members reported mild symptoms of depression (mean [SD] PHQ-9 score, 8.0 [3.5] for intervention and 7.7 [3.6] for control), anxiety (mean [SD] GAD-7 score, 9.2 [6.3] for intervention and 8.4 [5.9] for control), and PTSD (mean [SD] PTSS score, 26.2 [14.8] for intervention and 24.5 [13.6] for control) (Table 2). There were no significant differences in symptom changes by group at 3 months. Change from baseline to study day 3 was not statistically significantly different between intervention and control groups in IPC-Elicited Concerns (odds ratio [OR], 2.53; 95% CI, 0.90 to 7.14; *P* = .08), and IPC-Decision Making (estimated mean difference, 0.3 points; 95% CI, −0.1 to 0.7 points; *P* = .15) subscales as well as Quality of Communication scores (estimated mean difference, 0.50 points; 95% CI, −0.15 to 1.22 points; *P* = .13) (Table 2). Although a numerically greater improvement in goal concordant care was reported by intervention family members compared with control family members, this finding was not statistically significant (OR, 1.79; 95% CI, 0.62 to 5.19; *P* = .29).

#### Participant Race and Outcomes

There was no difference in the change in NEST scores by treatment group among Black family members between baseline and study day 3 (estimated mean difference, −0.3 points; 95% CI, −9.3 to 8.8 points; *P* = .96) and study day 7 (estimated mean difference, −1.4 points; 95% CI, −10.7 to 7.8 points; *P* = .76) (eTable 6 in Supplement 2). Instead, a nearly equal reduction of almost 9 points was observed in both treatment groups (Figure 2). However, Black family members’ mean (SD) estimated baseline NEST scores (39.9 [22.8]) were higher than White family members’ scores (34.2 [13.4]), whereas postintervention mean estimated scores in both Black intervention and control groups were similar to White family member controls’ scores (eTable 6 in Supplement 2). In contrast, White intervention recipients experienced a greater reduction in NEST score compared with control at study day 3 (estimated mean difference, −12.5 points; 95% CI, −18.9 to −6.1 points; *P* < .001 vs estimated mean difference, −0.3 points; 95% CI, −9.3 to 8.8 points; *P* = .96) and day 7 (estimated mean difference, −9.5 points; 95% CI, −16.1 to −3.0 points; *P* = .005 vs estimated mean difference, −1.4 points; 95% CI, −10.7 to 7.8; *P* = .76). Overall, these results led to a significant race-by-group interaction effect between Black and White family members in the estimated mean difference between baseline and study day 3 (estimated mean difference, 12.2 points; 95% CI, 1.6 to 22.8 points; *P* = .02). This change in estimated mean difference in NEST scores was numerically greater for White family members compared with Black family members at day 7, but this finding was not statistically significant (estimated mean difference, 8.1 points, 95% CI, −2.7 to 18.9 points; *P* = .14). White family members also experienced a greater improvement between baseline and study day 3 in Quality of Communication scores (estimated mean difference, 1.0 points; 95% CI, 0.1 to 1.8 points; *P* = .02) compared with Black family members (estimated mean difference, 0.08 points; 95% CI, −1.2 to 1.4 points; *P* = .91) (eTable 6 in Supplement 2).

#### Patient Outcomes

Intervention patients were more likely than usual care patients to have a shorter median (IQR) duration of ventilation (9.0 [5.0-14.0] days vs 11.5 [6.5-20.5] days), to be extubated for comfort care (14 of 55 patients [25%] vs 8 of 56 patients [14%]), and to have a shorter median (IQR) ICU length of stay (17.0 [10.0-29.0] days vs 19.0 [11.5-29.0] days) (eTable 6 in Supplement 2). However, there were no treatment group mortality differences at hospital discharge (17 of 55 patients [31%] vs 16 of 56 patients [29%]) and 3 months (21 of 55 patients [38%] vs 19 of 56 patients [34%]).

## Discussion

The ICUconnect primary palliative care intervention reduced the severity of family members’ unmet needs during ICU care compared with usual care control in what is, to our knowledge, the first ICU-based randomized clinical trial targeting unmet palliative care needs. We observed a greater intervention effect among White family members than Black family members, and the severity of Black family members’ unmet needs was consistently higher than White family members’ unmet needs throughout the trial.

### Relevance to ICU-Based Palliative Care Delivery

Although palliative care interventions have improved quality of life and symptoms in outpatient and hospital ward settings,^42^ there is weaker evidence for their impact in ICU settings beyond sometimes reducing length of stay.^8^ Although important trials by Curtis et al^43^ and White et al^44^ included measures of quality of communication and patient-centeredness of care (and improved them), they also relied on adding trained interventionists to the ICU team. In contrast, this trial provides evidence that a simple automated primary palliative care intervention for families and ICU clinicians could help to expand the delivery of basic palliative care services at a time of staffing stress among the specialist palliative care workforce. The intervention’s focus on family member–reported needs is important because physicians have difficulty identifying unmet needs, especially when clinical characteristics are unrelated to the presence, severity, and type of needs.^29,45^

Although the intervention reduced palliative care needs across the ICU stay, there was no differential effect on psychological distress symptoms 3 months later. Although this could be due to participants’ relatively mild baseline symptoms, we recently found no association of 3-month psychological distress with several hospital-based, person-centered outcomes, including goal concordance of care and quality of communication.^30^ In fact, few ICU-based palliative care interventions have improved long-term psychological distress at a similar magnitude reported over 15 years ago in the landmark trial by Lautrette et al.^46^ Given the many confounding factors that accrue over time as the impacts of critical illness ripple forward, the suitability of long-term outcomes for assessing the effect of an ICU-based palliative care intervention is uncertain. In contrast, outcomes such as palliative care needs that are more temporally proximate to intervention and assess several domains of palliative care may better reflect intervention mechanisms and the time frame of greatest clinical importance to the patient, family member, and clinician.^14^ In addition to its responsiveness to change, the NEST can also serve as a screening tool given the poor predictive value of clinical characteristics,^29^ as well as a means by which to identify patients and family members with complex communication needs who may potentially be best served by specialists or to direct nonphysician multidisciplinary practitioners to those with specific needs aligned with their expertise.^32^

### Race and Outcomes

One explanation for the nearly identical improvement in outcomes for Black family members in both treatment groups (ie, intervention and control) is that there was an enhanced attention control effect among Black recipients. Past work^22,25,47^ has shown that physicians are less likely to inquire about psychosocial needs in conversations with Black families compared with White families. Therefore, the trial’s focus on systematically prompting family members from both treatment groups to consider their individualized needs may have been perceived by Black families as therapeutic and may have created more opportunities for all Black families to share and proactively address their needs with clinicians.^48^ Another explanation may be that Black families’ expectations for interactions with ICU teams are different from White families’ expectations because of negative past health care experiences.^49^

There was a greater intervention effect among White family members compared with Black family members. Although the intervention prompted ICU physicians to act upon family-reported needs, well-described mechanisms of interpersonal racism may have led to the differential provision of such support to Black and White families.^50,51^ For example, although the intervention may have helped the predominantly White ICU physicians to better recognize Black family members’ needs, their ability to address them may have nonetheless been suboptimal.^15,21,22,52,53^ This hypothesized mechanism is supported by improved family-perceived quality of clinician communication among White, but not Black, participants despite Black family members having more family meetings. These findings are also consistent with research documenting the association of both practitioner implicit bias toward Black patients and racially discordant interactions between clinicians and patients and/or family members (the norm for Black families in the ICU including the trial site) with lower quality communication and care.^21,53,54,55,56^ Nevertheless, these differences by race should be interpreted with caution given the relatively small sample size of Black participants. Given the underrepresentation of populations from racial and ethnic minority groups in clinical trials in general and ICU-based palliative care trials specifically,^7,57^ future studies with larger sample sizes of Black patients and individuals from other racial and ethnic minority groups are needed to better understand potential mechanisms of these findings as well how to develop or refine interventions such as our own to ensure equitable outcomes.

### Limitations

This study has limitations that should be noted. Because most of this trial was conducted during the early COVID-19 pandemic when hospital visitation was restricted for family members and research staff, fewer participants were enrolled than planned; despite the redesign of study procedures for virtual conduct, robust measures of intervention fidelity (eg, observation of family meetings) were infeasible, limiting an understanding of dose. Compounding this challenge was the trial’s cluster design in which participants could only be enrolled when a randomized ICU physician was on service. An additional consideration is our limited ability to understand how this trial’s results may have been affected by the heightened attention toward racism in America prompted by the contemporaneous murder of George Floyd and the widespread recognition of excess deaths among people from racial and ethnic minority groups during the COVID-19 pandemic.

## Conclusions

Compared with usual care, the ICUconnect primary palliative care intervention improved the burden of unmet palliative care needs among family members of critically ill patients. There was a greater intervention effect among White family members compared with Black family members. This promising intervention requires further testing and exploration of race-based effects in a larger clinical trial.

## Notes

Supplement 1.
Trial Protocol and Statistical Analysis Plan

Supplement 2. **eAppendix 1.** Eligibility Criteria **eFigure 1.** Study Flow **eFigure 2.** Mobile App Notification Strategy **eAppendix 2.** List of Questions Provided to Intervention Family Members **eFigure 3. **Screenshots From ICUconnect App **eAppendix 3.** Training of Family Members and Physicians **eAppendix 4.** Family Meeting Conduct **eAppendix 5.** Procedure for Managing Changes in ICU Attendings Occurring During Intervention Time Period for Family Members **eAppendix 6.** NEST Items **eAppendix 7.** SAS Code for Primary Outcomes **eTable 1.** Characteristics of ICU Attending Physicians **eTable 2.** Family Member and Patient Characteristics by Race **eTable 3.** Clinical Characteristics and Clinical Outcomes **eTable 4.** Consent and Retention by Race and Group **eFigure 4.** Primary Outcomes at Day 3 by Treatment Group and Physician **eFigure 5. **Graph of Baseline NEST Scores by Month of Study **eFigure 6. **Primary Outcomes at Day 3 by Treatment Group, Race, and Physician **eFigure 7.** NEST Item Changes Over Time **eTable 5.** Needs Reported by Family Members in Their Own Words **eTable 6.** Outcomes by Race and Study Group

Supplement 3.
Data Sharing Statement
