# Supplementary material for: Mobile Application–Based Communication Facilitation Platform for Family Members of Critically Ill Patients: A Randomized Clinical Trial
Source: JAMA Netw Open. 2024 Jan 4;7(1):e2349666. doi: 10.1001/jamanetworkopen.2023.49666 (PMC10767607; doi:10.1001/jamanetworkopen.2023.49666)
Supplement: Supplement 1. — Trial Protocol and Statistical Analysis Plan [file jamanetwopen-e2349666-s001.pdf]

DUHS IRB Application (Version 1.34)

General Information

***Please enter the full title of your protocol:**

Addressing Needs Among Intensive Care Unit Family Members

***Please enter the Short Title you would like to use to reference the study:**

REACH Study
* This field allows you to enter an abbreviated version of the Study Title to quickly identify this study.

Add Study Organization(s):

List Study Organizations associated with this protocol:

Primary Dept?	Department Name
◆	**DUHS** - Duke Default Department

Assign key study personnel (KSP) access to the protocol

*** Please add a Principal Investigator for the study:**

(Note: Before this study application can be submitted, the PI MUST have completed CITI training)

Cox, Christopher

3.1 **If applicable, please select the Key Study personnel: (Note: Before this study application can be submitted, all Key Personnel MUST have completed CITI training)**
*** Denotes roles that are not recognized in OnCore. Please select an appropriate role that is recognized in all clinical research applications (iRIS, OnCore, eREG, etc.)**

A) Additional Investigators, Primary Study Coordinator (CRC), and the Primary Regulatory Coordinator (PRC):

Al-Hegelan, Mashael
Co-PI
Docherty, Sharron
Co-PI
Haines, Krista
Co-PI
Harrison, Robert
Co-PI

B) All Other Key Personnel

Alkon, Aviel
Data Manager
Ashana, Deepshikha
Collaborator
Bermejo, Santos
Study Coordinator (CRC/CRNC/RPL)
Brewley, Destiny
Study Coordinator (CRC/CRNC/RPL)
Cupelli, Olivia
Study Coordinator (CRC/CRNC/RPL)
Dempsey, Katelyn
Study Coordinator (CRC/CRNC/RPL)
Fernandes, Priyanka
Study Coordinator (CRC/CRNC/RPL)
Frear, Allie
Study Coordinator (CRC/CRNC/RPL)
Gao, Xiaomei
Data Manager
Gu, Jessie
Sub-Investigator
Hepler, Bonnie
Study Coordinator (CRC/CRNC/RPL)
Johnson, Allison
Study Coordinator (CRC/CRNC/RPL)
Johnson, Kimberly
Sub-Investigator
Koch, Amie
Study Coordinator (CRC/CRNC/RPL)
Lowder, Yen
Study Coordinator (CRC/CRNC/RPL)
Manyara, Raha
Study Coordinator (CRC/CRNC/RPL)
Matsouaka, Roland
Statistician
McDowell, Brittany
Study Coordinator (CRC/CRNC/RPL)
Misiewicz, Remi
Study Coordinator (CRC/CRNC/RPL)
Olsen, Maren
Statistician
Parish, Alice
Statistician
Pratt, Elias
Sub-Investigator
Randolph, Schenita
Study Coordinator (CRC/CRNC/RPL)
Riley, Isaretta
Sub-Investigator
Wood, Heather
Data Manager
Yang, Hongqiu
Data Manager

***Please add a Study Contact:**

Bermejo, Santos
Cox, Christopher
Dempsey, Katelyn
Frear, Allie
McDowell, Brittany

The Study Contact(s) will receive all important system notifications along with the Principal Investigator. (e.g., The study contact(s) are typically the Principal Investigator, Study Coordinator, and Regulatory Coordinator.)

Oncore

Please select the Library for your Protocol:

This field is used in OnCore. Determines the Reference Lists, Forms, Protocol Annotations, Notifications, and Signoffs available for the protocol. Protocols that require reporting to the NCI (National Cancer Institute), must select the Oncology library.

- ( ) Oncology
- (•) Non-Oncology

Protocol Application Type

Select the type of protocol you are creating:

Please see additional criteria and information in the policy titled "Reliance on the IRB of Another Institution, Organization, or an Independent IRB" on the **IRB web site**.

- (•) Regular Study Application - Most common. The IRB will determine if the study is eligible for expedited review or requires full board review upon submission.
- ( ) Application for Exemption from IRB Review - Includes Exempt, Not Human Subject Research, & Not Research.
- ( ) External IRB Application - Any study using an external IRB as the IRB-of-Record.
- ( ) Trainee Research While Away from Duke - Research conducted by medical students overseen by the Office of Curriculum & other student/trainee research away from Duke.
- ( ) Individual Patient Expanded Access, Including Emergency Use - Use of an investigational product under expanded access, including emergency use of an investigational drug or biologic or emergency use of an unapproved device.

Conflict of Interest

Do any of the participating study investigators or other key personnel (or their immediate family/significant other) have a financial or intellectual interest in, or are receiving compensation from, the sponsor or the drugs, devices or technologies used in this research?

( ) Yes (•) No

Are any key personnel an inventor of any of the drugs, devices or technologies used in this research?

(•) Yes ( ) No

Have you filed an Inventor Disclosure Form?

(•) Yes ( ) No

Do any key personnel have or anticipate (within the year) any financial relationships (e.g., consulting,

speaking, advisory boards, patents, equity, options) that could be perceived to overlap or present a conflict of interest with the current research?

○ Yes ◉ No

Do any key personnel have a conflict of interest management plan or cautionary memo issued by DOSI-COI related to this research?

○ Yes ◉ No

Oversight Organization Selection

CRU (Clinical Research Unit) or Oversight Organization Selection:

Please select the CRU.

Medicine

The Clinical Research Unit that takes responsibility for this study.

- Please select **Medicine** as the CRU **only** if the PI is in one of these Divisions or Institutes: Endocrinology, Gastroenterology, General Internal Medicine, Geriatrics, Hematology, Infectious Diseases, Nephrology, Pulmonary, Rheumatology & Immunology, Center for Applied Genomics and Precision Medicine, Center for the Study of Aging and Human Development, Duke Molecular Physiology Institute.
- More information on CRUs can be found on the Duke Office of Clinical Research (DOCR) website, **http://docr.som.duke.edu**
- Questions concerning CRU selection should be directed to docr.help@dm.duke.edu.
- For questions about the Campus Oversight Organization, please visit **Campus Oversight Organization**.

List all Key Personnel on the study who are outside Duke:

- **Note:** You will also need to attach the documentation of Human Subjects Certification for each individual, if they have completed the certification somewhere other than Duke.
- **If outside key personnel will have access to Duke PHI, a data transfer agreement AND external site IRB approval (or IRB authorization agreement) will be needed.** See HRPP policy **Use of Research Data by Former Duke Students or Former Duke Faculty and Employees**
- In the panel below, "PHI" is Protected Health Information.

Entry 1

**Name**	
**Study Role**	
**Email Address**	
**Institution / Organization**	
**Will he/she have access to Duke P.H.I.?**	○ Yes ○ No
**Is he/she an unpaid volunteer at Duke on the study?**	○ Yes ○ No

Indicate the Protocol source below:

The protocol source is the author of the protocol. If the protocol is a joint authorship between multiple sources, select the primary author.

An IRB fee may be assessed for all research that is supported by for-profit entities and requires full board review. For additional information, see the **IRB fees section of the IRB web site**

- (selected) PI initiated
- Commercial / Industry (for-profit entity) initiated
- Federal Government initiated
- Cooperative Group Initiated
- Foundation (non-profit group) initiated
- Other

Sponsor and Funding Source

Add all funding sources for this study:

View Details	Sponsor Name	Sponsor Type	Contract Type:	Project Number	Award Number
⊟	National Institute on Minority Health and Health Disparities	Federal Government	Grant		

Sponsor Name:	National Institute on Minority Health and Health Disparities
Sponsor Type:	Federal Government
Sponsor Role:	Funding
**Grant/Contract Number:**	1U54MED012530-01
**Project Period:**	From: to:
**Is Institution the Primary Grant Holder:**	Yes
Contract Type:	Grant
Project Number:	
Award Number:	
Grant Title:	
PI Name:<br>(If PI is not the same as identified on the study.)	
Explain Any Significant Discrepancy:	

Is this a federally funded study?

(selected) Yes   No

Does this study have any of the following?

- Industry sponsored protocol
- Industry funded Duke protocol
- Industry funded sub-contract from another institution
- Industry provided drug/device/biologic
- SBIR/STTR funded protocol

○ Yes ◉ No

As part of this study, will any samples or PHI be transferred to/from Duke to/from anyone other than the Sponsor, a Sponsor subcontractor, or a Funding Source?

○ Yes ◉ No

Is the Department of Defense (DOD) a funding source?

○ Yes ◉ No

For Federally funded studies:

Is your funding subject to, and does it comply with, the funding agency's policy for data sharing?

○ Yes ◉ No

Enter the Grant Number or Other Federal Agency Proposal or Application Number:

1U54MED012530-01, 1R21NR016743-01A1

Note: The Federal Funding Agency ID Number is the Sponsor's grant number assigned to your project and available on your Notice of Award (example: R01HL012345).

If known, enter the SPS (Sponsored Projects System) number if applicable:

231956

In the Initial Submission Packet, attach the following:
(1) The entire grant, or an explanation of why a grant is not needed.
(2) NIH institutional Certificate form related to data sharing (if applicable).

The entire grant is needed so that it may be reviewed against the protocol for concordance.

Have you successfully synced your protocol to OnCore by clicking the 'Sync Data Over API' button at the top of this page?

Please verify that the protocol has been created in OnCore before submitting this application for PI Signoff.

◉ Yes, I synced my protocol to OnCore and verified it was successfully sent by logging into OnCore.
○ I may have forgotten! I'll click it again right now, just to be sure, and verify it was successfully sent by logging into OnCore.

Mobile Devices and Software

Does this study involve the use of a software or a mobile application?

◉ Yes ○ No

Please describe the following:

- The developer of the mobile app and how the app will be obtained.
- What PHI will be collected via the app.
- Where the data will be stored and who will have access to it.

The mobile application, which is a web app, not a native mobile app, was developed by local developer Smashing Boxes. The mobile application will collect various data from ICU family members and ICU clinicians. From ICU family members (study participants), the mobile app will collect demographic data from the family member including HIPPA identifiers such as their name, address, email address, phone number, gender, ethnicity, and race. The rest of the survey instruments that the family member would fill out do not include PHI, except for one free text question that asks family members to provide us with "any other need or concern." It is possible that the study participant could disclose PHI in that text box.

In order to collect ICU family member and clinician information, delegated study team members will fill out a study participant profile within the web app. This will enable the app to connect a study participant with a specific ICU physician. No one has access to this participant profile beyond the study team. Data collected here would include family member name, email address, phone number, patient name, patient hospital location (unit and bed number), and current ICU attending. This profile will only be viewable by the study team and can only be accessed via Duke Shibboleth two-factor authentication.

The ICU clinicians will be asked 3 questions, none of which contain PHI. Similar to the ICU family members, each ICU clinician will have a profile created in the web app. Information collected in the web app for the ICU clinician profile includes the clinician's name, email address, cell phone number, and service location.

All other data collection (including the chart abstraction, which contains PHI elements) will be pulled from the EHR (MaestroCare) directly into the secured Duke REDCap database by the study team, bypassing the mobile application.

All data gathered by the web app will be stored in a Duke DHTS hosted, secured, and maintained SQL database located behind Duke's PHI firewall. After the web app has been deployed to production, Smashing Boxes will not have access to this database, and only duke employees will have access (study team members, the study manager, the biostatisticians, and DHTS).

List all software, including third party (non-Duke) and mobile apps, that will be utilized for ascertainment, recruitment, or conduct of the research/project: (eg, MaestroCare, DEDUCE):

ICUconnect web app using a Duke DHTS hosted, secured, and maintained database (icuconnect.duke.edu): this is the mobile web application developed by Smashing Boxes.
Duke secured REDCap database: the REDCap database will house clinician and participant data that is both abstracted by the study team (patient information from the EMR) and also store completed surveys which are transferred from the web application via a secure API from the web application. Given the high volume of patient/family dyads screened and recruited on a daily/weekly basis, the study team has opted to utilize REDCap as a secure platform to track screened and enrolled patient/family dyads.
Twilio: this software is utilized for the purposes of enabling SMS messaging to be received by the participating clinician and/or family member.
Duke secured SMTP mail server: this software is utilized to send secure emails to participans (clinicians and family members) regarding their study participation and study visit reminders.
MaestroCare: the Duke EMR is utilized for screening, recruitment and data abstraction necessary for the conduct of the clinical research study.
OnCore: the CTMS is utilized to manage to protocol life cycle, including subject level management of patient/family dyads.
Duke Box: the secure server is utilized by the study team on a daily basis to maintain their shared, secured electronic master file for screening purposes. This document is updated and saved daily on DukeBox by the delegated study team members. Additionally, Duke Box is utilized as a secure storage location for subject level documentation, including audio files for Aims 3 and 4 associated with the qualitative analysis portion of the project.
Study Tracking (Duke DHTS-maintained database): this database, similar to a CTMS, is utilized to track subject level participation and study statuses throughout the life-cyle of the study. This is a secure system behind Duke firewalls and was created by a Duke Data Analyst (Avi Alkon) for research purposes only.
SureTypesALot: a Duke approved vendor will perform audio file transcription for the audio recorded interviews collected for Aims 3 and 4 associated with the qualitative analysis. The audio recorded interviews may contain PHI, such as the participant's name; however, it will be the responsibility of

SureTypesALot to change all names to pseudonyms. All interviewers are trained to avoid using the participant's name and instead refer to the individual as "you" or "your loved one" for example. Each audio file will be named and saved using the study ID. The linker file to the study ID is saved in a separate file available only to study key personnel. Audio file transcriptions will be stored securely in the study folder located on the secure DukeBox; the transcriptionist will access the recordings and upload the transcribed files in this location. Only delegated study team members will have access to this folder on DukeBox. As it relates to who will verify that all PHI has been removed and/or de-identified, it will be the responsibility of the transcriptionist to ensure that PHI has been removed and/or de-identified. The files will only be used for research purposes and will therefore only be accessible and usable by study team members. All data obtained from the audio transcriptions will be further affirmed to be de-identified by the study statisticians upon final analysis. As it relates to length of time the transcriptions will be stored – they will be stored until the study is closed and up to 6 years thereafter and then will be destroyed.

Multi-site Research

Is this a multi-site study?

○ Yes ◉ No

Complete for each site if Duke is the Primary grant awardee or coordinating center:

Entry 1

**Site Name:**	
**City:**	
**State/Province:**	
**Country:**	
	**Site Contact Information**
**Primary Contact Name:**	
**Primary Contact Phone:**	
**Primary Contact Email:**	
	**Site Details**
**Does the site have an IRB?**	○ Yes ○ No
**Site IRB approval expiration date:**	
**If date not provided, explanation of why:**	
**Has the site granted permission for the research to be conducted?**	○ Yes ○ No
**Does the site plan to rely on the DUHS IRB for review?**	○ Yes ○ No
**What is the status of the study at this site?**	○ Open<br>○ Closed

Site approval letters or site personnel lists:	Attach site approval letters, site closure letterS (if applicable), or site personnel lists in the Initial Submission Packet.

Research Abstract

Please type your Research Abstract here:

The Research Abstract should summarize the main points of your study in one paragraph. The following guidelines may help you:

1. Purpose and objective (1-2 sentences)
2. Study activities and population group (2-4 sentences)
3. Data analysis and risk/safety issues (1-2 sentences)

This 5-year project funded and conducted under Duke's REACH Equity Center, run by PI Kimberly Johnson. The blanket protocol number for the REACH Equity Center is Pro00090554.

The purpose of the study is to test and develop a web application (ICUconnect) that will improve racial disparities and unmet palliative care needs among ICU family members using a randomized clustered clinical trial (RCT). An ancillary study will be conducted simultaneously to determine if the presence of 'palliative care triggers' among patients is associated with greater unmet needs among patients' family members.

We anticipate that our total study population across both the RCT and ancillary studies will include, in total, 360 patients, 395 family members, and 60 physicians. The clinical trial group will consist of 160 family members of patients (80 white and 80 black) in Duke ICUs (medical, surgical, cardiac, cardio-thoracic and neurological, including DRH ICU). A total of 200 family members will participate in the ancillary study, 130 of whom will be enrolled as the control group in the clinical trial (including up to 30 who otherwise would have been deemed ineligible after T1 based on a low needs score i.e., NEST survey) and 70 others. 10 family members will complete app user testing alone and will not be trial participants (Aim 1 Usability) and up to 25 family members previously enrolled in aim 2 of the RCT and ancillary studies will be asked to participate in Aim 4 of the RCT (or Aim 3 of the ancillary study).

For the families enrolled in Aim 2 of the RCT or the ancillary project, study activities include the completion of 4 surveys: T1, T2, T3, and T4. Should a family member be enrolled into the RCT under an ICU clinician who has been randomized to intervention, a family meeting will occur between the randomized clinician and the family member to discuss the reported needs from T1. This family meeting will occur after T1 is completed but prior to T2 survey completion. Clinicians enrolled into the RCT and randomized to the intervention arm will be able to view the family needs data.

This study represents minimal risk. We have specific plans to address potential distress occurring during the study, as well as plans to protect against the potential loss of data confidentiality.

Research Summary

State your primary study objectives

The primary objective of the RCT is to compare the web app (ICUconnect) to usual care on unmet needs and perceptions of quality of care. The secondary objective is to determine the effect of the web app (ICUconnect) as an intervention on racial disparities in both unmet needs and in the quality of patient-centered care within the Duke University Hospital Intensive Care Units (ICUs) and Durham Regional ICUs. The ancillary study aims to understand how ICU patients' and their families' palliative care needs in an ICU setting evolve over time and develop a needs-targeted, ICU-based palliative care collaborative delivery model.

Using a multi-aim approach, we hypothesize that by conducting the RCT and ancillary studies simultaneously that we will be able to:

1. Optimize the usability of the ICUconnect web app intervention (Aim 1, RCT)
2. Find that the intervention (web app, ICUconnect) will improve:
    a. Unmet needs in both Black and White patients and families in the ICUs (Aim 2, RCT).

    b. Quality of patient-centered care in both Black and White patients and families in the ICUs (Aim 2, RCT).
3. Find that the intervention (web app, ICUconnect) will improve:
    a. Reduce racial disparities in unmet needs and quality of patient-centered care for patients and families in the ICUs (Aim 3, RCT)
4. Understand the type, severity and burden of the needs of patients and families and thereby develop a method to decrease psychological distress and develop a collaborative care delivery model for ICU clinicians (Aim 1 and 2, Ancillary)
5. Understand previously enrolled family members' unique experiences of having a loved one in an ICU—before or during COVID. (Aim 3, Ancillary and Aim 4 RCT).

State your secondary study objectives

N/A

Please select your research summary form:

Standard Research Summary Template

This is the regular (generic) research summary template which is required for all regular applications (unless your protocol fits under the other research summary templates in this category). Use of these instructions is helpful for ensuring that the research summary contains all necessary elements.

Standard Research Summary

Purpose of the Study

- Objectives & hypotheses to be tested

The purpose of the study is to test and develop a web application (ICUconnect) that will improve racial disparities and unmet palliative care needs among ICU family members using a randomized clustered clinical trial (RCT). An ancillary study will be conducted simultaneously to determine if the presence of 'palliative care triggers' among patients is associated with great unmet needs among patients' family members.

The primary objective of the RCT is to compare the web app (ICUconnect) to usual care on unmet needs and perceptions of quality of care. The secondary objective is to determine the effect of the web app (ICUconnect) as an intervention on racial disparities in both unmet needs and in the quality of patient-centered care within the Duke University Hospital Intensive Care Units (ICUs) and Durham Regional ICUs. The ancillary study aims to understand how ICU patients' and their families' palliative care needs in an ICU setting evolve over time and develop a needs-targeted, ICU-based palliative care collaborative delivery model.

The RCT will consist of 4 aims:

1. **Aim 1**: Optimize the usability of the ICUconnect app intervention in preparation for Aim 2's RCT.
    a. **Approach & goal**: We will optimize the usability of ICUconnect using focus group interaction-response testing. We will add key features based on a short series of iterative revisions with clinicians and families until it achieves 'excellent' usability (mean Systems Usability Scale score >85).
2. **Aim 2**: In a cluster RCT, determine the effect of the ICUconnect intervention versus usual care on unmet needs(primary outcome) and perceptions of the quality of patient-centered care(secondary outcome) in the ICU, both overall and within Black and White patients and families.
    a. **Hypothesis 2a**: As compared to usual care, the intervention will improve unmet needs in both Black and White patients and families in the ICU.
    b. **Hypothesis 2b**: As compared to usual care, the intervention will improve quality of patient-centered care in both Black and White patients and families in the ICU.
3. **Aim 3**: Determine the effect of the ICUconnect intervention versus usual care on racial disparities in both unmet needs (primary outcome) and in the quality of patient-centered care (secondary outcome).

a. **Hypothesis 3a**: Racial disparities in unmet needs will be reduced among patients and families in the ICU in the intervention group as compared to usual care.
b. **Hypothesis 3b**: Racial disparities in the quality of patient-centered care will be reduced among patients and families in the ICU in the intervention group as compared to usual care.

4. **Aim 4 (exploratory):** Using mixed methods (semi-structured interviews and quantitative process measures), characterize the impact of ICUconnect across a variety of family-and patient-implementation contexts, with a goal of understanding intervention mechanisms and family members' unique experiences of having a loved one in an ICU—before or during COVID.
    a. **Approach & goal**: We will integrate content analysis via a semi-structured interview with previously enrolled family members as well as quantitative family, patient, and care process outcomes. The content analysis will provide insight into ICUconnect' mechanisms of action, relate outcomes to unique case contexts, and help lead to future intervention optimization, replicability, and scalability. It is hoped that this aim may also provide meaningful information to field regarding the effects of COVID-19 on family members who have had a loved one in an ICU setting.

The Ancillary Study, conducted simultaneously with the RCT, will consist of 3 aims:

1. **Specific Aim 1**: To understand ICU patients' and families' palliative care needs in an ICU setting—and how they evolve over time.
    a. **Goal**: To understand the type, severity, and burden of need as measured with a framework of palliative care quality domains and derive clinically relevant 'need typologies' from these dat
        i. **Exploratory hypothesis 1a**: Family members with needs that remain unmet across the ICU stay will report greater 3-month psychological distress compared to those whose needs were met.
        ii. **Exploratory hypothesis 1b**: Family member need burden will be higher based on patient trigger status (present vs. absent), even after adjustment for sociodemographic and patient characteristics.
2. **Specific Aim 2**: Develop a need-targeted, ICU-based palliative care collaborative care delivery model.
    a. **Goal 2a**: Develop a provisional collaborative ICU-based palliative care model based on Aim 1's need typologies, clinician skill in addressing needs, clinician attitudes, and published guidelines. The algorithm-based model will display recommendations for addressing needs (ICU team or ICU team plus specialist palliative care) on a mobile app viewable on any electronic device.
    b. **Goal 2b**: Test the care model's acceptability among clinicians, using qualitative analysis of focus groups and quantitative satisfaction metrics. Model improvements will be made in an iterative process.
3. **Aim 3 (exploratory):** Using mixed methods (semi-structured interviews and quantitative process measures), characterize the impact of ICUconnect across a variety of family-and patient-implementation contexts, with a goal of understanding intervention mechanisms and family members' unique experiences of having a loved one in an ICU—before or during COVID.
    1. **Approach & goal**: We will integrate content analysis via a semi-structured interview with previously enrolled family members as well as quantitative family, patient, and care process outcomes. The content analysis will provide insight into ICUconnect' mechanisms of action, relate outcomes to unique case contexts, and help lead to future intervention optimization, replicability, and scalability. It is hoped that this aim may also provide meaningful information to field regarding the effects of COVID-19 on family members who have had a loved one in an ICU setting.

Background & Significance

- Should support the scientific aims of the research

Currently, the quality of ICU-based palliative care is highly variable. Patients suffer from unmet symptoms in a technology-focused setting, family members report poor quality communication and decision making, and clinicians struggle to identify needs and connect with families in a shiftwork environment. These factors disproportionately impact Black patients and their families. Compared to Whites, Blacks report lower satisfaction with the quality of ICU care and studies document disparities in multiple domains of patient-centered care in the ICU, including communication. Process barriers to improving the quality palliative care for this high-risk population include difficulty identifying high risk patients and families with

unmet palliative care needs, coordinating care by ICU teams, and engaging diverse family decision makers as partners in the care process. The field needs better ways to meet the palliative care needs of families in an ICU setting.

Figure 1 outlines the project's conceptual model.

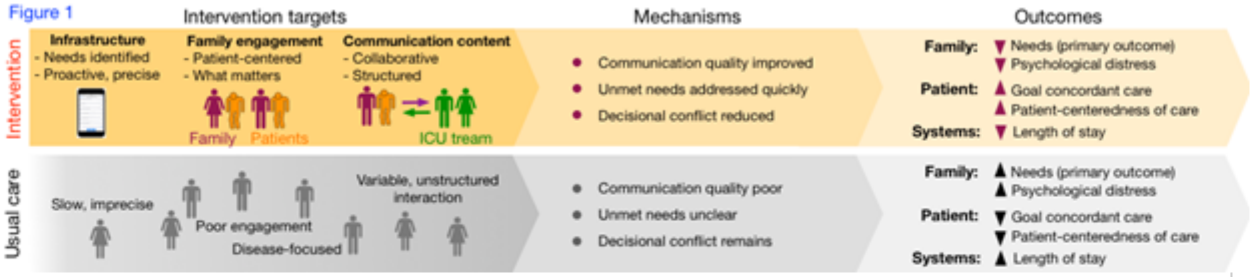

Design & Procedures

- Describe the study, providing detail regarding the study intervention (drug, device, physical procedures, manipulation of the subject or the subject's environment, etc.). Discuss justifications for placebo control, discontinuation or delay of standard therapies, and washout periods if applicable. Identify procedures, tests and interventions performed exclusively for research purposes or more frequently than standard of care. Include alternative therapies, concurrent therapies discontinued per protocol, risk benefit ratio, and use of tissue/specimens. Discuss monitoring during washout periods if applicable. Include brief description of follow-up, if any.

This study is a randomized clustered clinical trial (RCT) that will be conducted within the Intensive Care Units (ICUs) at Duke University Hospital (DUH) and Durham Regional Hospital (DRH). An ancillary study will be conducted simultaneously within the same locations as the RCT.

The RCT, as noted in the "Purpose of the Study" section above, will consist of 4 aims.

Aim 1 of the RCT will consist of three phases. The first phase will involve conducting 1-2 very informal focus groups in community gatherings at the Durham AME Zion Health Equity Advocates and Liaisons (HEAL) Partnership Meeting. During the meetings, the study team will obtain informal feedback surrounding the study surveys and the proposed subject handouts. Written informed consent will not be obtained because participants are aware of our participation in the meeting. The study team will preface the purpose of the presentation (and note that participants of the meeting do not have to participate or even remain in the room if they do not wish to do so). Feedback will be recorded by the study team via simple note taking. The study team will not record names of participants and no written questionnaires will be distributed. The study team will ask participants if they would be willing to participate in a more formal, future usability testing procedure and then record their contact information. The second phase of Aim 1 will involve web application (ICUconnect) improvements based on feedback received. These improvements include, but may not be limited to: simpler, secure login for Duke clinicians via shibboleth authentication, user-friendly interface, automated alerts to staff related to task completion, and enhanced dashboard views unique to the study role (e.g., CRC vs. clinical teams vs. family members). During this process, enterprise-level security features will be integrated to meet Duke's standard requirements. The third and final phase of Aim 1 is the evaluation of usability of the web application after phase 2 enhancements have been completed. During this phase a mixed methods approach will be utilized to complete usability testing. We intend to enroll 10-15 community-based laypersons (sampled geographically and racially). To complete user testing, each participant will receive an email with a brief explanation of the task (open ICUconnect, view information, complete ICUconnect survey) and a link to the System Usability Scale (SUS). Using this survey link, participants will have the opportunity to write free text feedback. No identifiers will be collected. Similarly, the study team will evaluate the physician-facing elements of ICUconnect among 8-12 purposively sampled ICU and palliative care clinicians. From both types of participants, data will be organized with the Nielsen usability heuristics model and revisions will be made to the web application as needed.

Aims 2 and 3 of the RCT will be completed simultaneously. The total targeted population for Aims 2 and 3 of the RCT are 240 enrolled patient/family dyads and a maximum of 60 randomized clinicians. To be considered "enrolled" for this study, a patient/family dyad must meet all eligibility criteria and receive a NEST score of $\geq$ 15.

For aims 2 and 3, the study team will conduct a randomized clustered clinical trial. Randomization occurs at the ICU clinician level versus that of the patient/family level. Clinicians are identified as eligible if they are the current attending on service for the particular ICU. Clinicians are randomized to one of the two arms based on the following:

1. Gender (male/female)
2. Clinical Experience (<10 years vs $\geq$ 10 years)
3. Discipline (medical vs. surgical)

Consented, enrolled clinicians are expected to complete the following survey(s):

- Baseline Demographics Survey (on the day of consent)

For those clinicians who are randomized to intervention, they will be required to complete the following:

- Review patient/family needs score and survey results within ICUconnect (at the completion of T1 by patient/family dyad)
- Schedule and conduct a family meeting (after T1 completion but before T2 completion)
- Review patient/family needs score and survey results within ICUconnect (at the completion of T2 by patient/family dyad)
- Complete the Clinician Survey via ICUconnect (after Family Meeting and completion of T1 and T2 by patient/family dyad).

Patient/family dyads will be identified at the time of ICU admission and approached for consent based on meeting study inclusion/exclusion criteria (see Selection of Subjects). Each patient/family member, if enrolled into the RCT will be asked to complete an ICUconnect profile and survey (T1). This survey is to be completed within 24 hours of consent to the study. Given that this study's intent is to address unmet needs, eligible participants must receive a NEST score of $\geq$ 15 (see Study Eligibility Workflow).

- If a participant receives a NEST score of < 15 and they are assigned to a randomized intervention clinician, the participant is considered a screen failure.
- If a participant receives a NEST score of < 15 and is assigned to a randomized control clinician, the participant is considered a screen failure for the RCT but is allowed to continue participation within the ancillary study.

Assuming that a participant receives a NEST score of $\geq$ 15, regardless of clinician randomization, the participant will continue with the following study procedures:

- Survey 2 (T2) to be completed within 3 days (0/+1 day) of T1 completion.
- Survey 3 (T3) to be completed within 7 days (0/+3 days) of T1 completion
- Survey 4 (T4) to be completed within 3 months (90 days, 0/+3 days) of T1 completion.

Should a participant receive a NEST score of $\geq$ 15 and be randomized to an intervention clinician, the participant will be contacted by the intervention clinician have a family meeting. Family meetings are to occur after the completion of T1 but prior to the completion of T2. Given the short timeframe between T1 and T2, the clinician can conduct the family meeting in a variety of settings: in the ICU at bedside, in a private conference room and/or location within the waiting room (at the choice of the participant), and/or over the phone.

In conjunction with Aims 2 and 3 of the RCT, the study team will simultaneously enroll patient/family dyads into the ancillary study. The ancillary study will include a total of 250 enrolled patient/family dyads. Eligible participants will be approached for consent at the time of the ICU admission. Participants enrolled into the ancillary study will be those participants whose current attending clinician is not enrolled in the RCT and/or their identified primary race and ethnicity is something other than White or African American /Black and Non-Hispanic. Participants enrolled in the ancillary study, like the RCT, must meet study eligibility criteria.

Participants enrolled into the ancillary study will complete an ICUconnect profile and then be asked to complete the following surveys:

- Survey 1 (T1) to be completed within 24 hours of consent.
- Survey 2 (T2) to be completed within 3 days (0/+1 day) of T1 completion.
- Survey 3 (T3) to be completed within 7 days (0/+3 days) of T1 completion
- Survey 4 (T4) to be completed within 3 months (90 days, 0/+3 days) of T1 completion.

Given that the targeted patient population involves individuals who are seriously ill and/or injured, the study team expects that there is a high likelihood that death could occur while the patient/family dyad's participation is ongoing. Should a patient pass away while study participation is ongoing, the following will occur:

- If the patient passes after the completion of T1 but prior to the completion of T2, the patient/family dyad will be withdrawn from the study.
- If the patient passes after the completion of T1 and T2 but prior to T3 completion, the patient /family dyad will remain eligible to continue study participation; however, the study team will be mindful and sensitive regarding approach and may skip T3. It is not considered a deviation if the family completes T3 prior to the study team enabling the "skip" option within ICUconnect.

- If the patient passes after the completion of T1, T2, and T3 but prior to completion of T4, the study team may wait 1 month before asking the family to complete the fourth and final study survey. It is not considered a deviation if the family completes T4 1 month late.

In regards to survey completion, it is understood, given the nature of the study design (self-directed participant survey completion), that surveys may not be completed in the specified protocol windows. The study team will provide participant education at the time of consent regarding the importance of completing surveys in a timely manner. In addition, the study team will follow-up with participants (patient /family dyads and clinicians) via phone, email, and in-person to complete study surveys and/or family meetings, as applicable.

The study consists of the following statuses for patient/family dyads:

- *Consented*: an individual(s) who meets all eligibility criteria and has voluntarily agreed to participate in the study (RCT or ancillary).
- *Enrolled*: an individual whom, after consent is obtained, completes the first survey.
  - Nte: if a participant is enrolled in the RCT study and receives a NEST score of < 15, he/she is deemed a screen failure and is therefore no longer enrolled. If a participant is enrolled in the ancillary study, he/she does not need to meet a NEST threshold for enrollment.
- *Screen Fail*: any participant who is enrolled into the RCT and receives a score of < 15.
- *Withdrawn by PI*: any participant who does not comply with study specific requirements (i.e. such as timely completion of study surveys, specifically T1 and/or T2) or is found to be by the principal investigator no longer appropriate for this study due to current state of being (physical, mental and /or emotional).
- *Withdrawn by Self*: voluntary withdraw of participant from study for any reason.
- *Completed*: any participant (RCT or ancillary) who completes 75% of study visits.

The study consists of the following statuses for clinicians:

- *Consented*: an individual clinician who is on service at the time of the eligible patient/family dyad admission and has agreed to voluntarily consent to the study.
- *Randomized*: a clinician who has been randomized to one of the two study arms:
  - *Interventin:* a clinician wh receives the patient/family dyad T1 and T2 survey results, conducts a family meeting to discuss results, and completes a clinician survey post family meeting and survey results review.
  - *Cntrol*: a clinician wh neither receives patient/family dyad T1 and T2 surveys nor conducts a family meeting to discuss results.

Given the nature of the targeted population under study and that the goal of this study is to address unmet needs and racial disparities among ICU family members, we do not anticipate adverse events and /or serious adverse events. However, it is possible that the patient/family dyad could become distressed in an ICU setting, especially as the patient becomes more ill and/or dies. As a result, the study team will monitor, document and report suicidal ideation (SI) as an Adverse Event (AE). The monitoring of SI is operationalized by the participant responding to one of the survey questions (PHQ-9; #9: *Thoughts that you would be better off dead or of hurting yourself in some way).* An automated alert is triggered and an email is sent to the Principal Investigator, the Study Manager, and the Clinical Research Coordinators. The PI will be the primary responsible party for addressing the SI alert; however, the Study Manager may also serve in the PI's absence. Both are trained on the Columbia Suicide Severity Rating Scale (C-SSRS).

A serious adverse event (SAE) for this trial would include a suicide attempt. Again, given the nature of the targeted population under study, it is not unexpected that the patient would experience hospitalization and subsequent ICU admission (or even repeat hospitalization, if discharged), therefore, the study will not document and/or report hospitalizations and/or deaths for the patients. However, if the enrolled family member and/or clinician were to be hospitalized and/or pass away, the study team would consider this an SAE and would document and report appropriately.

All protocol defined AEs and SAEs will be reported to the appropriate agencies (NIH) and the institutional review board, as appropriate and in line with guidelines and policies.

In addition, given that this study is short term, participant-driven, and survey-based, it is not anticipated that protocol deviations, violations, and or other unanticipated problems will occur at any great frequency. Should a protocol deviation, violation, or other unanticipated problem occur, the following reporting process will be adhered to: the study team will notify the PI of the error/issue(s). The event will be reviewed, documented and reported per institutional policies and NIH guidelines, as appropriate. As previously stated, this study does lend itself to the likelihood that the self-directed surveys will be completed outside of defined protocol window. These are not to be considered deviations and will not be reported as such given the likely frequency and benign nature of the event itself.

Characteristics of an Adverse Event(s)Relationship to Study Intervention

To assess relationship of an event to study intervention, the following guidelines are used:

1. Related (Possible, Probable, Definite)
    a. The event is known to occur with the study intervention.
    b. There is a temporal relationship between the intervention and event onset.
    c. The event abates when the intervention is discontinued.
    d. The event reappears upon a re-challenge with the intervention.
2. Not Related (Unlikely, Not Related)
    a. There is no temporal relationship between the intervention and event onset.
    b. An alternate etiology has been established.

Expectedness of SAEs

The Study PI will be responsible for determining whether an SAE is expected or unexpected. An adverse event will be considered unexpected if the nature, severity, or frequency of the event is not consistent with the risk information previously described for the intervention.

Severity of Event

The following scale will be used to grade adverse events:

1. Mild: no intervention required; no impact on activities of daily living (ADL)
2. Moderate: minimal, local, or non-invasive intervention indicated; moderate impact on ADL
3. Severe: significant symptoms requiring invasive intervention; subject seeks medical attention, needs major assistance with ADL

For Aim 4 (or Aim 3 for the ancillary study), delegated study team members will review the list of previously enrolled patient/family dyads and select individuals for recruitment based on their already collected information from study participation in aims 2 and 3 in the following categories:
-Study (R21 vs. ICUconnect intervention arm)
-Gender
-Trigger (present vs. absent)
-Race
-Age
-Medical vs Surgical ICU (place of eligibility and recruitment)
-NEST at T1 and T2 (responder vs. non-responder)
-Technology confidence
-Status (completed)

Families who meet the criteria listed above will be contacted via telephone and/or email and asked to participate in this portion of the project using the approved telephone and/or email consent template. Each individual will be asked to read, review, and sign/date an eConsent attesting to their willingness to participate.

As part of this aim, families who choose to participate will be asked to complete a 15 minute audio recorded interview in which the family member will be asked a series of questions pertaining to their loved one's hospitalization and ICU admission, the status of their loved one (alive or deceased), and then their overall experience related to their loved one's care in the ICU and their interactions with the clinical care team.

Selection of Subjects

- List inclusion/exclusion criteria and how subjects will be identified.

To identify eligible patient/family dyads for consent in both the RCT and the ancillary study, the study team will utilize the electronic medical record (EMR) known as EPIC for screening purposes. The study team will complete a daily review of the EMR, specifically reviewing each ICU unit and associated beds for patients who meet study criteria.

For both the RCT and the ancillary study, eligible participants are those patients who are at least at moderate risk of death or disability. In addition, while patients will not be excluded based on diagnosis, it is anticipated that most patients will predominantly be diagnosed with cardiorespiratory failure, stroke, trauma, and/or septic shock.

Eligible clinicians will be identified by the Principal and sub investigators. Clinicians must meet the criterion listed below. In addition, the selected clinician for consent and subsequent randomization should be scheduled to be on service at least 8 weeks per year (or the equivalent in terms of 12-hour shifts).

Inclusion Criteria (Patients, Pre-Consent)

1. ≥18 years of age
2. Patients must be on mechanical ventilation in a study ICU for ≥ 24 hours
    a. For those patients whose mechanical ventilation is interrupted for <72 hours, they will remain eligible for study participation.
    b. If a patient is intubated on the day of eligibility but then subsequently extubated the same day, the patient remains eligible for the study as long as consent and T1 are completed within 8 hours of extubation.
3. Death must not be imminent (< 24 hours)

Inclusion Criteria (Family Members, Pre-Consent)

1. One family member per patient is eligible.
2. The family member must be ≥ 18 years old.
3. The family member must be self-described as the individual (related or unrelated) who provides the most support and with whom the patient has a significant relationship.

Inclusion Criteria (Clinician, Pre-Consent)

1. Patients' bedside ICU attending physicians on the day of family consent are eligible.

Exclusion Criteria (Patients, Pre-Consent)

1. Patients who are admitted to an ICU at the index hospital >14 days will be ineligible for participation.
2. Patients who are on comfort care or are planned to undergo withdrawal of treatment within the following 48 hours of enrollment will be excluded.
3. Prisoners
4. Patients who have no known family or surrogate.
5. Patients who are transferred to a non-study ICU or non-study clinician will be excluded if they are enrolled into the RCT only.

Exclusion Criteria (Family Members, Pre-Consent)

1. Family members will be excluded if they lack English fluency or have other limiting factors that inhibit their ability to use the app (e.g. literacy, blindness, etc).
2. Family members will be excluded if they are not directly involved in decision making, are unreachable or unavailable.
3. Family members will be excluded if they are <18 years of age.
4. Family members will be excluded if they are unable to consent (e.g., dementia, cognitive impairment).
5. Prisoners.
6. For the RCT only: family members who self-identify as Hispanic and/or self-identify most with a racial group other than White or Black will be excluded.
    a. Ethnic and racial exclusions do not apply to the ancillary study.

Exclusion Criteria (Patient/Family Dyad Post-Consent)

1. Patient dies before T2 is completed.
2. Family members will be excluded from the RCT, but not the ancillary study, who have a very low need burden (NEST < 15) after the completion of T1.
3. For the primary RCT, participants are also excluded if the randomized physician leaves ICU service or is replaced by a non-participating ICU physician < 2 calendar days after family completes T1.

For Aim 4 RCT (or Aim 3 Ancillary Study) Only:
Inclusion Criteria (Family Member)
1. Must have been previously consented and enrolled in the ancillary and/or RCT study, meaning that all eligibility criteria noted above was met both pre and post consent.

Exclusion (Family Member)

1. Inability, in the opinion of the study team, to complete the single study visit.

Subject Recruitment and Compensation

- Describe recruitment procedures, including who will introduce the study to potential subjects. Describe how you will ensure that subject selection is equitable and all relevant demographic groups have access to study participation (per 45 CFR 46.111(a) (3)). Include information about approximately how many DUHS subjects will be recruited. If subjects are to be compensated, provide specific prorated amounts to be provided for expenses such as travel and/or lost wages, and/or for inducement to participate.

All eligible patient/family dyads, regardless of RCT or ancillary study, are recruited from within the participating ICU. Recruitment of eligible patient/family dyads can occur in 1 of 3 ways, depending on the patient/family dyad at the time of consent:

1. Traditional research introduction and in-person consent: the study team will approach a member of the clinical team who is facilitating in the care and communication with the eligible patient/family dyad. The study team will inform the clinical team member (typically the registered nurse in charge of the patient's room) that the patient/family dyad are eligible for participation in a research study. A brief explanation of the research study will be provided to the clinical team member. Given the sensitive nature of the targeted study population (at risk for death and/or disability), the study team will ascertain from the clinical care team member if it is an appropriate time to speak with the patient/family dyad. If the clinical care team member says that it is okay, the clinical care team member will be asked to provide a research introduction to the patient/family dyad. Assuming that a verbal "okay" to approach is provided, the study team will approach and provide a brief review of the study (verbally and/or utilizing the brief study infomercial) and ask if they would like to review the informed consent. Should the patient/family dyad be interested, the study team member will proceed with either eConsent or traditional paper consent, at the request and preference of the patient/family dyad.
2. Traditional research introduction and remote consent utilizing eConsent: in many instances family members of patients admitted to the ICU are not able to be present at the patient's bedside during normal business hours. In addition, many patients' family members are located out of state. The family members are still communicating with the clinical care team about their loved one after normal business hours or remotely (via phone, email and MyChart); however, this makes obtaining an in-person consent nearly impossible. As a result, when this type of scenario occurs, the study team will speak with the clinical care team and request a research introduction. Assuming that the family member provides verbal or written affirmation that they are okay to be approached by research, the study team will email the eConsent(s) to the family member and then follow-up via phone to review the informed consent with the family member in the same manner that they would review the consent if in person.
3. Utilization of the DUHS Recruitment and Engagement Policy: As noted above, many eligible family members of patients admitted to the ICU are not present at bedside. In addition, the clinical care team is often busy and have a number of different topics to discuss with the family members. As a result, research introductions for those families who are remote and/or visit the ICU after regular business hours often go uncompleted. To ensure enrollment for both the RCT and the ancillary studies remain successful, the study team would like to utilize an approved script to speak with family members without a traditional research introduction by a member of the clinical care team. Should this approach be utilized, the study team will utilize the eConsent to obtain informed consent unless the family member requests an in-person meeting for the purpose of consenting via paper. Prior to utilizing the policy, the study team will first review any potential participant's indication in MaestroCare to ensure they have not opted out of being contacted for research prior to contacting them. If the patient has opted out of being contacted for research no contact will be made utilizing the Recruitment and Engagement Policy.

Please note that for all scenarios described above, a research introduction may also be completed by the investigators of this study, as they all are routinely physicians on service and dually serve as members of the clinical care team. Research introductions for eligible patient/family dyads provided by investigators will be used as infrequently as possible and only when necessary. This will ensure that coercion to participate in the study is minimized as much as possible.

Eligible clinicians will be introduced to the research study by the investigators of the study. All eligible clinicians will be approached and provided a brief research introduction. If the clinician is interested in participation, he/she will provide verbal or written affirmation to the investigators. The investigators will then inform the study team of the clinician's interest in the study and confirm that it is okay to approach for research. The study team will then contact the clinician, due to their busy schedule, electronically and provide the eConsent (unless otherwise requested by the clinician, in which case paper consent would be utilized).

For the RCT, specifically, the study includes only those participants who self-identify as non-hispanic and either white or black. The purpose of this design is to determine racial disparities experienced among black patients and their families. However, the ancillary study is open to all races and ethnicities and will therefore ensure that the subject selection for this entire project is equitable and all relevant demographic groups have access to study participation.

To meet the statistical power needs for data analysis, the RCT intends to consent approximately 320 patient/family dyads to ensure that 240 patient/family dyads meet all study criteria and are subsequently enrolled. In order to enroll 240 patient/family dyads, 30-60 clinicians will be consented, enrolled and randomized to the study with a relatively equitable split between those clinicians assigned to intervention and those assigned to control.

In the same manner, the ancillary study intends to consent approximately 300 patient/family dyads to ensure that 250 patient/family dyads meet all study criteria and continue with subsequent study enrollment. The study team does not anticipate as high a screen fail rate among the ancillary study population as compared to the RCT given that participants do not need to meet a high needs threshold as defined by their completed NEST score at T1. No clinicians or other clinical care team members will be enrolled as part of this study.

All participants who consent and complete at least T1 will receive $20.00 for their time. For those participants who are enrolled into the RCT and remain eligible after T1, they will receive up to a total of $70.00 for the completion of all four surveys. For those participants who are enrolled into the ancillary study and remain eligible after T1, they will receive up to a total of $70.00 for the completion of all four surveys.

Compensation for participants is based on the completion of each study survey:

- T1: $20.00
- T2: $20.00
- T3 (mini survey): $10.00
- T4: $20.00

All compensation is received via check to the participant's provided mailing address on the completed IRB Data Disclosure Form (DDF).

Clinicians are not compensated for their study participation. This is to ensure and mitigate any potential research bias and/or coercion.

As noted above, Aim 4 RCT (or Aim 3 Ancillary), will recruit families from those individuals who already consented to the study for aims 2 or 3. Individuals will be approached via telephone and/or email and asked to consider study participation. They will be informed that research is 100% voluntary and that they are being asked to participate as they previously opted to participate in another portion of this study project. All willing participants will be asked to read, review, sign and date the eConsent attesting to their willingness to participate in the study.

Compensation will be provided to family members who choose to participate. Each individual will receive $20.00 for their time via check.

Consent Process

- Complete the consent section in the iRIS Submission Form.

Subject's Capacity to Give Legally Effective Consent

- If subjects who do not have the capacity to give legally effective consent are included, describe how diminished capacity will be assessed. Will a periodic reassessment occur? If so, when? Will the subject be consented if the decisional capacity improves?

Given the nature of the targeted patient population (i.e. those who are mechanically ventilated in an ICU), it is expected that they will not have the capacity to legally give effective consent. As a result, the family member (primary caregiver) and legally authorized representative (LARD) will be asked to consent on their behalf.

For those patients who have decision making capacity (DMC), they will not be excluded from participating. The family member (primary caregiver) of the patient will still be asked to consent and will be the primary responder for the study surveys; however, the patient who has DMC will be asked to consent on their own behalf to allow the study team to collect necessary medical information from their EMR for data analysis instead of the patient's LAR.

Study Interventions

- If not already presented in #4 above, describe study-related treatment or use of an investigational drug or biologic (with dosages), or device, or use of another form of intervention (i.e., either physical procedures or manipulation of the subject or the subject's environment) for research purposes.

The intervention under study is the novel web application known as ICUconnect. The primary purpose of ICUconnect is to develop an accessible, automated, and present application that proactively address needs (i.e. physical, mental, emotional, spiritual, and racial) and improves communication between ICU families, patients and the clinical care team members.

All participants, regardless of study enrollment into to the RCT or ancillary study, will be given access to ICUconnect and asked to complete surveys T1, T2, T3 and T4.

For those patient/family members enrolled into the RCT, they will specifically be assigned to a clinician who has been randomized to one of two study arms: "intervention" or "control". For those patient/family members who are randomized to an "intervention" clinician, the following will occur:

- T1 completed within 24 hours of consent.
  - Assuming a NEST scre of $\geq$ 15, the T1 survey results will be emailed r texted to the randomized clinician.
  - Clinician will review the T1 survey results and then, based n results, conduct a family meeting with the family member. The family meeting is to occur prior to T2.
- T2 completed 3 days after the completion of T1.
  - Clinician will receive T2 survey results and will be able t visualize the "change" in score post their family meeting. The purpose of this is to provide feedback to the clinician regarding how well they addressed the patient/family reported needs.
  - After the clinician has viewed the secnd survey results and "change" in score from the first survey to the second survey, the clinician will be asked to complete a 3 question survey regarding the patient's prognosis, the completion of the family meeting, and general impression of the family relationship.
- T3 completed 7 days after the completion of T1.
- T4 completed 90 days after the completion of T4.

For those patient/family dyads who are enrolled into the RCT and assigned a "control" clinician, they will receive "standard of care" as follows:

- T1 completed within 24 hours of consent.
  - Assuming a NEST scre of $\geq$ 15, the patient/family dyad remains eligible fr the RCT and continues with study enrollment. If they receive a score of < 15, they are no longer eligible for the RCT but will roll into the ancillary study.
- T2 completed 3 days after the completion of T1.
- T3 completed 7 days after the completion of T1.
- T4 completed 90 days after the completion of T4.

For those patient/family dyads enrolled into the ancillary study, they will not be assigned a clinician and will receive "standard of care" as follows:

- T1 completed within 24 hours of consent.
- T2 completed 3 days after the completion of T1.
- T3 completed 7 days after the completion of T1.
- T4 completed 90 days after the completion of T4.

For both the RCT patient/family dyads who are assigned to a “control” clinician and the ancillary patient /family dyads, their survey results will not be visible to a clinician and a family meet will not be conducted for research purposes.

Risk/Benefit Assessment

- Include a thorough description of how risks and discomforts will be minimized (per 45 CFR 46.111(a) (1 and 2)). Consider physical, psychological, legal, economic and social risks as applicable. If vulnerable populations are to be included (such as children, pregnant women, prisoners or cognitively impaired adults), what special precautions will be used to minimize risks to these subjects? Also identify what available alternatives the person has if he/she chooses not to participate in the study. Describe the possible benefits to the subject. What is the importance of the knowledge expected to result from the research?

Participants may experience some degree of stress due to the critical illness itself, and it is possible that they could experience anxiety when answering survey questions. When necessary, participants who experience psychological distress related to filling out self- report questionnaires will be referred for appropriate psychiatric or psychological care as described below.

Additionally, given the extreme stresses of the critical illness experience, participants may even endorse suicidal ideation (note the PHQ-9 has a suicidal ideation item). The study team will take the following measures to prevent any negative reactions as well as effectively manage any serious distress that occurs:

1. Participants will be told that they can discontinue survey completion at any time and revisit the survey when they feel they are able to complete it.
2. The study team will remain sensitive when discussing psychological distress among ICU family members.
    a. There is a potential risk for identifying underlying mental health issues through interactions, telephone calls, and survey responses. If issues, such as passive suicidal ideation or symptoms of depression, anxiety, or PTSD are suspected, the study team will alert the principal investigator. The principal investigator will follow-up with the patient/family dyad’s treating clinician and/or case manager within the ICU and ask for clinical follow-up, as needed.
3. The study team has developed a specific protocol to manage any study participant who expresses suicidal thoughts in person, by telephone, or via the app at any point during the study. The PI and other delegated study team members, as needed, are required to complete the online Columbia Suicide Severity Rating Scale (C-SSRS) training, a 30-minute interactive slide presentation followed by a question-answer session. Once completed, they are able to administer the C-SSRS. The C-SSRS is scale that can delineate high, moderate, and low-risk levels.

    Our Suicidality Response Plan can be activated in two main ways. First, study staff (coordinators, investigators, PI) may have in-person or telephone interactions that concern them. Second, the study data system will automatically detect any endorsement of the PHQ-9's suicidality item, sending an alert email in real time to the Study Manager and the PI. An example of this alert system in the context of using the Hospital Anxiety and Depression Scale (HADS) questionnaire in the CSTEP RCT is shown in the screenshot below.

    If the participant is deemed to be at high risk based on direct interaction or the PHQ-9 item response, then the PI and study manager will be notified immediately. Using the C-SSRS, the PI will then help to assess the participant to determine if they endorse active suicidal ideation. The PI will also determine of the participant is currently being treated by a mental health professional.

    If the patient is deemed not to be actively suicidal, they will be given a list of local mental health resources as follows:

    Duke: Call Emergency Psychiatry at 681-4410 or 681-1316, available 24 hours a day, 7 days a week.

    Backup: The National Suicide Prevention Lifeline - 1-800-273-TALK (8255) is a free, 24-hour hotline available to anyone in suicidal crisis or emotional distress.

    If the participant is considered to be actively suicidal then at least one of the following plans will be followed depending on the location of the participant for each of the following situations:

Situation 1. If the participant is with one of the study personal:

a. The study personnel will notify the PI immediately

b. The study personnel will either physically walk the subject to the emergency department, or call a Psychiatric Emergency services number.

Situation 2. If the participant is on the telephone:

a. The study personnel will notify the PI immediately.

b. The study personnel will stay on the telephone with the subject participant.

c. The study personnel will immediately contact 911 to initiate an on-site rescue if such action is clinically indicated.

d. The study personnel will stay on the telephone with the subject until EMS services have contacted the participant.

Situation 3. If there are any active suicidal concerns in any of the surveys (e.g., PHQ-9 above):

a. The study data system will notify the PI and Study Manager immediately.

b. Next, the study personnel will contact the participant by telephone.

c. The study personnel will stay on the telephone with the subject participant.

d. The study personnel will use a different telephone line to immediately contact 911 to initiate an on-site rescue if action is clinically indicated.

e. The study personnel will stay on the telephone with the subject until EMS services have contacted the participant.

If the participant is determined to be actively suicidal and requires immediate medical therapy, they will be withdrawn from the study.

Finally, there is a theoretical risk of loss of confidentiality of data given known limitations of data systems and human inputs. The study team will closely safeguard participant privacy regarding protected health and personal information. Study ID numbers, generated randomly at the time of enrollment, are linked to a separate Duke DHTS-maintained database, known as Study Tracking, that contains patient and family member names, as well as to a separate RedCAP subsystem that contains patient names and medical record numbers. Further, names, birthdates, telephone numbers, addresses, and medical record numbers are only viewable by the study team after authenticating into the webapp backend dashboard via Duke's shibboleth gate with two factor authentication. The webapp backend will store all study data on a Duke DHTS hosted, secured, and maintained SQL database. In addition, study data will be exported daily to a REDCap database via the REDCap API. Both databases store all data on secure Duke University servers with a sophisticated dual backup system. No PHI is made visible in any study participant interface. Study participants cannot view data via the webapp, only enter data (a 'one-way view') via one-time unique URLs to surveys. Participants access this one-way survey via PHI-free email or text links sent from the webapp backend system.

The study digital infrastructure consists of a central backend study management application, known as Study Tracking, hosted on a Duke secure server. This backend system manages three portals, one for study participant, one for ICU clinicians, and one for study management. Access to the study participant portal is through HTTPS and is designed to take in survey responses and serve up static content on the study and on asking questions while in the ICU via a proxy server that will serve as a hard wall between the web portal and the backend system. Access to the ICU clinician portal and the study management portal will only occur through Duke's Shibboleth two-factor authentication. The central backend system will house study data on a separate Duke DHTS hosted, secured, and maintained database, and will export study data daily to a separate REDCap database via the REDCap API. The central backend system will also utilize Duke's secure SMTP mail server to send out notifications.

Clinicians and intervention families will access the online elements through separate secure pathways hosted, monitored, and maintained by Duke University via the study webapp HTTP portals. Study staff will use their University credentials to login in a process identical in security strength to EHR login. The complete ICUconnect system is hosted on a secure Duke University and will be composed of a central webapp backend system, the separate HTTP portals (participant, clinician, study management) that will be served from the backend, a separate DHTS secured and maintained study database, and the study REDCap database.

Smashing Boxes has built technological solutions that preserve the privacy, confidentiality, and security of protected health information that may be part of health records or research datasets. All staff who work with sensitive data are required to complete appropriate HIPAA training with periodic updates, complete human subjects and data privacy training, comply with site IT Security Policies, and agree to the provisions of the University Rules of Behavior and Sanction Policy.

Any and all storage of confidential participant data on Smashing Boxes' hardware is strictly prohibited. For both the RCT and ancillary study, the web application and supporting backend architecture that Smashing Boxes developed is hosted internally on a secure Duke University server, established behind Duke's PHI firewall system. Thus, no patient data will be stored on Smashing Boxes' hardware and only on approved, secure University servers.

Study data imported into the REDCap database will be delivered via the ICUconnect web app backend via the REDCap API. The central REDCap database will run on a mirrored Duke University server system with automatic fail-over features, daily backups, and transaction logs. This system is physically located in a Tier II Data Center providing backup power sources, climate control, fire protection, and 24x7 surveillance. Audit logs will be reviewed routinely by Duke Health Technology Solutions staff to verify that security measures are operational. The servers are scanned twice weekly for vulnerabilities and are currently maintained at the highest level of vendor and CERT security recommendations. Data will never be shared outside the project unless authorized by the project leader as described in the Resource Sharing Plan. User authentication is based on user passwords as described earlier. Password creation requirements are in place to guarantee "strong passwords" as defined by the CERT security recommendations. The lead systems administrator is GIAC Security Essentials certified through May 2018.

To the study team's knowledge, this is the first study proposed to address the psychological distress of both ICU survivors using a telephone-based or mobile web platform-based behavioral intervention. The targeted population faces enormous but common disability as a result of critical illness and its sequelae. Therefore, the implications of this research, designed to mitigate this stress and suffering, are significant for the approximately one million such patients and their families treated each year in an ICU setting in the US.

It is hypothesized that that the intervention could greatly improve participants' well-being in the future, and we therefore believe that important knowledge could come of this proposed study. Specifically, the ICUconnect intervention may reduce psychological distress. Additionally, subjects in the control condition may experience similar or even greater benefits to those described for ICUconnect. At this time; however, direct benefit cannot be guaranteed.

Study involvement does place subjects at low risk for any adverse physical or psychological risk. Therefore, the potential benefits justify the minimal risks associated with the study.

Costs to the Subject

- Describe and justify any costs that the subject will incur as a result of participation; ordinarily, subjects should not be expected to pay for research without receiving direct benefit.

There are no costs to any participant (patient/family dyad or clinician) for participating in the RCT or ancillary study.

Data Analysis & Statistical Considerations

- Describe endpoints and power calculations. Provide a detailed description of how study data will be analyzed, including statistical methods used, and how ineligible subjects will be handled and which subjects will be included for analysis. Include planned sample size justification. Provide estimated time to target accrual and accrual rate. Describe interim analysis including plans to stop accrual during monitoring. Phase I studies, include dose escalation schema and criteria for dose escalation with definition of MTD and DLT.

Aim 1 Analyses: Aim 1 is designed to optimize ICUconnect usability. Verbal and free-text feedback from families and physicians, along with SUS scores examined across purposively sampled strata after each iteration until the goal is reached (mean SUS >85), will ensure app revisions adequately address the needs of low technology / low health literacy users. 14

Aim 2 Analyses:

General considerations: The primary analyses will be conducted on an intention-to-treat basis; clinicians and patients will be analyzed in the group to which they were randomized, regardless of clinician intervention adherence, using all available data. The main conclusions drawn from this trial will be based on the pre-specified hypotheses outlined below and will be tested with two-sided p-values at the standard 0.05 level. For all study outcomes, we will interpret differences between groups over time with reference to prior literature regarding clinically meaningful changes. Results from exploratory analyses will be interpreted with appropriate consideration for their exploratory nature. Statistical analyses will be performed using the latest release SAS for Windows (Cary, NC) and R/Rstudio.

Approach to statistical models: The goal of the primary hypothesis (2a) is to determine the efficacy of the ICUconnect intervention versus usual care on reduction in unmet needs from T1 to T2. Additionally, we are interested the intervention effect within Black families and White families separately. Unmet needs will be assessed at T1 and T2 with the total NEST score, a continuous, normally distributed measure. Because each family member is surveyed at multiple time points and the same ICU physician sees several patients, outcome variable measurements will not be independent. We, therefore, plan to use hierarchical linear models as our primary analytic strategy because they appropriately account for the multiple types of correlation inherent in our study design.45 The hierarchical linear model will have the following form: Yijk= b0+ b1(T2) + b2(T2*intk) + b3(racek)+ b4(servicetimek) + b5(ICUtypek)+ bik+ ck+ eijk, where Yijkis the total NEST score for family iat time jand physiciank. In this model, T2and intare indicator variables for the post-treatment and intervention group, respectively. Additionally, we include indicators for the randomization stratification variables. The multiple levels of correlation are captured via the physician-level random effect, ck, and the family-level random effect, bjk. Both random effects and the residual error (eijk) are assumed to be independent and normally distributed. PROC MIXED in SAS (SAS Inc., Cary, NC) will be used to fit the hierarchical linear models and test the primary hypothesis (2a). Specifically, if b2is negative and significantly different than zero, this provides evidence families in the intervention group have reduced unmet needs as compared to usual care group families. The secondary outcome, quality of patient-centered care, is also a continuous measure assessed at T1 and T2. A similar hierarchical linear model will be used to test Hypothesis 2b; in this model, if b2is positive and significantly different than zero, this provides evidence families in the intervention group have improved quality of patient-centered care as compared to usual care group families. Both analyses for Hypotheses 2a and 2b will be repeated within Black and White patients separately, with the goal to ensure that the intervention is effective in both subgroups (as an overall treatment effect can sometimes mask important heterogeneity). Aim 3 addresses relative differences in the intervention effect on reducing disparities.

Aim 3 Analyses: A hierarchical linear model will again be the primary modeling strategy for the Aim 3 analyses. For this Aim, however, the model will include patient-race interaction terms and will have the following form: Yijk= b0+ b1(Blacki) + b2(T2) + b3(T2*Blacki) + b4(T2*intk) + b5(T2*Blacki*intk) + b6 (racek)+ b7(servicetimek) + b8(ICUtypek)+ bik+ ck+ eijk, where Yijkis the NEST score for family iat time jand physician k. In this model, Blackiis the indicator variable for whether a patient is Black (value of 1) or White (value of 0). Racial disparities in unmet needs at baseline are represented by the estimated value of b1. That is, the estimated baseline mean for White families is b0 and the estimated baseline mean for Black families is b0 + b1, so if the estimate of b1is positive, this indicates that prior to the intervention, Black patients have more unmet needs than White patients. Post-treatment, the Black-white mean difference among usual care group families is BWuc = (b0 + b1+ b2 + b3) – (b0 + b2), and among intervention group families is BWint = (b0 + b1+ b2 + b3 + b4 + b5) - (b0 + b2+ b4). Hypothesis 3a will be tested by the difference between BWint and BWuc, indicating that racial disparities unmet needs are reduced in the intervention group as compared to the control group. The estimated difference, p-value, and 95% confidence intervals will be calculated via estimate statements in PROC MIXED. Additionally, estimated values and confidence intervals will be calculated for BWint as compared to b1 to quantify the magnitude of reduction from baseline. A similar hierarchical linear model will be used to examine the reduction in racial disparities of quality of patient-centered care and test Hypothesis 3b.

Missing Data: The survey data may contain missing values in any of the clinician-level and patient-level variables due to drop-out, death, or item non-response. Our primary analysis technique, hierarchical models, allow for unbalanced or incomplete data and will be fit with maximum-likelihood methods to preserve the missing at random assumption. Additionally, we will thoroughly explore reasons for dropout, and depending upon the type and scope of missing data, variables may be multiply imputed as recommended by guidelines.46Note that if needed, we will utilize imputation methods that account for the multiple levels of correlation inherent in the clustered data structure.

Aim 4's mixed methods analyses will explore and describe user experiences with the intervention to understand both mechanisms of action and how outcomes may be related to unique case contexts. Additionally, Aim 4 intends to understand the effects COVID-19 may have had on the family member and

their loved one's ICU admission as it relates to their needs. This knowledge can guide future intervention optimization through enhanced personalization, replicability, and scalability. Interview transcripts will be analyzed using a content qualitative analysis technique that combines structural (e.g., intervention component, care process barriers) and magnitude coding (e.g., theme intensity) with inductive coding (e.g., variations in outcomes not captured by instruments).50 Drs. Docherty and Cox will separately code the same 5 family members, discuss the generated codes to create an initial code book by consensus. Next, they will each code 5 different interviews with further refinements made to the code book which will then be used by the investigators to code remaining 15-20 family member transcripts. While the final code book will be organized into categories used to generate themes of mechanistic and process elements from across all participants, within-case themes generated from family member and clinician interviews will be used to write case profiles. Case profiles will merge data from case context descriptors (family and clinician race, patient illness, etc), within-case qualitative themes, and quantitative process measures. A visual 'ethnoarray' matrix will be constructed to display key case findings in relation to every other case. The ethnoarray, loosely adapted from a graphical heat map approach, is a powerful way to present complex data and is a flexible method for blending narrative and quantitative data to facilitate the discovery of patterns, relationships and understand the contextual richness of the data. These procedures will enhance comparative analyses and theoretical interpretation. For example, case comparisons could be made by needs (needs met vs. needs unmet) or patient-centeredness of care (high vs. low). Process tracing will be used to explore how particular case outcomes (e.g., need, distress, goal concordance) may have been related to intervention components (e.g., needs assessment, family meeting) and to their effect on barriers in our conceptual model (Figure 1). Dr. Docherty will use ATLAS.ti qualitative data analysis software for analyses.

Power and sample size considerations. For Aim 1, to reach a target of 'excellent' usability (mean SUS score >85), we expect 1-2 iterative revision cycles of 8-15 family members and 8-10 clinicians—a sample similar in size to that needed to reach thematic saturation in qualitative analysis (e.g., the 30 patient-level cases (total n=90) we expect will allow us to recognize theme saturation in Aim 4). The effect of interest for Aims 2 and 3 is essentially the T1 – T2 difference between the intervention and usual care groups. Aim 2 focuses on the overall difference and the difference within Black and White patients separately, while Aim 3 focuses on the difference within Black families compared to White families. Therefore, we base our sample size calculations on the mean difference score using tests for two means in a cluster randomized design. The sample size requirements are greatest for Aim 3; as discussed by Leon and Heo (2009), the needed sample size for the family race by intervention group interaction is 4 times that needed for the overall test. Based on preliminary studies, the standard deviation of the change in NEST score is estimated as 12 units and a reasonable range of intraclass correlation coefficients is 0.01 to 0.1. For all calculations, the type I error is 5% and power is 80%. With a sample size of 160 families (4 per each of 40 physicians, 20 per treatment arm), we will be able to detect differences of 4.0 to 5.0 NEST units for Hypothesis 2a in the overall test, 5.4 to 6.1 for Black or White patients separately, and 7.6 to 8.0 units for Hypothesis 3a. For Aim 4, we expect that up to 25 cases comprised of family members will be sufficient to reach informational redundancy—the point at which no new themes emerge.

Ancillary study: Needs and triggers: improving intensive care unit-based palliative care deliveryStatistical methods:

Aim 1: The primary goal of the cohort study (Aim 1) is to derive 3-5 clinically rational palliative care need typologies, or subgroups with a phenotypic homogeneity of need severity and type, to inform Aim 2's derivation of a care delivery model. These analyses will be conducted within the framework of latent class analysis (LCA), a statistical methodology that uses contingency table analyses to discern response patterns across a series of categorical variables. LCA will be applied to the dichotomized (high vs. low need) NEST item scores to identify latent (i.e., unobservable) subgroups of need classes within family members at T1 via maximum likelihood estimation (SAS PROC LCA). Study investigators will iteratively develop more fully formed needs typologies resulting from these analyses in a series of face-to-face investigator meetings using a decision-rule process. We will provisionally classify need typologies as 'simpler' (ICU team can address) or 'complex' (specialist care advised).

Exploratory hypotheses: Hypothesis 1aexplores the potential value of a novel intervention target—'unmet needs,' defined as a NEST summary score at T2 that is greater than T1 for those completing both interviews. This hypothesis will be confirmed if the unmet needs coefficient is statistically significant after regressing PHQ-9 scores at T4. Hypothesis 1bwill tested via a multivariable regression model with NEST summary score as the outcome and trigger status (present / absent) as the coefficient of interest, adjusted for sociodemographic and clinical variables. We anticipate < 10% missing data between T1 and T2.

Aim 2: This Aim's primary goal is to develop a provisional collaborative model of ICU-based palliative care using a mixed methods approach in which qualitative focus group data from family members, self-reported skills confidence data from clinicians, and expert guidelines will be integrated with Aim 1's need typologies. Step 1: validate needs typologies. 3 focus group interviews with family members purposively sampled from each of Aim 1's needs typologies subgroups will be conducted to validate the needs typologies in participants' own voices, verify the complexity of needs, and allow us to look beyond socioemographic variables for other common factors shared by subgroup members. Transcripts will be

analyzed using a content analysis technique that will combine structural and magnitude coding (a deductive form using a set of codes derived from the needs domains) with inductive coding (which identifies text of meaningful units and creates a label for the new category). Drs. Docherty and Cox will separately code an initial set of 5 interviews, discuss generated codes, and use a consensus procedure to create an initial code book, subsequently coding the remaining interviews. Final categories of codes will be used to generate themes. Step 2: assess confidence vs. expected skills. We will next survey 30 ICU physicians and 55 ICU nurses to evaluate their attitudes about and confidence in addressing each of the NEST need domains using 10-point Likert scales; scores >7 will reflect 'high' confidence. Step 3: assess acceptability. We will conduct 2 focus groups of 8-10 clinicians sampled purposively by stratifying across reported confidence levels (low vs. high), job type (physician vs. nurse), and discipline (ICU vs. palliative care). Clinicians will provide feedback on the clinical use of the derived need typologies. We anticipate discussions about how to sensibly use need type (i.e., NEST domains), need burden (i.e., NEST summary score), and need change over time (T2 > T1 NEST scores) to facilitate triage. Step 4: finalize model. Using data from Steps 1-3, investigators, expert Advisory Committee, and consultants (see letters of support), site palliative care and ICU leaders, and past guidelines relevant to expected basic palliative care skills, we will finalize the care model using an iterative, consensus-building approach.

Aim 2's secondary goal is to optimize model acceptability. Dr. Cox will facilitate 2-3 focus groups of 8-10 clinicians diverse in ICU type and role; a number sufficient to hold an active discussion and allow each participant to be heard. We will assess model acceptability using open-ended feedback and quantitative satisfaction metrics. Our targets will be a mean CSQ-8 score >15, with no scores < 5. A series of iterative revisions and retesting will be performed until the acceptability target is met. 'Member checking' of all past clinician participants will be performed using a 5-item Likert-scaled approval rating for the final model.

Aim 3's mixed methods analyses will explore and describe user experiences with the intervention to understand both mechanisms of action and how outcomes may be related to unique case contexts. Additionally, Aim 3 intends to understand the effects COVID-19 may have had on the family member and their loved one's ICU admission as it relates to their needs. This knowledge can guide future intervention optimization through enhanced personalization, replicability, and scalability. Interview transcripts will be analyzed using a content qualitative analysis technique that combines structural (e.g., intervention component, care process barriers) and magnitude coding (e.g., theme intensity) with inductive coding (e. g., variations in outcomes not captured by instruments).50 Drs. Docherty and Cox will separately code the same 5 family members, discuss the generated codes to create an initial code book by consensus. Next, they will each code 5 different interviews with further refinements made to the code book which will then be used by the investigators to code remaining 15-20 family member transcripts. While the final code book will be organized into categories used to generate themes of mechanistic and process elements from across all participants, within-case themes generated from family member and clinician interviews will be used to write case profiles. Case profiles will merge data from case context descriptors (family and clinician race, patient illness, etc), within-case qualitative themes, and quantitative process measures. A visual 'ethnoarray' matrix will be constructed to display key case findings in relation to every other case. The ethnoarray, loosely adapted from a graphical heat map approach, is a powerful way to present complex data and is a flexible method for blending narrative and quantitative data to facilitate the discovery of patterns, relationships and understand the contextual richness of the data. These procedures will enhance comparative analyses and theoretical interpretation. For example, case comparisons could be made by needs (needs met vs. needs unmet) or patient-centeredness of care (high vs. low). Process tracing will be used to explore how particular case outcomes (e.g., need, distress, goal concordance) may have been related to intervention components (e.g., needs assessment, family meeting) and to their effect on barriers in our conceptual model (Figure 1). Dr. Docherty will use ATLAS.ti qualitative data analysis software for analyses.

Power calculations and sample size: Based on Dziak's series of simulation studies examining statistical power in latent class models, 200 family members will provide > 80% power to detect a 3-class vs. a 2-class latent class model. Based on data from previous studies (estimated SD=12, type-I error of 5%), enrolling 184 family members (92 met/unmet needs) will provide 80% power to detect a 5-point difference. We conservatively plan to enroll 200 family members to protect against deviations from our assumptions and to account for dropout >25%.

Data & Safety Monitoring

- Summarize safety concerns, and describe the methods to monitor research subjects and their data to ensure their safety, including who will monitor the data, and the frequency of such monitoring. If a data monitoring committee will be used, describe its operation, including stopping rules and frequency of review, and if it is independent of the sponsor (per 45 CFR 46.111(a) (6)).

The study team will report all applicable adverse and serious adverse events, protocol deviations, protocol violations, and other unanticipated problems as defined by the Study Design & Procedures section and per Institutional Review Board (IRB) and NIH policies. In addition, all adverse and serious adverse events, protocol deviations, violations and other unanticipated problems will be reported as part of the semi and annually progress reports in the non-competitive and competitive renewals.

Dr. Cox will supervise this study at all times but will be in close and frequent contact with other investigators, including Dr. Olsen, the study biostatistician and the chief data manager. Investigators will adhere to established federal and institutional patient safety and protection guidelines. To assure data accuracy, Drs. Cox and Olsen will review the computer data files on a monthly basis. Additionally, coordinator staff will process the RedCap database routinely to search for errors and generate basic reports for dissemination at regular meetings. Protocol compliance will be reviewed during weekly meetings between clinical research coordinators and Dr. Cox.

Aim 2's RCT will be supervised by a single independent central DSMB due to its interventional nature. The DSMB will include professionals with significant experience in clinical trials, mind-body interventions, and biostatistics who are not directly involved in the study, its interpretation, or any study institution. DSMB members will be chosen with the assistance and approval of the NIH. The main responsibilities of the DSMB will be to (a) assess for the presence of potential harms and unintended consequences of the intervention, (b) ensure the validity and integrity of the data, and (c) make recommendations to the NIH about whether the study should be continued without modification, continued with modification, or terminated. The initial DSMB meeting will occur before the initiation of subject enrollment for the purpose of updating members on the study, ensuring agreement on the review process, and establishing the review methodology and procedures. The first DSMB data review will then occur either after the first 10 patients (5 per treatment group) have been enrolled or enrollment has occurred for 3 months, whichever is observed first. Thereafter, the DSMB will review cleaned data pulled from the RedCap database system every six months during enrollment and will prepare a report with any recommendations within the following month. While this is an intensive DSMB engagement schedule, the short enrollment period will demand greater oversight.

The specific study functions and outcomes that the DSMB will review at each meeting include: dropout rate, randomization rate, NEST, PHQ-9, GAD-7, and Post-Traumatic Stress Scale (PTSS) scores. The primary safety measures will be Adverse Events reports and post-intervention PHQ-9 scores. Other items reviewed by the DSMB at each meeting will include: (a) data quality, completeness, and timeliness; (b) performance of the Duke center; (c) adequacy of compliance with goals for recruitment and retention, including women and minorities; (d) protocol adherence; and (e) presence of factors that could adversely affect study outcome or compromise data confidentiality.

During the review process, formal statistical tests for examining the differences in Adverse Event or outcome rates between study groups may be performed under DSMB supervision if requested. However, this is unlikely given the exploratory pilot study design. For differences in study dropout rates, appropriate changes to the protocol will be made by investigator consensus after DSMB member input. Additionally, the DSMB may request a formal statistical assessment if a suspicious increase in PHQ-9 score is observed in either group. If the intervention group is found to have either a statistically significant increase in PHQ-9 score, the DSMB scope of action will include recommendation for cessation of the trial. Dr. Olsen, the biostatistician, will oversee any DSMB statistical requests and interpretations. Any protocol changes, as well as any adverse events, will also be reported to the Institutional Review Boards of all study sites, as well as to the NIH.

Privacy, Data Storage & Confidentiality

- Complete the Privacy and Confidentiality section of the iRIS submission form.

Describe Role of External Personnel:

N/A

Study Scope

Does this study have a cancer focus? Cancer focus includes studies that enroll >50% oncology or malignant hematology patients; or, preventing, detecting, and diagnosing cancer or understanding the impact of

cancer on patients and their caretakers.

○ Yes ◉ No

Are you using a drug, biologic, food, or dietary supplement in this study?

○ Yes ◉ No

Are you using a medical device, an algorithm (whether computer based or not), an in vitro diagnostic test, or using samples to look for biomarkers in this study?

○ Yes ◉ No

Does this study employ magnetic resonance, including imaging (MRI), spectroscopy (MRS), angiography (MRA) or elastography (MRE) beyond the standard of care?

○ Yes ◉ No

Does this study specify or require the performance of diagnostic procedures using ionizing radiation (x-rays, DEXA, CT scans, nuclear medicine scans, etc.) that are beyond the standard of care?

○ Yes ◉ No

Does this study specify or require the performance of therapeutic procedures using ionizing radiation (accelerator, brachytherapy or systemic radionuclide therapy) that are beyond the standard of care?

○ Yes ◉ No

Will the participant be subjected to increased or decreased ambient pressure?

○ Yes ◉ No

Do you plan to recruit subjects from Duke Regional Hospital (DRH)?

◉ Yes ○ No

Do you plan to recruit subjects from Duke Raleigh Hospital (DRAH)?

○ Yes ◉ No

Does this study utilize the Duke Early Phase Clinical Research Unit (DEPCRU)?

○ Yes ◉ No

Are you using the Duke logo in any advertisements?

○ Yes ◉ No

Is this study retrospective, prospective, or both?

"Retrospective" means that data or samples already in existence (collected prior to the study submission) will be used.
"Prospective" means there will be data or samples collected in this study for research purposes.

- ( ) Retrospective
- (•) Prospective
- ( ) Retrospective and Prospective

If the study is both retrospective and prospective: Is this a review soley of information collected for non-research purposes (i.e. a review of medical records)?

( ) Yes ( ) No

Does this protocol include any research using botulinum toxin, including the FDA-approved clinical product (Botox)?

( ) Yes (•) No

Does this protocol involve the administration of any of the following materials to humans?

•Any viral vector or plasmid
•Any cells that have been modified by a viral vector
•Any other genetically-modified cells
•Any genetically-modified virus, bacterium, or other agent
•Any other recombinant or synthetic nucleic acid

( ) Yes (•) No

Duke Regional Hospital (Site Specific)

Protocol calls for extra costs to subjects for:

Ambulatory Visits

( ) Yes (•) No

Hospitalization

( ) Yes (•) No

Drugs

( ) Yes (•) No

Tests

( ) Yes (•) No

Other

( ) Yes (•) No

Protocol calls for utilization of DRH Personnel/Resources:

Nursing personnel:

( ) Yes (•) No

Pharmacy:

( ) Yes (•) No

Lab:

○ Yes ◉ No

Radiology:

○ Yes ◉ No

Medical records:

○ Yes ◉ No

Resource distribution (IV pumps, etc):

○ Yes ◉ No

Operating room:

○ Yes ◉ No

Other:

○ Yes ◉ No

Protocol calls for Investigational items included in study:

Drugs:

○ Yes ◉ No

Device:

○ Yes ◉ No

Protocol calls for any confidential information:

Questionnaires:

◉ Yes ○ No

Records:

◉ Yes ○ No

Photo(s):

○ Yes ◉ No

Other:

○ Yes ◉ No

Protocol calls for any risks:

Psychological:

◉ Yes ○ No

Physical:

○ Yes ◉ No

Protocol calls for Subject Compensation?

(●) Yes ( ) No

How much?

All participants who consent and complete at least T1 will receive $20.00 for their time. For those participants who are enrolled into the RCT and remain eligible after T1, they will receive up to a total of $70.00 for the completion of all four surveys. For those participants who are enrolled into the ancillary study and remain eligible after T1, they will receive up to a total of $70.00 for the completion of all four surveys.

Compensation for participants is based on the completion of each study survey:

- T1: $20.00
- T2: $20.00
- T3 (mini survey): $10.00
- T4: $20.00

For individuals participating in Aim 4 of the RCT (or Aim 3 of the ancillary), they will receive $20.00 for the completion of the study visit.

Clinicians are not compensated for their study participation. This is to ensure and mitigate any potential research bias and/or coercion.

Compensation for participation is provided via ETR check request.

Will patients be:

(●) Inpatient
( ) Outpatient
( ) Both

Anticipated duration of study:

3 months

Study involves:

[x] Research data collection only
[ ] Drug(s)
[ ] Device
[ ] Therapy/treatment

Is this an NIH trial?

(●) Yes ( ) No

Grant #

1U54MED012530-01, 1R21NR016743-01A1

List anticipated services which are NOT standard of care (non-routine):

Service(s)
No services outside standard of care

Will DRH be paid by sponsor or other entity?

( ) Yes (•) No

Additional Questions

Should you have questions regarding this form, call Lynn Whitt, Duke Regional Hospital, at 470-4172 or Jody Power, DUHS IRB Office, at 668-2605. You may also email Lynn Whitt at lynn.whitt@duke.edu.

Should you have questions involving clinical issues or operational processes at DRH, please call:

Pharmacy:	Lynn Whitt	470-4172
Laboratory:	Ellen Poulson	613-5240
Radiology:	Robert Pagnanelli	470-5281
Nursing:	Gloria McNeil	470–7141
Surgical Services:	John Hudson	470-4730
Finance:	Shannon Baker	470-6543
Risk Management:	Ken Washington	470-8530

Subject Population Groups and Enrollment

Population Groups (Select targeted population groups only):

Note:

- If Minors are included, the study will be routed to the Department of Pediatrics for Pediatric Risk Assessment.
- Students and Employees over whom Key Personnel have a supervisory role may not be enrolled in this study

- [x] Adults
- [ ] Minors who are Wards of State
- [ ] Minors
- [x] Duke Patients
- [ ] Pregnant Women
- [ ] Fetuses
- [ ] Prisoners
- [x] Adults incapable of giving consent
- [ ] Adults with diminished capacity
- [ ] Handicapped subjects
- [ ] Students
- [ ] Employees
- [ ] Healthy Controls
- [ ] Deceased subjects
- [ ] Blanket Protocol

Please select any population groups excluded from participation in this study:

- [ ] Pregnant women

Maximum number of subjects to be consented at Duke:

Enter a single number. If you anticipate consenting a range of subjects, enter the **upper** limit of the range. The number should represent the maximum number of subjects for the life of the study.

550

Maximum number of subjects to be consented at all sites:

Enter a single number. If you anticipate consenting a range of subjects, enter the **upper** limit of the range. The number should represent the maximum number of subjects for the life of the study.

550

Subject Procedures and Costs

Biobank - Does this study involve the collection, use, tracking, banking (storage) or distribution of human biological specimens?

Human biological specimens include blood or its components, healthy or diseased tissue, bodily fluids, DNA /RNA or human stem cells.

○ Yes ◉ No

Procedures

Check all the apply:

- ☐ Genetic Testing
- ☐ Gene Transfer
- ☐ DNA Banking
- ☐ Testing for Reportable Infectious Diseases
- ☐ Human Cell Banking
- ☐ *Use of Human Embryonic Stem Cells
- ☐ *Use of Human-induced Pluripotent Stem Cells
- ☐ *Use of Other Cells Derived from Human Embryos
- ☐ *Use of Human/Animal Chimeric Cells
- ☐ *Specialized Cell Populations for Cell Therapy
- ☐ Use of Human Tissue
- ☐ Use of Bodily Fluids
- ☐ Use of Blood (or its components)
- ☑ Not Applicable

Will blood be drawn in this study for research purposes?

○ Yes ◉ No

Will the Operating Room be used in this study?

Include only research time, not clinical care time.

○ Yes ◉ No

Will there be extra costs to subjects or insurance as a result of the research (e.g. tests, hospitalization)?

○ Yes ◉ No

Will there be Subject Compensation?

(•) Yes ( ) No

Compensation for Travel / Lost Income (in USD):

0

Other Subject Compensation:

All participants who consent and complete at least T1 will receive $20.00 for their time. For those participants who are enrolled into the RCT and remain eligible after T1, they will receive up to a total of $70.00 for the completion of all four surveys. For those participants who are enrolled into the ancillary study and remain eligible after T1, they will receive up to a total of $70.00 for the completion of all four surveys.

Compensation for participants is based on the completion of each study survey:
T1: $20.00
T2: $20.00
T3 (mini survey): $10.00
T4: $20.00

Aim 4 participants will receive $20.00 compensation for their time for the single, audio recorded visit.

Clinicians are not compensated for their study participation. This is to ensure and mitigate any potential research bias and/or coercion.

Compensation will be provided via ETR check request.

Subject Recruitment Materials

For each document to be reviewed, use the table below to provide the following information:

Attach a copy of each advertisement that you will be using with this study in the Initial Submission Packet. If any Ad will have multiple wording variations, attach a copy of each version of the Ad.
All materials that will be used to advertise the study in order to recruit subjects must be approved by the IRB.

Types of subject recruitment materials include, but are not limited to, the following:

Direct Advertising
Posters
Billboards
Flyers
Brochures

Media Advertising
Newspaper Ads
Magazine Ads
Radio Ads
TV commericals / Video
Internet website
Social Media

Other Types of Advertising
Newsletter
Email
Postcards / Letters

(Note: Doctor-to-Doctor letters do not require IRB approval)

Document name	Material category	Location material displayed	Has this material previously been approved by the IRB?

ICUconnect informational video script and key images	( ) Billboard / Flyer / Poster<br>( ) Brochure<br>( ) Internet website / Email<br>( ) Letter / Postcard<br>( ) Phonescript<br>( ) Radio<br>(•) Television / Video<br>( ) Newsletter / Newspaper / Magazine<br>( ) Other	Please be specific. For example, "Duke" would not be an appropriate location. "Duke Hospital Television" would be an appropriate response.<br><br>This informational video will be shown to family members in the Duke ICUs identified as eligible for the protocol after we receive permission to discuss the study with them as an introduction to the study. This video may also be sent directly to eligible individuals as a link so that they can view the informational video on their personal devices at a later time.	(•) Yes ( ) No
Pro00090202 ICUconnect Usability Testing Recruitment Email	( ) Billboard / Flyer / Poster<br>( ) Brochure<br>(•) Internet website / Email<br>( ) Letter / Postcard<br>( ) Phonescript<br>( ) Radio<br>( ) Television / Video<br>( ) Newsletter / Newspaper / Magazine<br>( ) Other	Please be specific. For example, "Duke" would not be an appropriate location. "Duke Hospital Television" would be an appropriate response.<br><br>This email will be sent to community members who have previously agreed to be contacted for usability testing.	(•) Yes ( ) No
ICUconnect Family Flyer 4.5.19	(•) Billboard / Flyer / Poster<br>( ) Brochure<br>( ) Internet website / Email<br>( ) Letter / Postcard<br>( ) Phonescript<br>( ) Radio<br>( ) Television / Video<br>( ) Newsletter / Newspaper /	Please be specific. For example, "Duke" would not be an appropriate location. "Duke Hospital Television" would be an appropriate response.<br><br>This flyer will be posted in approved areas of Duke ICUs for the purpose of	(•) Yes ( ) No

	Magazine<br>○ Other	alerting family members to the presence of the study.	
REACH_Text_Script___v1.0_19 Apr 2021	○ Billboard / Flyer / Poster<br>○ Brochure<br>○ Internet website / Email<br>○ Letter / Postcard<br>○ Phonescript<br>○ Radio<br>○ Television / Video<br>○ Newsletter / Newspaper / Magazine<br>◉ Other	Please be specific. For example, "Duke" would not be an appropriate location. "Duke Hospital Television" would be an appropriate response.<br><br>This text message script will be sent to the legalized authorized representatives (LAR) for eligible subjects to help alert the LARs of the presence of the study.	○ Yes ◉ No

Consent Process

Attach draft consent forms in the Initial Review Submission Packet.

Consent forms must be MS Word documents and follow the specific format outlined by the IRB. **Click here** to download a copy of the consent form template.

Note: Please do not edit the section of the footer that contains the Protocol ID, Continuing Review and Reference Date fields. Those fields will be used to stamp the final consent form when it is approved by the IRB. If you want to add an internal version date, please put it in the header.

Who will conduct the consent process with prospective participants?

Give the person's role in this study (PI, Study Coordinator, etc.):

Delegated study team members, specifically Clinical Research Coordinators and/or Clinical Research Specialists Srs, will be allowed to consent for this study.

Who will provide consent or permission?

(Select all that apply):

- [x] Participant
- [ ] Parent(s) or Legal Guardian(s)
- [x] Legally Authorized Representative (LAR)

How much time will the prospective participant (or legally authorized representative) have between being approached about participating in the study and needing to decide whether or not to participate?

If you are not giving the person overnight to consider whether or not to participate, please justify.

The prospecitve participant will be given as much time as they need to consider participation within the research study within the constraints of study eligibility (i.e. participants are no longer eligible after 14 days of ICU admission).

Where will the consent process occur?

Consent will ideally occur in a quiet, private location such as the patient's ICU room or a designated private room behind a closed door. However, it is important to note that in many instances, eligible participants ask to meet in the waiting room/lobby of the ICU and/or ask for other members of their family and/or friends to be present during the informed consent. The study team will ensure that the location and individuals present at the time of consent are at the will and permission of the consenting individual.

If consent occurs remotely (via eConsent, over the telephone, and/or email), the delegated study team member performing the consent will ensure that they are in a quiet, private location where they can appropriately speak to the potential participant or send an email privately.

What steps will be taken in that location to protect the privacy of the prospective participant?

We will attempt to conduct the consent process in a private room during time periods when non-clinician traffic is low. We will also ask the prospective participant if they are comfortable discussing the study before beginning and if speaking by phone if they are in a private location.

How much time will be allocated for conducting the initial consent discussion, including presenting the information in the consent document and answering questions, with each prospective participant?

There is no time limit. We will allow as much time as needed by the prospective participant.

What arrangements will be in place for answering participant questions before and after the consent is signed?

We will answer all questions at the time of study consent. The prospective participant will be given phone numbers and/or pager numbers for the relevant clinical research coordinator and the site PI for additional questions.

Describe the steps taken to minimize the possibility of coercion or undue influence.

First, prior to consent occuring, the delegated study team member will obtain an appropriate research introduction from a member of the clinical team and/or other appropriate member as outlined by institutional policy. This will ensure that the prospective participant voluntarily chose to be approached by research and learn about the research opportunity for which they may qualify for. Second, if the recruitment and engagement policy is utilized, the study team member will ensure to ascertain that the potential participant is 1) willing to speak with them about the research study and 2) ascertain if the present is an appropriate time to talk about the research study prior to reviewing the informed consent with them. Third, all potential participants will be reminded that research is 100% voluntary and that there are no penalities for choosing not to participate in the study (or withdraw at a later time if they choose to consent). In addition the study team will specifically address that while the survey results are visible to the study team and assigned clinician (if intervention), that there will be no negative repercussions to them and that the clinician participating in the study and reviewing their needs will continue to treat them equitably.

What provisions will be in place to obtain consent from participants who do not read, are blind or who do not read/understand English?

We will exclude those who are blind and cannot read/understand English for two reasons: 1) our questionnaires are not validated in other languages and our web app has not been designed for other languages and 2) the app is not available for those individuals who are

blind and require brail.

Do you plan to obtain written consent for the conduct of research?

○ Yes ◉ No

Protected Health Information (PHI)

Indicate how you intend to use potential subjects' Protected Health Information (PHI):

○ I will review, but not record, PHI prior to consent.
◉ I will record PHI prior to consent.
○ I do not intend to use PHI prior to consent.
○ I will record PHI without consent. (decedent research, database repository, chart review)

Request for Waiver or Alteration of Consent and/or HIPAA Authorization

Will the population include deceased individuals?

○ Yes ◉ No

This waiver request applies to the following research activity or activities:

☐ Scheduling of research activities in MaestroCare and/or the recording of PHI via telephone for screening purposes prior to obtaining written consent for the research. Scheduling of research activities in MaestroCare and/or the recording of PHI via telephone for screening purposes prior to obtaining written consent for the research. (If you check this box, please complete all sections below.)

☑ Ascertainment (identification, selection) and/or recruitment of potential subjects while recording identifiable private information, such as protected health information (PHI), prior to obtaining the subject's consent. (If you check this box, please complete sections B and C below.)

☑ Conduct of the research project without obtaining verbal or written consent and authorization. (If you check this box, please complete sections B and C below.)

Note: Answer the questions below as they pertain solely to PHI collected prior to consent.

Provide the following information:

List the elements of informed consent and/or HIPAA authorization for which waiver or alteration is requested:

- Provide the rationale for each.

We would like to request a waiver for all elements of consent and HIPAA authorization with the intent to document and store this information for the purposes of determining clinician and patient/caregiver eligibility for study participation. We specifically intend to document and store this information, such as, but may not be limited to, name (first and last), contact information, relevant medical history pertaining to potential study eligibility (patient only), and MRN# (patient only). This information is necessary to collect as it will help us determine study eligibility and how to most easily contact a potential subject for a research consent visit. The information we are requesting to collect is minimal in nature and necessary to conduct the research study.

List the specific protected health information (PHI) to be collected and its source(s):

- (Note: PHI = health information + identifiers)

As noted above, we are requesting a waiver for all elements of consent and HIPAA authorization. The specific PHI that may be collected varies based on subject type. If the potential subject is a clinician, we

would like to collect, at minimum, first and last name and contact information prior to consent. This information will be obtained from the Duke internal directory and stored in a Duke DHTS maintained database known as Study Tracking, which was created and is actively secured under a Duke server for Duke clinical research teams within the REACH equity project. It is necessary to obtain and document this information as it creates a profile which feeds into the study REDCap database and ultimately enables the study team to electronically send an eConsent to the clinician if they are agreeable to review the informed consent for study participation. If the clinician declines consent, their information will remain in Study Tracking and the status of decline will be automatically added. It is important to maintain a list of clinicians who have declined as the study team desires to mitigate research-related burden by not re-approaching for potential consent. If the potential subject is a patient and their caregiver, the PHI that may be documented and stored prior to consent is name of patient, MRN# and relevant health information related to the study's inclusion/exclusion criteria, at a minimum. This information will be obtained from the patient' s EMR and stored on a secure excel file on the Duke server, as well as Study Tracking, and only accessed by study key personnel. This information is necessary for screening purposes as it will help identify eligible subjects. Given the patient population under study (admitted, ventilated patients), the caregiver will sign informed consent on their behalf (LAR) and will also be approached to participate in the study. Should a patient not participate due to ineligibility or a lack of desire to participate in the study (at the caregiver's discretion), the study team would like to request the permission to retain the limited PHI data (name, MRN#, date screened and/or approached) and document the reason for ineligibility or decline to participate and store this information in the electronic database known as Study Tracking. The reason for this is to 1) ensure that the same patient and caregiver are not re-approached during their hospital admission, as admissions can continue on for a significant amount of time, 2) to inform the study team regarding study design as it pertains to eligibility, and 3) to ensure that researchers are not systematically excluding people for reasons that seem unfair/incorrect that were not clear initially.

Criteria for Waiver: The DUHS IRB may waive the requirement for informed consent and authorization if all of the following criteria are met:

- Please respond to each item in the space below using protocol-specific language to provide justification:

a) The research or clinical investigation involves no more than minimal risk to subjects:

This study tests the efficacy of the ICUconnect web app for communicating family needs to ICU clinicians vs. the standard of care for family members of patients who are on mechanical ventilation for at least 24 hours. There are no physical risks for subjects. There is a risk of mild anxiety when answering study questions as well as the potential (and standard) risks of loss of confidentiality. We feel that these risks are minimal.

b) The waiver or alteration will not adversely affect the rights and welfare of the subjects. Include a description of any measures to be taken to ensure that the rights and welfare of subjects will be protected:

This is a minimal risk project. We have a tested plan to use the minimum data required and all data collected will be stored securely as described in the approved RDSP. We have great respect for the process and have put much thought into designing a reasonable system to uphold subjects' rights, safety and well-being.

c) Whenever appropriate, the subjects will be provided with additional pertinent information after participation:

Yes, when applicable the subjects will be provided with additional pertinent information after study participation has occurred.

d) If this research activity relates to research involving deception, explain how subjects will be provided with additional pertinent information after study participation and what information will be provided. Otherwise indicate "not applicable":

Not applicable

e) The use or disclosure of protected health information involves no more than minimal risk to the privacy of individuals, based on, at least, the presence of the following elements (e1. and e2.)

Demonstrate that the use or disclosure of PHI involves no more than minimal risk to the privacy of subjects by describing the plans requested below:

e1) An adequate plan to protect the identifiers from improper use and disclosure.

Describe the plan (how protection will be accomplished) and indicate where the PHI will be stored and who will have access:

Identifiers recorded for the purposes of screening and enrollment will be stored on an excel spreadsheet and stored in a limited access, password protected folder on a secure Duke server behind the Duke firewall. In addition, the identifiers described above will be imported into the Duke DHTS database, known as Study Tracking, created by a Duke database analyst for the purpose of creating subject IDs and tracking subject data as it relates to enrollment and reasons for exclusion that will be used to help researchers better identify and understand trends relating to why participants are ineligible. Identifiers will be also imported into REDCap for the purposes of sending an eConsent to a potential clinican particiapnt via email. The servers being utilized are all protected behind the Duke firewall and will not be used for any other purpose other than research conducted under the scope of this IRB application.

e2) An adequate plan to destroy the identifiers at the earliest opportunity consistent with conduct of the research, unless there is a health or research justification for retaining the identifiers or such retention is otherwise required by law.

Describe the plan (how and when identifiers will be destroyed and by whom). If there is a health or research justification for retaining the identifiers or such retention is otherwise required by law, provide the reason to retain identifiers:

This information will be collected and maintained for the duration of the project to minimize research-related burden on participants so as to ensure that re-approach does not occur should a participant not qualify or decline approach/consent. The data obtained regarding ineligibility may be used to redefine future study design regarding inclusion/exclusion criteria, as well as facilitate in assessing the current study inclusion/exclusion criteria. Additionally, the data will be used to ensure that researchers are not systematically excluding people for reasons that seem unfair/incorrect that were not clear initially.No additional data will be collected after refusal of informed consent is given.

e3) Adequate written assurances that the protected health information will not be reused or disclosed to any other person or entity except (i) as required by law, (ii) for authorized oversight of the research study, or (iii) for other research for which the use or disclosure of PHI would be permitted by the HIPAA Privacy Rule. By electronically signing this submission, the PI provides this written assurance:

By electronically signing this submission, the PI provides this written assurance.

f) The research could not practicably be conducted or carried out without the waiver or alteration:

- Explain why informed consent/authorization can not be obtained from subjects.

We otherwise could not identify participants for this unique research given the chaotic nature of intensive care units and the difficulty of relying solely on voluntary self-identification (or identification by ICU physicians).

g) The research could not practicably be conducted or carried out without access to and use of the protected health information:

For the clinicians, it is important to collect and document specific PHI, such as name and contact information, to send him/her the eConsent from REDCap. The REDCap project is designed to automatically take the clinicians through the first survey, if they choose to consent, without direct coordinator involvement. This minimizes the research burden felt by clinician who chooses to participate given their intense ICU case load to manage. For patients and their caregivers, without the ability to record the described PHI, it would be impossible to determine eligibility. Further, we would have no idea how to contact the patient's legal representative. In addition, given the patient population under study, we want to ensure that research burden is minimized. Therefore, if they do not qualify or they choose to decline, we ask to maintain electronically stored documentation to avoid re-approach.

h) For research using biospecimens or identifiable information, the research could not practicably be carried out without access to and use of the protected health information:

N/A, not applicable. This research does not utilize biospecimens; however, it does use identifiable information such as name, contact information and/or MRN#. As stated above, we are requesting this waiver for all elements of consent and HIPAA authorization. These elements of PHI are necessary to determine eligibility and mitigate research-related burden for the clinicians, the patients and their caregivers.

Privacy and Confidentiality

Explain how you will ensure that the subject's privacy will be protected:

Consider privacy interests regarding time and place where subjects provide information, the nature of the information they provide, and the type of experience they will be asked to participate in during the research.

We will closely guard privacy and are very sensitive to this concern. All participants (caregivers /LARs) will be interviewed in either a private room in the hospital or by telephone. Clinicians will be apprached by an investigator to provide an introduction to the study and then asked if it would be okay to receive an email with the eConsent link. All interviews will be performed when convenient for the participant, and at times they feel comfortable--that is, when their stress level is not reported to be high. They (caregivers/LARs) will provide information on their distress and memories of the ICU, which could be sensitive subjects for participants. However, study staff will draw on their experience administering similar questionnaires among similar participants to set them at ease. We are confident that we can respectfully and safely perform this study as well as respect privacy to the highest degree possible in a research setting.

Describe how research data will be stored and secured to ensure confidentiality:

How will the research records and data be protected against inappropriate use or disclosure, or malicious or accidental loss or destruction? Records and data include, for example, informed consent documents, case report forms or study flow sheets, survey instruments, database or spreadsheets, screening logs or telephone eligibility sheets, web based information gathering tools, audio/video/photo recordings of subjects, labeled specimens, data about subjects, and subject identifiers such as social security number.

All electronic data will be stored in a Duke DHTS hosted, secured, and maintained database, located on a secured Duke PHI server, known as Study Tracking, as well as in a Duke secured REDCap database located on Duke secured servers. Both databases and servers are maintained by Duke DHTS, and are backed up on mirrored servers routinely.

The sociodemographic and questionnaire data will be collected from study participants via an online survey portal integrated into our ICUconnect web app, which will flow in a one-way submission direction to our study backend system via a proxy server. This portal will be accessed by participants by a one-time unique URL sent to them via email or text (similar to how REDCap sends survey invites out). No data will go back out to the HTTP participant portal. In the event that the app is not available, surveys will be printed out and administered to the study participants either in person or via phone.

All clinical data will be entered directly by CRCs either by password protected, encrypted tablet computers or Duke desktop computers into our Duke REDCap database. Similarly, CRC's will enter all data collected via telephone during follow-up interviews directly into the REDCap database. Standard operating procedures for data management at Duke emphasize security procedures for maintaining patient confidentiality. All study staff will adhere to these procedures in this study.

Daily screening logs and enrollment logs will be completed on excel spreadsheets and kept on a limited access shared Duke server and/or REDCap.

All consent forms and any paper data collection will be kept in a locked cabinet in either the PIs or the study coordinator's locked office or within the secure drive and/or secure REDCap. All SSN's collected for payment purposes will be kept in the study coordinator's locked office in a locked cabinet or within the secure drive. Only the study coordinators will have access to these documents for payment processing. SSN's will be redacted as soon as the subject payment has been processed.

Request for Waiver of Documentation of Informed Consent

Use this form to request DUHS IRB review if you propose to obtain informed consent for the research activity without also obtaining the subject's signature on the consent form. Note: If the IRB grants this waiver, the investigator will still be required to provide information about the research to each potential subject, but the subject's signature on the form will not be required. A written script of the information that will be read or given to potential subjects must be provided

for IRB review with this submission. In addition to describing the study, the script must contain the basic elements of informed consent, as referenced in 45CFR46.116(a) and 21CFR50.25. If the study will collect protected health information (PHI), the script must also present the core elements of authorization, as referenced in 45CFR164.508(c). A request for waiver or alteration of HIPAA authorization, found on the IRB website (http://irb.duhs.duke.edu/), must also be submitted.

This type of waiver is useful for some telephone or internet surveys, questionnaires, or when signing the consent document could have a negative consequence for the subject.

Conditions for Waiver: In accordance with federal regulations, the DUHS IRB may waive the requirement for the principal investigator to obtain a signed consent form for some or all subjects if it finds that one of two conditions are met:

- [ ] The only record linking the subject and the research would be the consent document and the principal risk would be potential harm resulting from a breach of confidentiality. Each subject will be asked whether the subject wants documentation linking the subject with the research; and the subject's wishes will govern. [45 CFR 46.117(c)(1)]

This applies only to research that is subject to Department of Health and Human Services (HHS) regulations. It does not apply to research that is subject to Food and Drug Administration (FDA) regulations.

Or

- [x] The research presents no more than minimal risk of harm to subjects and involves no procedures for which written consent is normally required outside of the research context. [45 CFR 46.117(c)(2); 21CFR56.109(c)(1)]

This applies to research that is subject to either HHS regulations or FDA regulations.

In cases in which the documentation requirement is waived under these conditions, the IRB may require the investigator to provide subjects with a written statement regarding the research. [**21CFR56.109(d)**]

- **Note:** FDA regulation **21CFR50.24** provides an additional exception from informed consent requirements for emergency research. Briefly, to qualify under this regulation the research would involve human subjects in a life-threatening situation, for which available treatments are unproven or unsatisfactory, and for which the collection of valid scientific evidence is necessary to determine the safety and effectiveness of particular interventions. Obtaining informed consent will not be feasible, participation in the research holds out the prospect of direct benefit to the subjects and the clinical investigation could not practicably be carried out without the waiver.

  This information is presented for the sake of completeness; however, **waivers of documentation of informed consent under 21CFR50.24 are not applicable to this request form and will not be considered further.**

Waiver Qualification

Please provide a response to each statement below and continue as instructed:

The only record linking the subject and the research would be the consent document:

○ True
◉ False

The research does not qualify for a waiver of documentation of informed consent under this condition.

The research presents no more than minimal risk* of harm to subjects:

- *Minimal risk: The probability and magnitude of physical or psychological harm or discomfort anticipated in the research are not greater in and of themselves than those ordinarily encountered in daily life or during the performance of routine physical or psychological examinations or tests.

(●) True
( ) False

The research involves no procedures for which written consent is normally required outside of the research context:

(●) True
( ) False

Supporting Information

Please answer the following questions and provide information and materials as instructed.

What is the rationale for this request for waiver of documentation of informed consent?

- Provide sufficient detail to demonstrate that the research meets one of the two conditions listed above.

This is a minimal risk study involving no medical procedures for research purposes. Occasionally caregivers /legal representatives cannot meet or are known to have other barriers preventing travel to the study ICU within 24-48 hours. If an in-person meeting is impossible per their schedule and they are not able to use the REDCap eConsent form alternative, we will obtain verbal consent by reading a telephone consent script. We will still mail two copies of the consent form with an explanatory cover letter to them should telephone consent occur. They will be instructed to sign each (with a witness documenting signature as well) and return one copy in an enclosed stamped envelope to the study PI. We are requesting this waiver should the participant complete all study procedures by phone or online and neglect to return the mailed consent form and should we be unable to contact him/her after Interview 2.

All data forms, electronic or printed, will be labeled with a study ID number linked to a master list of names and medical record numbers kept on an excel spreadsheet limited access Duke server. The master list of study ID linkage to this personal data will be destroyed after study completion. Any other materials, electronic or printed, will be de-identified prior to analysis. As a result, the principal risk associated with the link between the subject and the consent form would be a breach of confidentiality. While all consents will be kept in a locked filing cabinet in the study coordinator's locked office, there is still a theoretical risk of loss of confidentiality of data given known limitations of data systems and human inputs.

What materials will be given or read to potential subjects to inform them about the study?

- All materials must be submitted for IRB review, must accompany this waiver request (e.g. an information sheet, consent form without signature lines, or script of information for verbal interaction with a potential subject) and must include the required elements of informed consent and authorization in accordance with the regulations cited above. Attachments can be included in the Initial Review Submission Packet.

CRC’s will read a telephone script that provides all the necessary information about the study for the subject to provide informed consent. Two copies of the consent form will then be mailed to the caregiver /legal representatives. They will be instructed to sign each (with a witness documenting signature as well) and return one copy in an enclosed stamped envelope to the study PI.

Does the written material include the requirement for the signature of the subject or his/her legally authorized representative?

[x] Yes
[ ] No
[ ] N/A

If the written description or script is to be signed by the subject or his/her legally authorized representative, and the consent process occurs by telephone, does the written description or script include the requirement for signature by a witness to confirm the identity of the subject?

[x] Yes

☐ No
☐ N/A

Application Questions Complete

Please click Save & Continue to proceed to the Initial Submission Packet.

The Initial Submission Packet is a short form filled out after the protocol application has been completed. This is an area to attach protocol-related documents, consent forms, and review the application.

Statistical Analysis Plan for ICU Connect

The goal of the primary hypothesis was to determine the efficacy of the ICUconnect intervention versus usual care on reduction in unmet needs from T1 to T2. Unmet needs were assessed at T1, T2, and T3 with the total NEST score, a continuous, normally distributed measure. Because each family member was surveyed at multiple time points and the same ICU physician saw several patients, outcome variable measurements were not be independent. Therefore hierarchical linear models fit via PROC MIXED in SAS 9.4 (Cary, NC) were used as the primary analytic strategy they appropriately accounted for the multiple types of correlation inherent in our study design. The other continuous, longitudinal outcomes included: PHQ-9, GAD-7, PTSS, and QOC; hierarchical linear models were also used to estimate the treatment effect over time for these outcomes. The binary outcomes of goal concordant care and IPC (dichotomized due to ceiling effects) were analyzed via generalized linear models with a logit link and fit with generalized estimating equations (PROC GENMOD in SAS, 9.4). Finally, treatment group differences in hospital and ICU length of stay were examined with a Wilcoxon test.
