# Supplementary material for: Mobile Application–Based Communication Facilitation Platform for Family Members of Critically Ill Patients: A Randomized Clinical Trial
Source: JAMA Netw Open. 2024 Jan 4;7(1):e2349666. doi: 10.1001/jamanetworkopen.2023.49666 (PMC10767607; doi:10.1001/jamanetworkopen.2023.49666)
Supplement: Supplement 2. — eAppendix 1. Eligibility Criteria eFigure 1. Study Flow eFigure 2. Mobile App Notification Strategy eAppendix 2. List of Questions Provided to Intervention Family Members eFigure 3. Screenshots From ICUconnect App eAppendix 3. Training of Family Members and Physicians eAppendix 4. Family Meeting Conduct eAppendix 5. Procedure for Managing Changes in ICU Attendings Occurring During Intervention Time Period for Family Members eAppendix 6. NEST Items eAppendix 7. SAS Code for Primary Outcomes eTable 1. Characteristics of ICU Attending Physicians eTable 2. Family Member and Patient Characteristics by Race eTable 3. Clinical Characteristics and Clinical Outcomes eTable 4. Consent and Retention by Race and Group eFigure 4. Primary Outcomes at Day 3 by Treatment Group and Physician eFigure 5. Graph of Baseline NEST Scores by Month of Study eFigure 6. Primary Outcomes at Day 3 by Treatment Group, Race, and Physician eFigure 7. NEST Item Changes Over Time eTable 5. Needs Reported by Family Members in Their Own Words eTable 6. Outcomes by Race and Study Group [file jamanetwopen-e2349666-s002.pdf]

Supplemental Online Content

Cox CE, Ashana DC, Riley IL, et al. Mobile Application–based communication facilitation platform for family members of critically ill patients. JAMA Netw Open. 2024;7(1):e2349666. doi:10.1001/jamanetworkopen.2023.49666

eAppendix 1. Eligibility Criteria
eFigure 1. Study Flow
eFigure 2. Mobile App Notification Strategy
eAppendix 2. List of Questions Provided to Intervention Family Members
eFigure 3. Screenshots From ICUconnect App
eAppendix 3. Training of Family Members and Physicians
eAppendix 4. Family Meeting Conduct
eAppendix 5. Procedure for Managing Changes in ICU Attendings Occurring During Intervention Time Period for Family Members
eAppendix 6. NEST Items
eAppendix 7. SAS Code for Primary Outcomes
eTable 1. Characteristics of ICU Attending Physicians
eTable 2. Family Member and Patient Characteristics by Race
eTable 3. Clinical Characteristics and Clinical Outcomes
eTable 4. Consent and Retention by Race and Group
eFigure 4. Primary Outcomes at Day 3 by Treatment Group and Physician
eFigure 5. Graph of Baseline NEST Scores by Month of Study
eFigure 6. Primary Outcomes at Day 3 by Treatment Group, Race, and Physician
eFigure 7. NEST Item Changes Over Time
eTable 5. Needs Reported by Family Members in Their Own Words
eTable 6. Outcomes by Race

This supplemental material has been provided by the authors to give readers additional information about their work.

© 2024 Cox CE et al. *JAMA Network Open.*

eAppendix 1. Eligibility Criteria
Note that the procedure for screening began with the race recorded for patients in the electronic health record system. That is, if a patient's race (generally self-reported) was either White of Black, study staff would approach the family member.

Patients
Inclusion Criteria:
- ≥18 years of age
- Receive mechanical ventilation in a study ICU for ≥48 hours under care of a study ICU physician

Exclusion Criteria (pre-consent):
- Decisional capacity
- Death expected within 24 hours
- Admission to an ICU at the index hospital >14 days
- Care in ICU >7 days
- Comfort care or withdrawal of treatment planned
- Imprisoned
- Extubated and possess decisional capacity prior to informed consent
- Died before day 3 survey survey complete
- No known family or surrogate
- Care assumed by a non-study ICU attending after consent by patient/family but before family member completion of baseline survey
- Care assumed by non-study ICU attending <3 days after family member completion of baseline survey but before day 3 survey
- Study ICU attending physicians from different groups (i.e., intervention or control) caring for patient change <3 days after baseline survey completed by family

Exclusion Criteria (post-consent):
- Patient regains decision making capacity before day 3 survey
- Patient dies before day 3 survey

Family members
Inclusion Criteria:
- ≥18 years of age
- Self-described as the individual (related or unrelated) who provides the most support and with whom the patient has a significant relationship (per definition of 'family' described in the Society of Critical Care Medicine 2016 Guidelines for Family-Centered Care in the Neonatal, Pediatric, and Adult ICU)

Exclusion Criteria (pre-consent):
- Lack a knowledge of English such that the potential participant is not confident that they could complete study tasks (app viewing, surveys)
- Imprisoned
- Unable to complete surveys for any reason
- Describe their race as neither White nor Black
- Describe their ethnicity as Hispanic

Exclusion criteria (post-consent):

© 2024 Cox CE et al. *JAMA Network Open.*

- If the assigned randomized ICU attending physician either leaves ICU service or is replaced by a non-participating ICU physician ≤2 calendar days after family member completes T1 survey
- Low need burden (NEST score <15)

ICU physicians

Inclusion Criteria:

- ≥18 years of age
- Attending physician in a study ICU

Exclusion Criteria:

- None

*The NEST score cutoff was changed on June 26, 2019. At this point, 3 patients had been randomized. This change was based on our review of data (NEST + psychological distress symptoms) from a parallel cohort study of >50 family members of ICU patients who met nearly identical eligibility criteria as for the ICUconnect trial. This was a larger cohort than the pilot study on which we based the NEST cutoff.

© 2024 Cox CE et al. *JAMA Network Open.*

eAppendix 3. Training of Family Members and Physicians

The intervention was designed as an in-the-moment communication facilitation platform that operated using a shared language of needs that could be understood easily by both family members and physicians. While it offered some static reference features that could be accessed anytime, its main purpose was to send text or email messages (as the user preferred) that prompted an action intended to result in reducing unmet needs (e.g., report needs, conduct family meeting, etc).

Because the system was so straightforward and simple, intensive training was not required.

- <u>Family members</u> received training at the time of consent from the clinical research coordinator who used materials such as videos, printed handouts describing tasks and timelines and help resources, and a brief demonstration using a fictional family member account displayed on a tablet computer.
- <u>ICU physicians</u> received general exposure to the intervention, trial design, timelines, and expectations through a series of presentations the study team made during staff meetings for each study ICU. At the time of consent, the clinical research coordinator performed a similar series of steps as with family members described above. For physicians, staff let them navigate in a demo physician environment to see app capabilities. The physician was sent several text messages from the app platform for demo patients while the coordinator watched their responses to texts sent to prompt different tasks. Physicians were given a printed card with timeline information (see example of one page of this card), the study website that included a synopsis training video, and help information. Coordinators would give quick refresher updates to physicians who had gone over a month without a trial participant enrolled under them.

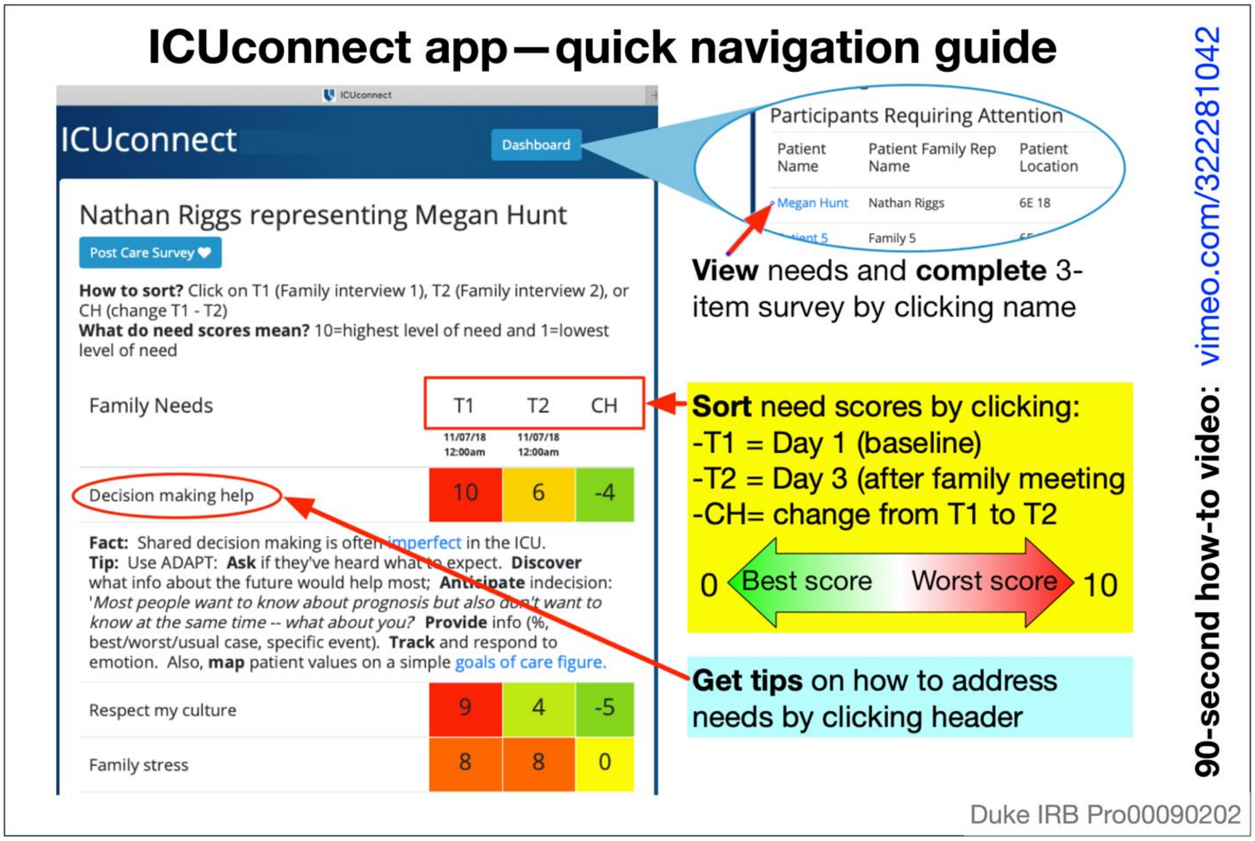

© 2024 Cox CE et al. *JAMA Network Open.*

eAppendix 4. Family Meeting Conduct

The goals of the two family meetings were for the ICU attending physician (and ICU team if possible) to substantively discuss with the family member at least the 3-4 most serious needs reported via the ICUconnect mobile app and using the tips included within the app, to explore patient values and treatment goals, and to address questions. No other guidance was provided; physicians were encouraged to use the style and language with which they felt most comfortable.

We asked the ICU physicians to conduct the meetings in person and in a private room whenever possible. However, during 2020-2022 the COVID-19 pandemic forced the meetings to be conducted primarily by telephone or by teleconference. Study staff did not schedule family meetings.

© 2024 Cox CE et al. *JAMA Network Open.*

eAppendix 5. Procedure for Managing Changes in ICU Attendings Occurring During Intervention Time Period for Family Members

Timing of MD Transition	Attending Change Scenario: MD 1 – MD 2	Included/Excluded	MD Bucket
**After Consent but Prior to T1**	Control-control	Included	MD2
	Control-intervention	Included	MD2
	Intervention-Intervention	Included	MD2
	Intervention-Control	Included	MD2
**After T1 but Before T2**<br><br>*It is assumed that if MD change occurs ≥3 days after T1 that intervention was received	Control-control	Included	MD1
	Control-intervention	Included	<3 days-MD1- stay under control MD/no reassignment
			>3 days- MD1
	Intervention-Intervention	Included	MD2 **if** < 3 days after T1<br>*CRC reassign in app and communicate to MD2 to view T1 and have family meeting
	Intervention-Control	Excluded/PI Withdraw?	CRC to avoid this
**After T2**	Control-control	Included	MD1
	Control-intervention	Included	MD1
	Intervention-Intervention	Included	MD1
	Intervention-Control	Included	MD1

© 2024 Cox CE et al. *JAMA Network Open.*

eAppendix 7. SAS Code for Primary Outcomes

```
proc mixed data=working;
	class clinician_id record_id timept ;
	model nest=t2  t2*rand_dumm t3 t3*rand_dumm prac_yr_ctr female_ctr surg_ctr /
		ddfm=kr solution  cl;
	repeated timept / subject=record_id type = cs rcorr;
	random intercept / subject=clinician_id type=vc;
where _timept in (0,1,2);

   ESTIMATE '0A: T1, CONTROL' INTERCEPT 1 T2 0 T3 0 t2*rand_dumm 0 t3*rand_dumm 0 /
CL;
  ESTIMATE '1A: T2, CONTROL' INTERCEPT 1 T2 1 T3 0 t2*rand_dumm 0 t3*rand_dumm 0/
CL;
  ESTIMATE '2A: T3, CONTROL' INTERCEPT 1 T2 0 T3 1 t2*rand_dumm 0 t3*rand_dumm 0/
CL;

  ESTIMATE '0B: T1, INT' INTERCEPT 1 T2 0 T3 0 t2*rand_dumm 0 t3*rand_dumm 0/ CL;
  ESTIMATE '1B: T2, INT' INTERCEPT 1 T2 1 T3 0 t2*rand_dumm 1 t3*rand_dumm 0/ CL;
  ESTIMATE '2B: T3, INT' INTERCEPT 1 T2 0 T3 1 t2*rand_dumm 0 t3*rand_dumm 1/ CL;

  ODS OUTPUT ESTIMATES=ESTIMATES_nest;
RUN;
```

© 2024 Cox CE et al. *JAMA Network Open.*

eAppendix 2. List of Questions Provided to Intervention Family Members

Here are three groups of questions that you can use right now to get information and support.

First, there are examples of real questions for people to ask **ICU teams**. They are grouped under specific topics to make them easier to use. Many people have told us that these questions were really helpful.

Second, we list questions that are important to ask yourself about **your loved one** who is a patient in the ICU.

Third, we list questions to ask yourself about how **you** are coping with things.

1. Questions that you can ask the ICU team

Information

The medical problem (that is, the diagnosis)
- What is the main medical problem right now?
- Is the medical problem affecting many body organs
- Is my loved one on life support?

Treatments
- Who are the different people taking care of my loved one?
- What is the main treatment my loved one is getting?
- How long does it take in general to see a response from this treatment?
- Are there any other treatments that would help?

Procedures
- What is the purpose of the procedure?
- How likely is the procedure to work?
- What are the main risks?
- Are their alternatives to doing this procedure?

Decisions

General questions
- What are the main decisions I need to be thinking about?
- What do other people in my position often decide to do?
- What is your advice about what I should do?

Being a substitute decision maker for a loved one
- How can I be a good substitute decision maker for my loved one?
- What if my loved one never talked to me about his/her beliefs about life support?
- Do I need a copy of my loved one's Advance Directives to make decisions?

Uncertainty
- I don't know what to do—or how to decide. Can you help me think through things?
- How sure are you about what to expect?
- Sometimes a doctor says things are better and then later a different doctor says that things are about the same. How can this be?

What should I expect?

What to expect short-term?
- How long do you expect my loved one to be in the ICU?
- How long do you think it will take to see signs of improvement?
- What are you looking for as a sign of improvement? Or a sign of worsening?

- Is there an important milestone you are looking for?

What to expect long-term?

- What does the recovery period look like in general?
- How do you think this illness will affect my loved one's future quality of life?
- How likely is it that my loved one will regain their baseline health?
- How likely is it that my loved one will regain their ability to be independent?
- How likely is it that this illness will affect my loved one's thinking and cognitive function?

Asking about prognosis

- I want to know what to expect. Could you tell me about prognosis in a couple different ways so that we understand better:
    - What are the statistics? Like, out of 100 people like my loved one, how many will get better and how many won't?
    - What are the best case and worse case scenarios?
    - What do you think is the most likely outcome?

Conflict and disagreement

- Within the family: I am worried about conflict within our family, but I don't know what to do. Can you give me advice on how to handle it?
- Between the ICU team and the family: I know we all want the same thing--to do right by my loved one. But I'm worried that there is disagreement about what to do between our family and the medical team. Can I tell you what is on my mind?
- Between the ICU team and the family: I am worried that there is a lot of disagreement between our family and the medical team. Can we get someone to help us both work through this situation?

Feeling stressed out or worried

- I am having a hard time with this situation. Is there someone I could talk to about my stress and worry?
- How do other people manage their stress and sadness in situations like this?

Culture, language, faith / religion / spirituality

- Could I get help with a translator for myself / my family?
- We have [cultural / religious / spiritual] beliefs that are important to how we think about medical care and decisions. Could I tell you more about this?

2. Questions about your loved one that you should think about

What my loved one values

- What are the things in life that are most important to my loved one?
- Is the treatment my loved one getting likely to result in a quality of life they would accept?
- To increase the chance of survival, would your loved one accept being dependent on others? Or have difficulty thinking as a result of ICU care?
- Am I making decisions for my loved one as if I were standing in their shoes? Or am I making decisions based on what I want for my loved one?

Symptoms

- Does my loved one look comfortable—or do they seem to have pain, shortness of breath, anxiety, or confusion?

© 2024 Cox CE et al. *JAMA Network Open.*

3. Questions about yourself (remember, we can help)
**Support**
• Do I need help or support from others—or just a person to talk to?
**Faith and spirituality**
• Would it help to talk to a chaplain or a spiritual leader?
**Finances**
• Am I worried about money and finances?

© 2024 Cox CE et al. *JAMA Network Open.*

eAppendix 6. NEST Items

		Not at all Very much so
1	I have regular talks with the ICU doctors about the medical condition, treatments, and what to expect<br>**Short title: Communication**	0 1 2 3 4 5 6 7 8 9 10
2	I always have someone to give me support<br>**Short title: Social support**	0 1 2 3 4 5 6 7 8 9 10
3	My loved one is completely comfortable (*has no pain, shortness of breath, anxiety, or confusion*)<br>**Short title: Patient symptoms**	0 1 2 3 4 5 6 7 8 9 10
4	I feel calm and in control in the ICU<br>**Short title: Family stress**	0 1 2 3 4 5 6 7 8 9 10
5	The ICU doctors understand my spiritual beliefs<br>**Short title: Spiritual**	0 1 2 3 4 5 6 7 8 9 10
6	I participate as much as I want in medical decisions in the ICU<br>**Short title: Decision making**	0 1 2 3 4 5 6 7 8 9 10
7	The financial impact of my loved one's illness is easy for our family to handle<br>**Short title: Financial stress**	0 1 2 3 4 5 6 7 8 9 10
8	The ICU doctors give me medical information in a way that is easy to understand<br>**Short title: Information**	0 1 2 3 4 5 6 7 8 9 10
9	The ICU doctors explained the possible long-term effects of my loved one's illness<br>**Short title: Long-term outcome**	0 1 2 3 4 5 6 7 8 9 10
10	The current ICU treatment plan fits well with my loved one's values<br>**Short title: Treatment=values**	0 1 2 3 4 5 6 7 8 9 10
11	I trust the medical information I get from the ICU doctors<br>**Short title: Trust**	0 1 2 3 4 5 6 7 8 9 10
12	The ICU doctors respect the things that are important to me (*for example: culture, values, language*)<br>**Short title: Respect culture**	0 1 2 3 4 5 6 7 8 9 10
13	The ICU doctors take the time listen to what I say and to answer my questions<br>**Short title: Listen & answer Qs**	0 1 2 3 4 5 6 7 8 9 10
14	Write any other need or concern here:<br>______________________________	*Rate how important this is to you*:<br>0 1 2 3 4 5 6 7 8 9 10

© 2024 Cox CE et al. *JAMA Network Open.*

eTable 1. Characteristics of ICU Attending Physicians

Characteristic	Total<br>n=37	Intervention<br>n=19	Control<br>n=18	*p*
**Participants enrolled within each physician's cluster**				
Total participants, median (IQR)	2.00 (1.00, 4.00)	3.00 (1.00, 3.00)	2.00 (1.00, 5.00)	0.888
White participants, median (IQR)	1.00 (0.00, 2.00)	1.00 (0.00, 2.00)	1.50 (1.00, 2.00)	0.356
Black participants, median (IQR)	1.00 (1.00, 2.00)	1.00 (1.00, 2.00)	1.00 (0.00, 1.75)	0.202
**Gender at Birth***				0.737
Male	23 (62%)	12 (63%)	11 (61%)	
Female	14 (38%)	7 (37%)	7 (39%)	
**Gender described**				0.737
Man	23 (62%)	12 (63%)	11 (61%)	
Woman	14 (38%)	7 (37%)	7 (39%)	
**Race**				0.490
White	31 (84%)	15 (79%)	16 (89%)	
Asian	5 (14%)	3 (16%)	2 (11%)	
Another race	1 (3%)	1 (5%)	0	
**Ethnicity**				1.000
Not Hispanic or Latino	34 (92%)	18 (95%)	16 (89%)	
Hispanic or Latino	3 (8%)	1 (5%)	2 (11%)	
**Years of practice***				
<1 year	4 (11%)	2 (11%)	2 (11%)	0.682
1-5 years	10 (27%)	4 (21%)	6 (33%)	
6-9 years	6 (16%)	4 (21%)	2 (11%)	
10-15 years	8 (22%)	3 (16%)	5 (28%)	
>15 years	9 (24%)	6 (32%)	3 (17%)	
**Type of practice***				0.737
Medical (medical, cardiac ICUs)	23 (62%)	12 (63%)	11 (61%)	
Surgical ((surgical ICUs)	14 (38%)	7 (37%)	7 (39%)	
**Practice specialty**				0.342
Medicine	23 (62%)	12 (63%)	11 (61%)	
Surgery	3 (8%)	1 (5%)	2 (11%)	
Anesthesia	11 (30%)	6 (32%)	5 (28%)	
**Weeks attending in an ICU**				0.068
≤4 weeks	0	0	0	
5-8 weeks	20 (54%)	8 (42%)	12 (67%)	
9-16 weeks	16 (43%)	11 (58%)	5 (28%)	
>16 weeks	1 (3%)	0	1 (6%)	
**Confidence in new technology**				1.000
Low	2 (5%)	1 (5%)	1 (6%)	
Moderate	20 (54%)	11 (58%)	9 (50%)	
High	15 (41%)	7 (37%)	8 (44%)	

© 2024 Cox CE et al. *JAMA Network Open.*

**Enthusiasm for a mobile app**				0.541
Low	5 (14%)	2 (11%)	3 (17%)	
Moderate	25 (68%)	12 (63%)	13 (72%)	
High	7 (19%)	5 (26%)	2 (11%)	
**Orientation to patient care**				1.000
Social and emotional primarily	24 (65%)	13 (68%)	11 (61%)	
Technical and scientific primarily	13 (35%)	6 (32%)	7 (39%)	
**Preferred style for decision making**				0.206
Lead patients and families	6 (16%)	5 (26%)	1 (6%)	
Engage as an equal partner	26 (70%)	12 (63%)	14 (78%)	
Provide information to patients and families for them to make their own decisions	5 (14%)	2 (11%)	3 (17%)	
**Confidence in communicating**				1.000
Low	0	0	0	
Moderate	11 (30%)	5 (26%)	6 (33%)	
High	26 (70%)	14 (74%)	12 (67%)	
**Preference for handling palliative care issues**				0.277
By myself	13 (35%)	8 (42%)	5 (28%)	
Palliative care team	2 (5%)	0	2 (11%)	
Mostly by myself with the palliative care team for difficult cases	22 (59%)	11 (58%)	11 (61%)	

*Stratification variables for randomization.

© 2024 Cox CE et al. *JAMA Network Open.*

eTable 2. Family Member and Patient Characteristics by Family Member Race

	Total<br>n=111	Black<br>n=47	White<br>n=64
**Family member characteristics**			
**Age**			
Mean (SD) years	51 (15)	50 (15)	52 (14)
median (IQR) years	52 (43, 61)	52 (41, 58)	54 (44, 62)
**Gender at birth**, n (%)			
Female	92 (83%)	43 (91%)	49 (77%)
Male	19 (17%)	4 (9%)	15 (23%)
**Gender described**, n (%)			
Woman	96 (86%)	45 (96%)	51 (80%)
Man	15 (14%)	2 (4%)	13 (20%)
**Education**, n (%)			
Completed High School or Below	20 (18%)	8 (17%)	12 (19%)
Bachelor's Degree or Other College-Related Education	68 (61%)	30 (64%)	38 (59%)
Graduate or Professional Degree	23 (21%)	9 (19%)	14 (22%)
**Marital Status**			
Married or live with a Partner	67 (60%)	21 (45%)	46 (72%)
Separated, divorced	19 (17%)	8 (17%)	11 (17%)
Widowed	4 (4%)	2 (4%)	2 (3%)
Single	21 (19%)	16 (34%)	5 (8%)
**Financial distress**, n (%)			
After paying the bills, you still have enough money for special things that you want	45 (41%)	16 (34%)	29 (45%)
You have enough money to pay the bills, but little spare money to buy extra or special things	44 (40%)	18 (38%)	26 (41%)
You have money to pay the bills, but only because you have cut back on things	13 (12%)	10 (21%)	3 (5%)

© 2024 Cox CE et al. *JAMA Network Open.*

	Total<br>n=111	Black<br>n=47	White<br>n=64
You are having difficulty paying the bills, no matter what you do	9 (8%)	3 (6%)	6 (9%)
**Relationship to patient**, n (%)			
Spouse or Partner	50 (45%)	14 (30%)	36 (56%)
Child	22 (20%)	13 (28%)	9 (14%)
Parent	23 (21%)	9 (19%)	14 (22%)
Brother or Sister	11 (10%)	8 (17%)	3 (5%)
Other	5 (5%)	3 (6%)	2 (3%)
**Employment**, n (%)			
Working Full Time or Part Time	74 (67%)	33 (70%)	41 (64%)
Homemaker Full Time	7 (6%)	0	7 (11%)
Unemployed	4 (4%)	3 (6%)	1 (2%)
Retired	19 (17%)	7 (15%)	12 (19%)
Disabled	7 (6%)	4 (9%)	3 (5%)
**Expected hospital survival by family member**, n (%)			
Almost certainly	56 (50%)	31 (66%)	25 (39%)
Most likely	47 (42%)	14 (30%)	33 (52%)
Probably or almost definitely not	8 (7%)	2 (4%)	6 (10%)
**Relationship with ICU physician,** n (n%)			
Excellent	46 (41%)	14 (30%)	32 (50%)
Good	43 (39%)	20 (43%)	23 (36%)
Acceptable	16 (14%)	7 (15%)	9 (14%)
Poor	6 (5%)	6 (13%)	0 (0%)
**ICU physician discussed my needs in a formal meeting,** n (n%)			
Yes	49 (44%)	22 (47%)	27 (42%)
No	59 (53%)	24 (51%)	35 (55%)
Missing	3 (3%)	1 (2%)	2 (3%)

© 2024 Cox CE et al. *JAMA Network Open.*

	Total n=111	Black n=47	White n=64
**Family meeting documented in medical chart after enrollment,** n (n%)			
Yes	57 (51%)	29 (62%)	28 (44%)
No	9 (8%)	1 (2%)	8 (13%)
Missing	45 (41%)	17 (36%)	28 (44%)
**Patient characteristics**			
**Age**, median (IQR) years	56.4 (44.3, 65.8)	57.2 (48.3, 66.8)	55.3 (43.4, 65.4)
**Gender**, n (%)			
Female	45 (41%)	20 (43%)	25 (39%)
Male	66 (59%)	27 (57%)	39 (61%)
**Race**, n (%)			
Black or African American	45 (41%)	44 (94%)	1 (1.5%)
White	65 (59%)	3 (6%)	62 (97%)
American Indian or Alaska Native	1 (1%)	0 (0%)	1 (1.5%)
**Insurance status**, n (%)			
Medicare	41 (37%)	16 (34%)	25 (39%)
Medicaid	22 (20%)	12 (26%)	10 (16%)
Commercial	36 (32%)	14 (30%)	22 (34%)
None	9 (8%)	4 (9%)	5 (8%)
Veteran’s Administration	3 (3%)	1 (2%)	3 (3%)
**ICU**			
Medical	61 (55%)	19 (40%)	42 (66%)
Neurological	20 (18%)	10 (21%)	10 (16%)
Surgical	17 (15%)	7 (15%)	10 (16%)
Cardiac	13 (12%)	11 (23%)	2 (3%)
**ICU admission source**			

© 2024 Cox CE et al. *JAMA Network Open.*

	Total n=111	Black n=47	White n=64
Emergency room	47 (42%)	28 (60%)	19 (30%)
Hospital ward	9 (8%)	3 (6%)	6 (9%)
Transfer from outside hospital	50 (45%)	15 (32%)	35 (55%)
Post-operative	4 (4%)	1 (2%)	3 (5%)
Clinic	1 (1%)	0	1 (2%)
**Primary ICU Admission Diagnosis**			
Shock	16 (14%)	8 (17%)	8 (13%)
Acute respiratory failure	53 (48%)	20 (43%)	33 (52%
Renal Failure	3 (3%)	1 (2%)	2 (3%)
Liver Failure	1 (1%)	1 (2%)	0
Acute neurological even / altered mental status	24 (22%)	13 (28%)	11 (17%)
Trauma / post-operative	14 (13%)	4 (9%)	10 (16%)
**APACHE II score**, median (IQR) points	23.0 (1.0, 28.0)	23.0 (17.0, 31.0)	23.0 (16.5, 26.0)
**Expected hospital survival by ICU physician**, n (%)			
Almost certainly	10 (9%)	5 (11%)	5 (8%)
Most likely	30 (27%)	9 (19%)	21 (33%)
Probably or almost definitely not	6 (5%)	1 (2%)	5 (8%)
Missing	61 (55%)	31 (66%)	30 (47%)
**ICU physician reported discussing needs with family in formal family meeting**			
Yes	39 (35%)	11 (23%)	28 (44%)
No	11 (10%)	5 (11%)	6 (9%)
Missing	61 (55%)	31 (66%)	30 (47%)
**ICU physician self-described relationship with family**			
Excellent	17 (15%)	5 (11%)	12 (19%)
Acceptable	3 (3%)	1 (2%)	2 (3%)
Good	28 (25%)	9 (19%)	19 (30%)

© 2024 Cox CE et al. *JAMA Network Open.*

	Total<br>n=111	Black<br>n=47	White<br>n=64
Poor	2 (2%)	1 (2%)	1 (2%)
Missing	61 (55%)	31 (66%)	30 (47%)
**Chronic medical comorbidities**, median (IQR) number of comorbidities	1.0 (1.0, 2.0)	2.0 (1.0, 3.0)	1.0 (1.0, 2.0)
**Tracheotomy during hospitalization,** n (%)	47 (42%)	17 (36%)	30 (47%)
**Code status at ICU admission**			
Full code	109 (98%)	47 (100%)	62 (97%)
DNAR	2 (2%)	0	2 (3%)
**Code status at hospital discharge**			
Full code	79 (71%)	40 (85%)	39 (61%)
DNAR	31 (28%)	7 (15%)	24 (38%)
Missing	1 (1%)	0	1 (2%)
**Code status during hospitalization**			
Full code throughout	79 (71%)	40 (85%)	39 (62%)
DNAR throughout	2 (2%)	0	2 (3%)
Full code to DNAR	29 (26%)	7 (15%)	22 (35%)
DNAR to full code	0	0	0
Missing	1 (1%)	0	1 (2%)
**Palliative care trigger present**, n (%)			
No	81 (73%)	38 (81%)	43 (67%)
Yes	30 (27%)	9 (19%)	21 (33%)
Multisystem organ failure that worsened over 48 hours from ICU admission	18 (16%)	8 (17%)	10 (16%)
Devastating neurological injury	10 (9%)	6 (13%)	4 (6%)
≥3 activities of daily living limitations	3 (3%)	0 (0%)	3 (5%)
Cardiac arrest	3 (3%)	2 (4%)	1 (2%)
Advanced cancer	4 (4%)	1 (2%)	3 (5%)

© 2024 Cox CE et al. *JAMA Network Open.*

	Total<br>n=111	Black<br>n=47	White<br>n=64
≥1 ICU admission within 3 mo.	5 (5%)	2 (4%)	3 (5%)
≥2 hospital admissions within 3 mo.	9 (8%)	5 (11%)	4 (6%)
Dementia	0	0	0
Admitted from post-acute care facility	5 (5%)	2 (4%)	3 (5%)
**Palliative care specialist consultation during hospitalization**, n (%)			
No	97 (87%)	42 (89%)	55 (86%)
Yes	14 (13%)	5 (11%)	9 (14%)
**Ventilator status at discharge,** n (%)			
Liberated from ventilator	72 (65%)	32 (68%)	40 (63%)
Ventilator dependent	17 (15%)	10 (21%)	7 (11%)
Extubated for comfort care	22 (20%)	5 (11%)	17 (27%)

© 2024 Cox CE et al. *JAMA Network Open.*

eTable 5. Needs Reported by Family Members in Their Own Words

The following table reflects needs written in family members' own words. They are arranged in five general themes below.

Environment and visitation

"Quiet hours" or visiting restrictions grossly impede our family's ability to provide support for our loved one, and enforcement of these policies does not seem uniform across the unit or staff. An open ICU policy would make our family feel more welcome and involved in care.
accomidations for family members in the ICU waiting rooms; would like pull out beds or cots
Arrangements in waiting room to be more accommodating to long term visits
Better housekeeping/ floor mopped/ bathroom mopped
can't visit him because of covid
Concern with admission to ICU from the ER, I was still sitting in the ER when he was transferred to the ICU and no one informed me. I didn't find out that he was transferred until I asked the front desk again. They then informed me that visiting hours were over. However, the social worker came down and took me up to the floor. There was added stress because I had to leave my 16 year old in the ER by herself because they would not let her in.
everything has been addressed appropriately and a timely fashion; I trust the doctors. My biggest frustration is not being able to come to the hospital to see my loved one
Family accommodation of long-term stay patients of MICU. Family's of cancer and under 21 yrs old have wonderful care in this area. It is a great blessing for those families.
Having a patient/loved one "medical" advocate
I know and understand that for everyone protection with the COVID 19 going around that everyone needs to be protected but I think if there is a patient that is a young adult and is in serious condition that both parents should be able to see them at different times of visiting hours in other words with and 8 hour visiting time have it divided into 4 hrs a piece and both can't be in hospital at same time unless it's clearly life threatening
I know that COVID has caused a lot of concern for the healthcare and families whom have become ill. Believe me we are dealing with it right now with our 82 year old Dear Mother! I also know that are rules to protect the doctors, nurses, patients, visitors, and other employees at the hospital. I think it is cruel and unnecessary to keep children from taking turns and visiting there mother who is in intensive care whom is 82 years old, on a respirator, dialysis, oxygen, lung infection, etc... Our mother wants to see all of her 3 girls because the Lord may take her! It is heartbreaking that we live 4 hours away and my sister who is staying at the Lodge across the street can't come see her mother who is so sick! They should allow sisters to take turns to be with their mother! Give them a COVID Test in these circumstances but don't keep children away from their failing parents. That puts that one sibling in a position to get so tired and worried when they could take turns and can help during this sad time! We live 4 hours away and my sister sits and waits! Shame!
I wish I could visit my loved one. While I completely understand the need for the COVID-19 restrictions, it is very hard on families to not be able to be with their loved ones.
I would like an opportunity to put my Dad phone to his ear so that he could hear my voice
I would like to see my wife please
Information about parking, location of food, etc was not given for 4 days
LAR does not like the protocol for re-testing for COVID. Feels like re-testing should be done more often.
Noise level in the unit is always high.
parking
Restrictions on number of visitors after 9 pm have placed a significant hardship when coping with the stress and trauma of the ICU.
Validation for parking especially for family's in icu
Waiting room sleep and comfort conditions arent the best and very uncomfortable
Wishes she could visit her husband. COVID restrictions are tight.

© 2024 Cox CE et al. *JAMA Network Open.*

Patient related needs

I wish there was more that can be done for his extreme dry mouth situation, but I understand that it is probably not possible.
[*patient name*] is on a ventilator. Therefore I expect her level of comfort to improve once she is extubated.
long term care for my son
My concern is that he is not improving
The long term afect
The plans cannot be talked at length because his day to day condition
Understanding the treatment of elderly and mentally challenged individuals. Would prefer to have everyone understand a 92 years old who walked into hospital bright and alert but suffering for dementia. Yes she had fractured ribs which can be a life threatening issue but is not always fatal. Who currently is recovering but suffers when care giver is unable to stay bedside. Faith and encourage along with care and compassion means more sometimes than doctors acknowledge.

Family member related needs

Financial assistance
Financial burdens
Nothing the hospital can help with. Our car is too old to drive from [*name of town*] and back daily so Amanda is alone. I know she is well taken care of but it is just awful not being there. We have other children at home so we are torn between what we want...what's best for everyone....what's rational, etc. Do we get a hotel and rent a vehicle...rent an apt......visit once a week? Just awful
The responses to the survey was completed over the phone with the CRC asking the participant these questions. The participant stated that because of his father's lack of English proficiency and speaking a certain dialect of Portuguese, he felt that there were many communication barriers with the patient and the clinical team. The participant often translates for the patient. The participant wishes that he could be at the patient's bedside more but was told that he could only visit for certain time of day. The participant feels that he is discouraged to come in to the hospital in person and due to this he's having to be updated via phone calls he receives from the clinical team which causes him anxiety.
Worried about financial situation in the future, treatments, medications, travel, etc
consider my knowledge of the patient in a personal perspective of his mental + emotional sate as well as physical

Physician, nurse, and staff related needs

Better English not so much medical terms
Calls are not frequent, and usually from PA and only specialist doctor, not attending
Communication from medical team needs to improve. Family should not have to continuously call for updates on their loved one.
Consider the effect of the Resident and Fellow
During the weekend it was hard to get an answer from the nurse's station or doctors to call me
Ensuring that every effort is being made to communicate on updates
Have not heard from a doctor only NP and I had to come all the way to hospital for that phone call to be made.
Honestly the proper understanding about my mom's care and understanding if the procedure there saying she needs is a long term or short. Also the understanding of her treatments. The nurses being more aware of the patients needs and not allowing them to sit in there bowls moments. Also being patient with the family when they are asking questions without giving attitude. Nurses negative thoughts.
I dont know who the doctors are. I never met a single one
I feel that procedures are often pushed to be done without considering that my brother can heal and get better but with adequate time, I feel that his needs are not always considered because it is an assumption that he doesnt understand but he does. I also dont like that he is sometime considered by doctors as having a

I feel that the times the drs do rounds should be closer to the times that the family is allowed in my husband has been here four days and today was the first day that I have really spoke to a dr also sometimes instead of using the words we have to wait and see it's easier to say what you think in your medical opinion instead of telling a family member it takes time it's easier to hear it straight out then living in the unknown. I was here with my husband and all I wanted to know is if he would walk again and instead of telling me the odds I was told it's a waiting thing until I got furious and asked straight out for his odds sometimes it's easier to think our lives ones will beat the odds.
I have concerns with the transfer (or lack) of information from other health care providers.
I have not communicated with a neurologist, but have been told there is documentation in the chart when I was in the room but I was not aware they were here because they did not come in to room.
I have one shot with a doctor in the morning, would be nice to have access out
I learned that he had a rough event only after arriving at the room. However I was informed promptly by the nurse when I arrived.
I recently stated a concern and I felt I was blown off.
I tried to reach the nurses by calling sometimes, but it can be hard to reach the nurses by phone at times
I wish doctors would stop by more often just to fill patients in on progress and test results.
I feel that the times the drs do rounds should be closer to the times that the family is allowed in my husband has been here four days and today was the first day that I have really spoke to a dr also sometimes instead of using the words we have to wait and see it's easier to say what you think in your medical opinion instead of telling a family member it takes time it's easier to hear it straight out then living in the unknown. I was here with my husband and all I wanted to know is if he would walk again and instead of telling me the odds I was told it's a waiting thing until I got furious and asked straight out for his odds sometimes it's easier to think our lives ones will beat the odds.
I have concerns with the transfer (or lack) of information from other health care providers.
I feel that the times the drs do rounds should be closer to the times that the family is allowed in my husband has been here four days and today was the first day that I have really spoke to a dr also sometimes instead of using the words we have to wait and see it's easier to say what you think in your medical opinion instead of telling a family member it takes time it's easier to hear it straight out then living in the unknown. I was here with my husband and all I wanted to know is if he would walk again and instead of telling me the odds I was told it's a waiting thing until I got furious and asked straight out for his odds sometimes it's easier to think our lives ones will beat the odds.
I have concerns with the transfer (or lack) of information from other health care providers.
I have not communicated with a neurologist, but have been told there is documentation in the chart when I was in the room but I was not aware they were here because they did not come in to room.
I have one shot with a doctor in the morning, would be nice to have access out
I learned that he had a rough event only after arriving at the room. However I was informed promptly by the nurse when I arrived.
I recently stated a concern and I felt I was blown off.
I tried to reach the nurses by calling sometimes, but it can be hard to reach the nurses by phone at times
I wish doctors would stop by more often just to fill patients in on progress and test results.
I wish that a doctor would call us once a day, at least until my sister regains consciousness.
I wish that they would call more often when they find out something new
I would like a detailed description of care plan
I'm not always able to be physically present. I would appreciate a phone call after rounds to get any updates on my loved ones condition.
ICU nurses are not being nice to my grandmother all the time. My grandmother stated " these people are mean" when discussing her nurses. It seems that the night shift nurse are more aggressively holding my grandmother and they doing a lot of things to her without telling her what they are doing.
Inexperienced nurses with complex patients, more supervised training.
Info on where the patient will be in terms of how things may play out
It's hard to remember details from dialogues with the care team; would it be possible to generate a simple summary of current status and plans

© 2024 Cox CE et al. *JAMA Network Open.*

Just a note that I speak more frequently with nurses than doctors and the doctor recently changed. The previous doctor was very helpful, but I don't know the new one we'll because I haven't been here at the same time the new doctor has checked on the patient.
Just being able to communication with the doctors
Little communication from MD. Mostly from PAs & RN.
not regular communication with ICU MDs conflicting information between RNs and shifts. No plan for the day to set expectations for ourselves. Had to chase down MDs
Not sure there is time for me to ask all my questions, so I pick the most pressing.
patient is cared for when family is around. patient called for 1 hour without response. often when request to speak to a doctor it is not acted upon quickly and a lot of bullshit.
The ICU nurses are wonderful and a great resource. Charge nurse, however, is very distant and does not acknowledge a patient 's family in the room or conversation.The difference in approach was jarring, given the responsiveness of the rest of the team.
They need to let family members know what is going on with their loved one. Understand that they are busy, but needs to be more open communication.
They refuse to provide information on my chart.
This survey should include questions about how the age of the family member effects communication.
Unsure of how to pay medical bills
When you rarely see the doctor it is hard to answer these questions. I also asked the doctors to call me the last 3 days my daughter was in ICU and I never have received that call. I did talk to a PA once never to a doctor eccept the 1st night she was there before surgery.
Whenever a nurse relieves the nurse assigned he/she needs to be sure who is allowed to visit and receive information regarding the patient.
Would like to get written report of diagnosis & procedures
would like more updates on my husband

Positive comments

All needs have been addressed
I am extremely pleased with the care we have received thus far
I am uniquely situated given that my husband (patient)is a physician himself & the vast team of Doc's whom currently (& previously) look after him, all communicate and keep me well informed.
I am very satisfied with the ICU doctors
So far the staff have been very kind
ICU team is like family.
The ICU team has made me and the family feel just as much apart of the team as they are.
The ICU team is awesome! I'm very happy with my brothers care and our family involvement
They always come back if they must finish up what they are doing

eTable 6. Outcomes by Race and Study Group

Outcome	Black family members: Intervention *n=19* Mean (SD)	Black family members: Control *n=28* Mean (SD)	Black family members: Estimated difference, intervention - control (95% CI)	Black family members: *p* value	White family members: Intervention *n=36* Mean (SD)	White family members: Control *n=28* Mean (SD)	White family members: Estimated difference, intervention - control (95% CI)	White family members: *p* value	*Intervention vs Control for Black vs White family members*	*p value*
**NEST *(primary outcome)***										
Day 1	42.6 (27.2)	38.0 (19.6)			33.9 (12.3)	34.5 (15.0)				
Day 3	33.9 (22.9)	29.2 (20.7)			22.8 (16.1)	35.3 (23.4)				
Day 7	30.3 (26.7)	28.0 (21.6)			22.3 (14.5)	31.2 (18.9)				
Change, Day 3 – Day 1			-0.3 (-9.3, 8.8)	0.96			-12.5 (-18.9 - 6.1)	0.0002	12.2 (1.6, 22.8)	0.02
Change, Day 7 – Day 1			-1.4 (-10.7, 7.8)	0.76			-9.5 (-16.1, -3.0)	0.005	8.1 (-2.7, 18.9)	0.14
**PHQ-9**										
Day 1	7.4 (3.9)	7.7 (3.7)			8.3 (3.3)	7.7 (3.5)				
Day 3	6.1 (4.0)	6.1 (2.9)			7.4 (3.6)	6.7 (3.4)				
Month 3	5.1 (3.2)	6.1 (3.3)			6.4 (3.4)	6.0 (3.4)				
Change, Day 3 – Day 1			0.2 (-1.1, 1.5)	0.79			0.2 (-0.9, 1.3)	0.72	-0.2 (-2.4, 1.9)	0.82
Change, Month 3 – Day 1			-0.8 (-2.8, 1.2)	0.40			0.2 (-1.5, 1.8)	0.86	-1.0 (-3.2, 1.3)	0.41
**GAD-7**										
Day 1	9.2 (7.2)	7.3 (5.7)			9.2 (5.9)	9.5 (6.0)				

© 2024 Cox CE et al. *JAMA Network Open.*

Day 3	7.7 (6.5)	6.5 (5.1)			8.1 (6.0)	8.4 (5.7)				
Month 3	5.2 (5.2)	4.3 (4.8)			6.5 (5.2)	6.3 (5.1)				
Change, Day 3 – Day 1			-0.5 (-2.1, 1.1)	0.55			0.0 (-1.8, 1.8)	0.99	-0.3 (-3.7, 3.2)	0.88
Change, Month 3 – Day 1			0.1 (-3.0, 3.2)	0.96			0.5 (-1.9, 2.9)	0.68	-1.1 (-4.8, 2.5)	0.54
**PTSS**										
Day 1	25.1 (17.9)	24.4 (14.3)			26.8 (13.2)	24.6 (13.1)				
Month 3	22.9 (17.1)	20.8 (13.4)			26.5 (15.1)	25.3 (14.3)				
Change, Month 3 – Day 1			2.2 (-6.6, 10.9)	0.62			0.4 (-5.1, 5.9)	0.88	1.7 (-8.0, 11.4)	0.73
**QOC**										
Day 1	7.4 (3.2)	7.5 (3.0)			8.3 (2.0)	8.4 (1.7)				
Day 3	8.6 (1.9)	8.5 (2.2)			9.1 (1.7)	8.3 (2.0)				
Change, Day 3 – Day 1			0.08 (-1.2, 1.4)	0.91			1.0 (0.1, 1.8)	0.02	-0.7 (-2.1, 0.7)	0.31
**IPC / Eliciting Concerns** [1]										
Day 1 N (%)	13(68%)	23 (82%)			33 (92%)	24 (86%)				
Day 3 N (%)	15 (83%)	22 (79%)			34 (94%)	22 (81%)				

© 2024 Cox CE et al. *JAMA Network Open.*

Odds Ratio of Intervention vs Control, Day 3 – Day 1			2.4 (0.5, 11.2)	0.27			3.7 (0.7, 20.2	0.13	n/a	n/a
**IPC / Decision making**										
Day 1	3.4 (1.4)	3.6 (1.4)			3.7 (1.1)	3.8 (1.1)				
Day 3	3.6 (1.2)	3.4 (1.5)			4.0 (1.0)	3.7 (1.1)				
Change, Day 3 – Day 1			0.22 (-0.53, 0.98)	0.55			0.3 (-0.1,0.8)	0.18	-0.04 (-0.9, 0.8)	0.92
**IPC / Discrimination** [2]										
Day 1	2 (11%)	5 (18%)			2 (6%)	2 (7%)				
Day 3	3 (17%)	6(21%)			0 (0%)	1 (4%)				
Odds ratio of intervention vs. control (95% CI), day 3			n/a	n/a			n/a	n/a	n/a	n/a
**Goal concordant care**										
Day 1	14 (74%)	26 (93%)			23 (64%)	24 (86%)				
Day 3	18 (95%)	23 (82%)			29 (81%)	24 (86%)				
Odds ratio of intervention vs. control (95% CI), day 3			5.9 (0.5, 74.4)	0.17			0.9 (0.2, 3.4)	0.93	n/a	n/a

[1] The IPC Eliciting Concerns score was dichotomized at 5 vs <5.
[2] The IPC Discrimination score was dichotomized at 1 vs >1. Due to the small number of events in each arm and time point, a longitudinal model was not fit to this outcome; no statistical test was conducted; no statistical test was conducted.

© 2024 Cox CE et al. *JAMA Network Open.*

eTable 3. Clinical Characteristics and Clinical Outcomes

	**Total** n=111	**Intervention** n=55	**Control** n=56
**ICU**			
Medical	61 (55%)	34 (62%)	27 (48%)
Neurological	20 (18%)	5 (9%)	15 (27%)
Surgical	17 (15%)	10 (18%)	7 (13%)
Cardiac	13 (12%)	6 (11%)	7 (13%)
**ICU admission source**			
Transfer from outside hospital	50 (45%)	26 (47%)	24 (43%)
Emergency room	47 (42%)	22 (40%)	25 (45%)
Hospital ward	9 (8%)	5 (9%)	4 (7%)
Post-operative	4 (4%)	2 (4%)	2 (4%)
Clinic	1 (1%)	0 (0%)	1 (2%)
**Primary ICU Admission Diagnosis**			
Shock	30 (27%)	14 (25%)	16 (29%)
Myocardial infarction	1 (1%)	1 (2%)	0
Cardiac dysrhythmias	7 (6%)	6 (11%)	1 (2%)
ARDS	14 (13%)	9 (16%)	5 (9%)
Pneumonia	45 (41%)	23 (42%)	22 (39%)
Sepsis	23 (21%)	18 (13%)	10 (18%)
Gastrointestinal bleeding	2 (2%)	1 (2%)	1 (2%)
Pancreatitis	2 (2%)	0	2 (4%)
Intestinal obstruction	3 (3%)	2 (4%)	1 (2%)
Acute renal failure, no acute dialysis	4 (4%)	3 (5%)	1 (2%)
Acute renal failure, required acute dialysis	15 (14%)	10 (18%)	5 (9%)
Surgical procedure	5 (5%)	3 (5%)	2 (4%)
Cancer complication	2 (2%)	1 (2%)	1 (2%)

© 2024 Cox CE et al. *JAMA Network Open.*

	Total<br>n=111	Intervention<br>n=55	Control<br>n=56
Altered mental status / encephalopathy	11 (10%)	4 (7%)	7 (13%)
Stroke or intracerebral hemorrhage	11 (10%)	4 (7%)	7 (13%)
Seizures	4 (4%)	1 (2%)	3 (5%)
Other	32 (29%)	15 (27%)	17 (30%)
**Secondary ICU Admission Diagnoses**			
Shock	16 (14%)	11 (20%)	5 (9%)
Acute respiratory failure	53 (48%)	25 (45%)	28 (50%)
Renal Failure	3 (2.7%)	3 (5%)	0 (0%)
Liver Failure	1 (0.9%)	0 (0%)	1 (2%)
Acute neurological event / altered mental status	24 (22%)	8 (15%)	16 (29%)
Trauma / post-operative	14 (13%)	8 (15%)	6 (11%)
**Acquired hospital conditions**			
Acute liver failure	1 (1%)	1 (2%)	0
Myocardial infarction	3 (3%)	1 (2%)	2 (4%)
Acute respiratory distress syndrome	7 (6%)	5 (9%)	2 (4%)
Bacteremia	7 (6%)	2 (4%)	5 (9%)
Cardiac arrest	2 (2%)	1 (2%)	1 (2%)
Cardiac dysrhythmias requiring treatment	5 (5%)	3 (5%)	2 (4%)
Acute renal failure requiring acute dialysis	4 (4%)	1 (2%)	3 (5%)
Pneumonia	18 (16%)	8 (15%)	10 (18%)
Shock	7 (6%)	3 (5%)	4 (7%)
Decubitus ulcer	2 (2%)	1 (2%)	1 (2%)
Surgical procedure	9 (8%)	4 (7%)	5 (9%)
Pulmonary embolism or deep venous thrombosis	5 (5%)	5 (9%)	0
Stroke	2 (2%)	2 (4%)	0
None	70 (63%)	32 (58%)	38 (68%)

© 2024 Cox CE et al. *JAMA Network Open.*

	**Total** n=111	**Intervention** n=55	**Control** n=56
**Major surgery**, n (%)	42 (38%)	24 (44%)	18 (32%)
**Surgery acuity**, n (%)			
Emergency	19 (17%)	5 (9%)	14 (25%)
Elective	23 (21%)	19 (35%)	4 (7%)
**APACHE II score on day of randomization**, median (IQR), units	23.0 (17.0, 28.0)	23.0 (18.0, 28.0)	21.0 (16.0, 27.0)
**Palliative care clinical trigger present**			
No	72 (65%)	32 (58%)	40 (71%)
Yes	39 (35%)	23 (42%)	16 (29%)
Multisystem organ failure that worsened over 48 hours from ICU admission	18 (16%)	7 (13%)	11 (20%)
Devastating neurological injury	10 (9%)	2 (4%)	8 (14%)
≥3 activities of daily living limitations	3 (3%)	1 (2%)	2 (4%)
Cardiac arrest	3 (3%)	1 (2%)	2 (4%)
Advanced cancer	4 (4%)	3 (5%)	1 (2%)
≥1 ICU admission within 3 mo.	5 (4.5%)	3 (5%)	2 (4%)
≥2 hospital admissions within 3 mo.	9 (8.1%)	3 (5%)	6 (11%)
Dementia	0	0	0
Admitted from post-acute care facility[5]	5 (5%)	4 (7%)	1 (2%)
**Total number palliative care triggers present**, median (IQR), no.	1.0 (0, 1.0)	1.0 (0, 1.0)	1.0 (0, 1.0)
**Mechanical ventilation duration**, median (IQR), days	10.0 (6.0, 18.0)	9.0 (5.0, 14.0)	11.5 (6.5, 20.5)
**Tracheotomy**	47 (42%)	24 (44%)	23 (41%)

© 2024 Cox CE et al. *JAMA Network Open.*

	Total<br>n=111	Intervention<br>n=55	Control<br>n=56
**Ventilation status at discharge**			
Liberated from ventilator	72 (65%)	33 (60%)	39 (70%)
Ventilator dependent	17 (15%)	8 (15%)	9 (16%)
Extubated for comfort care	22 (20%)	14 (25%)	8 (14%)
**ICU duration**, median (IQR), days	19.0 (11.0, 29.0)	17.0 (10.0, 29.0)	19.0 (11.5, 29.0)
**Hospital length of stay**, median (IQR), days	26.0 (18.0, 40.0)	26.0 (16.0, 42.0)	27.0 (19.5, 40.0)
**Procedures post-randomization**			
CPR	6 (5%)	2 (4%)	4 (7%)
Dialysis	17 (15%)	7 (13%)	10 (18%)
ECMO	17 (15%)	10 (18%)	7 (13%)
Intubation	50 (45%)	22 (40%)	28 (50%)
Major surgery	21 (19%)	13 (24%)	8 (14%)
Surgical feeding tube (i.e., PEG)	39 (35%)	18 (33%)	21 (38%)
Tracheotomy	37 (33%)	18 (33%)	19 (34%)
Vasopressors	32 (29%)	15 (27%)	17 (30%)
**Family meeting after randomization documented in medical record**			
Yes	57 (51%)	28 (51%)	29 (52%)
No	9 (8%)	4 (7%)	5 (9%)
Missing	45 (41%)	23 (42%)	22 (39%)
**Code status at ICU admission**			
Full code	108 (98%)	54 (98%)	55 (98%)
DNAR	2 (2%)	1 (2%)	1 (2%)

© 2024 Cox CE et al. *JAMA Network Open.*

	Total n=111	Intervention n=55	Control n=56
Missing	1 (1%)	0	1 (2%)
**Code status at discharge**			
Full code	79 (72%)	37 (67%)	42 (75%)
DNAR	31 (28%)	18 (33%)	13 (23%)
Missing	1 (1%)	0	1 (2%)
**Code status during hospitalization**			
Full code throughout	79 (72%)	37 (67%)	42 (75%)
DNAR throughout	2 (2%)	1 (2%)	1 (2%)
Full code to DNAR	29 (26%)	17 (31%)	12 (21%)
DNAR to full code	0	0	0
**Hospital survival**			
Alive	73 (70%)	38 (69%)	40 (71%)
Died	33 (30%)	17 (31%)	16 (29%)
**Discharge disposition**			
Home, independent	20 (18%)	8 (15%)	12 (21%)
Home with paid care	8 (7%)	4 (7%)	4 (7%)
Inpatient rehabilitation facility	13 (12%)	5 (9%)	8 (14%)
Skilled nursing facility	22 (20%)	14 (25%)	8 (14%)
Long-term acute care facility	10 (9%)	4 (7%)	6 (11%)
Transfer to other acute care hospital	1 (1%)	1 (2%)	0
Inpatient palliative care unit or inpatient hospice	3 (3%)	2 (4%)	1 (2%)
Other	1 (1%)	1 (2%)	0
Died	33 (30%)	17 (31%)	16 (29%)

© 2024 Cox CE et al. *JAMA Network Open.*

	**Total** n=111	**Intervention** n=55	**Control** n=56
**Died within 3 months of randomization,** n (%)	40 (36%)	21 (38%)	19 (34%)

© 2024 Cox CE et al. *JAMA Network Open.*

eTable 4. Consent and Retention Rates by Race and Group

	Black		White	
Consented[1]		80		110
Randomized		47 (59%)		64 (58%)
	**Intervention (n=19)**	**Control (n=28)**	**Intervention (n=36)**	**Control (n=28)**
**Day 7**	17 (89%)	27 (96%)	35 (97%)	26
**3 months**	15 (79%)	25 (89%)	34 (94%)	24

[1] Note: 5 consented family members ad missing race

© 2024 Cox CE et al. *JAMA Network Open.*

eFigure 1. Flow of Study Operations

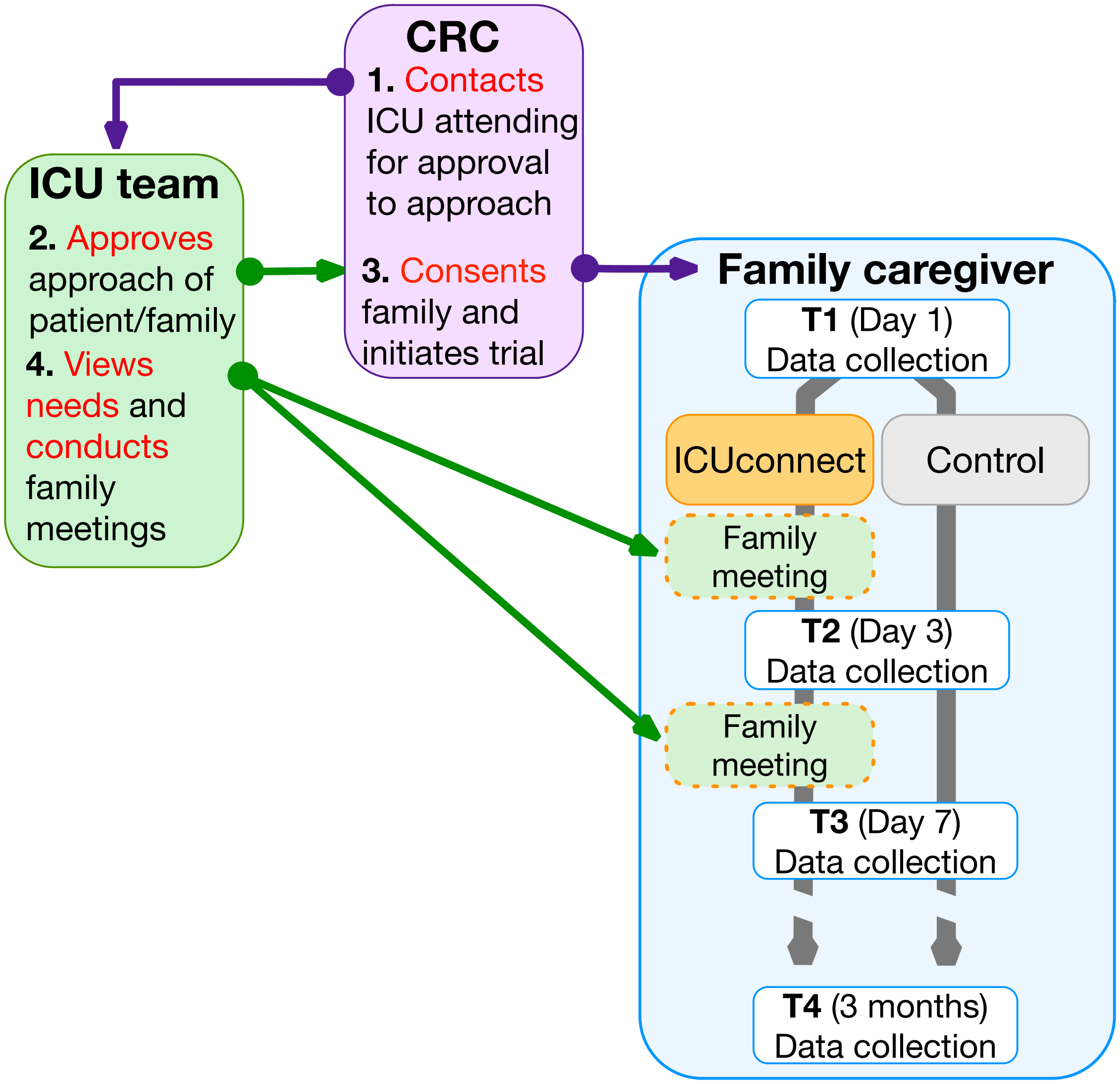

© 2024 Cox CE et al. *JAMA Network Open.*

eFigure 2. Mobile App Notification Strategy

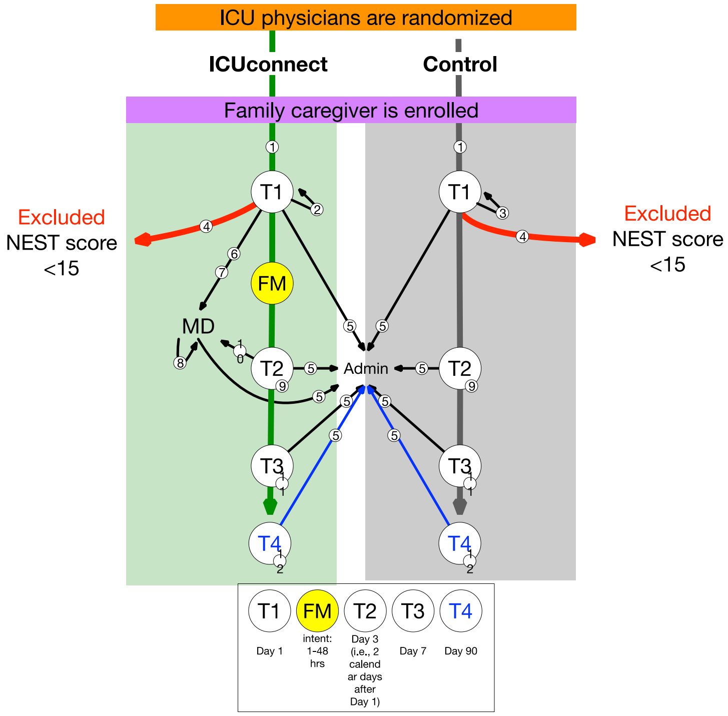

	Alert modality	Copy title	Who receives	Alert intiated by:	Alert fires initially:	Alert repeats:	Alert stops b/c:
①	text / email	Caregiver-T1 Interview [use same email for reminder]	Family member	Family enrollment / profile in app	Profile created and 'submit' pressed	12 hrs past 11:59pm of the day initial alert fires x 2 days	T1 completed by family member
②	text / email	Caregiver-Welcome to the ICUconnect study INTERVENTION	Family member	T1 completed by family member with intervention doctor	Exact time T1 completed by family member	N/A	N/A
③	text / email	Caregiver-Welcome to the ICUconnect study CONTROL	Family member	T1 completed by family member with control doctor	Exact time T1 completed by family member	N/A	N/A
④	text / email	Caregiver-ONLY FOR THOSE WITH NEST<15	Family member	Email	Exact time T1 completed by family member and NEST<15	N/A	N/A
⑤	text / email	text / email	Study staff	Family **or** doctor complete ANY survey	Exact time survey completed by family member (T1/T2/T3/T4) or doctor (T2)	N/a	N/a
⑥	text / email / page	ICU physician-T1 View NEST score	Doctor	T1 completion by family member	Exact time family completes T1	N/A	N/A
⑦	text / email / page	ICU physician-T1 View NEST score reminder	Doctor	T1 completion by family member but NEST not viewed by doctor	12 hours past 1159pm of day of T1 completion (i.e., noon of next calendar day)	2 days from 11:59pm of the calendar day family member completes T1	Doctor views T1
⑧	text / email / page	ICU Physician-Family meeting reminder	Doctor	T1 completion by family member	12 hours past 1159pm of day of T1 completion (i.e., noon of next calendar day)	None; only sent 1 time	N/a
⑨	text / email	Caregiver-T2 survey [use same email for reminder]	Family member	Time	2 days from 11:59pm of the calendar day family member completes T1	3 days past 11:59pm the calendar day family member completes T1 x 2 days	T2 completed by family member
⑩	text / email / page	ICU physician-T2 NEST score to view [use same email for reminder]	Doctor	Family member completes T2 survey	Exact time family completes T2	12 hrs past 11:59pm of the day T2 is completed x 2 days	Doctor completes doctor survey (and views family T2 data)
⑪	text / email	Caregiver-T3 survey [use same email for reminder]	Family member	Time	6 days from 11:59pm of the calendar day family member completes T1	7 days past 11:59pm the calendar day family member completes T1 x 2 days	T3 survey completed by family member
⑫	text / email	Caregiver-T4 survey [use same email for reminder]	Family member	Time	89 days from 11:59pm of the calendar day family member completes T1	90 days past 11:59pm the calendar day family member completes T1 x 2 days	T4 survey completed by family member

© 2024 Cox CE et al. *JAMA Network Open.*

eFigure 3. Screenshots of ICUconnect Mobile App

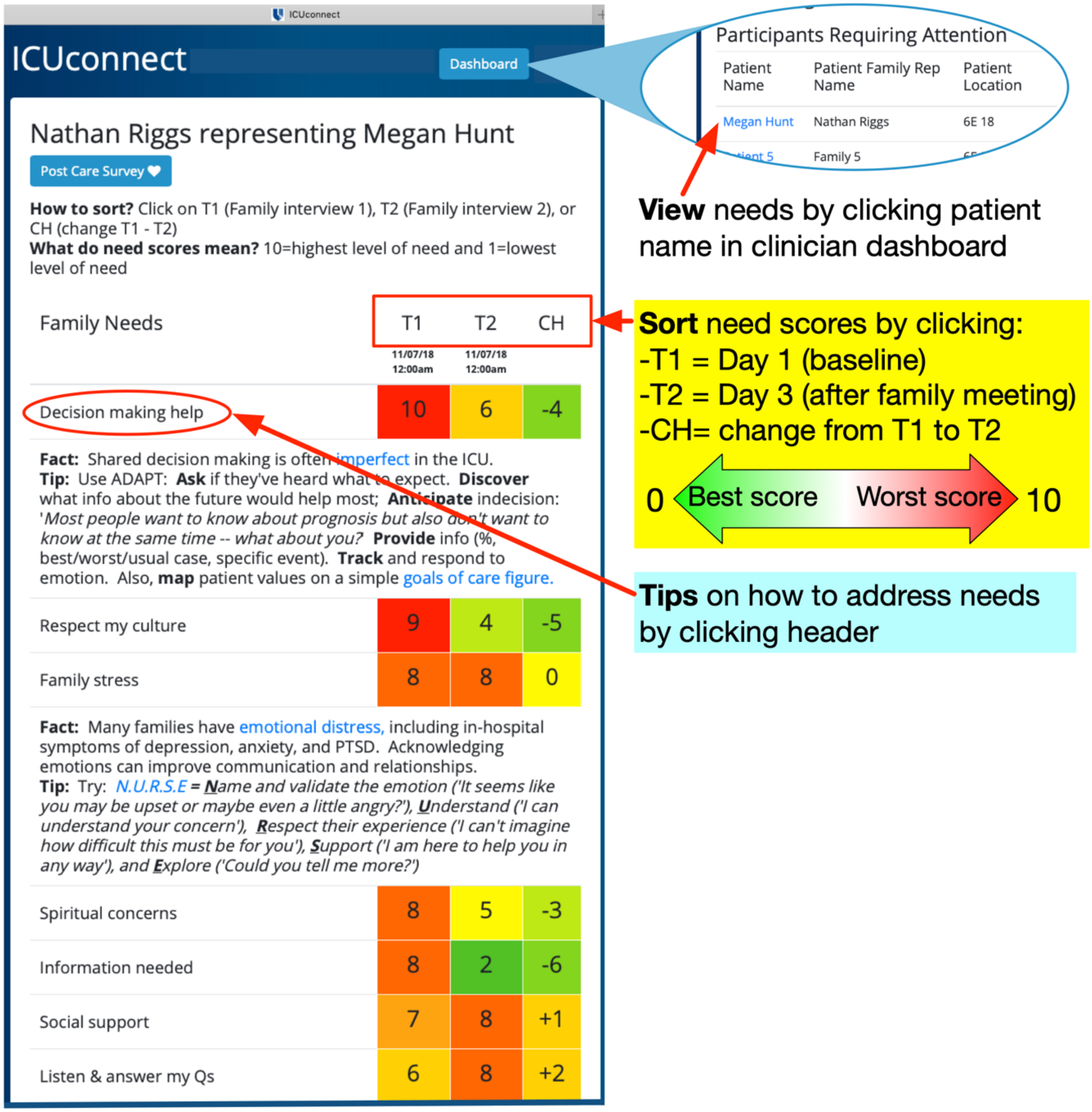

© 2024 Cox CE et al. *JAMA Network Open.*

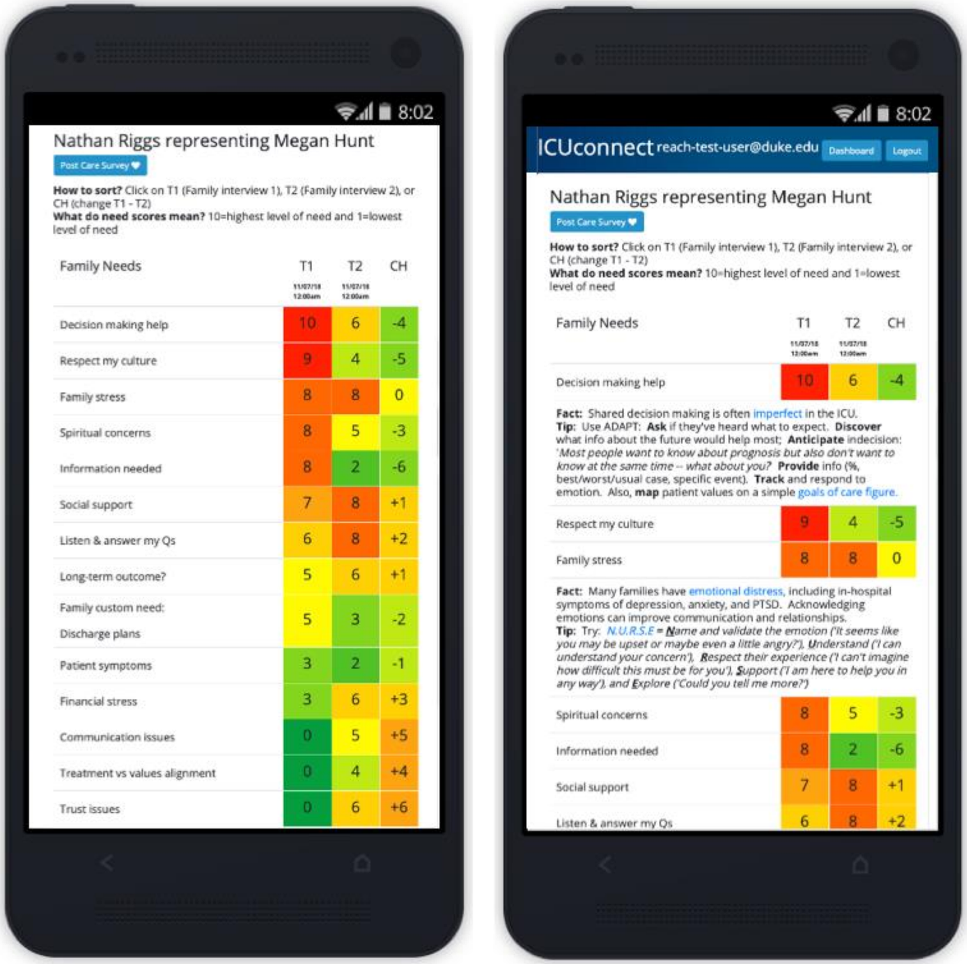

© 2024 Cox CE et al. *JAMA Network Open.*

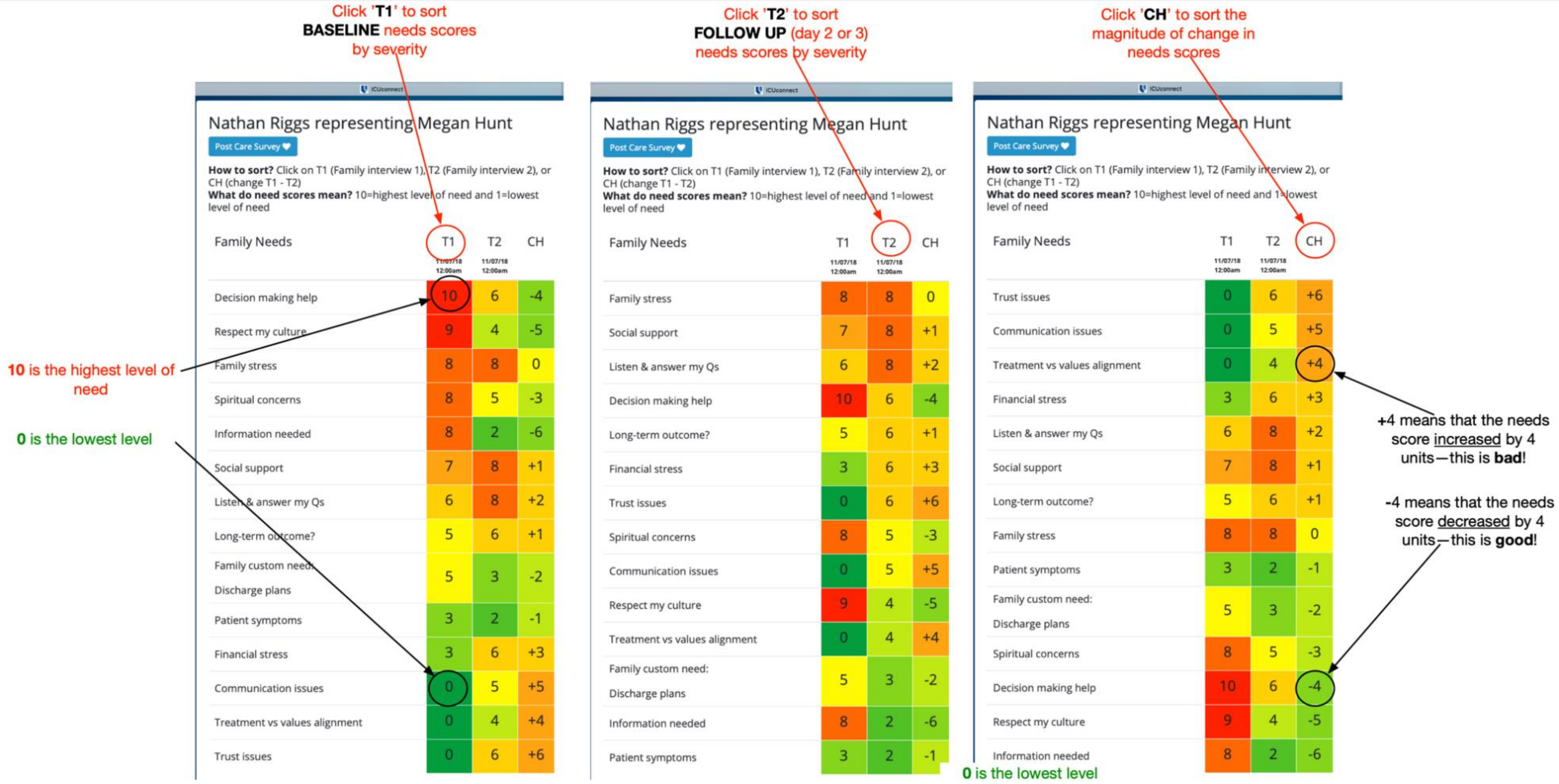

© 2024 Cox CE et al. *JAMA Network Open.*

eFigure 4. Primary Outcome by Treatment Group and Physician

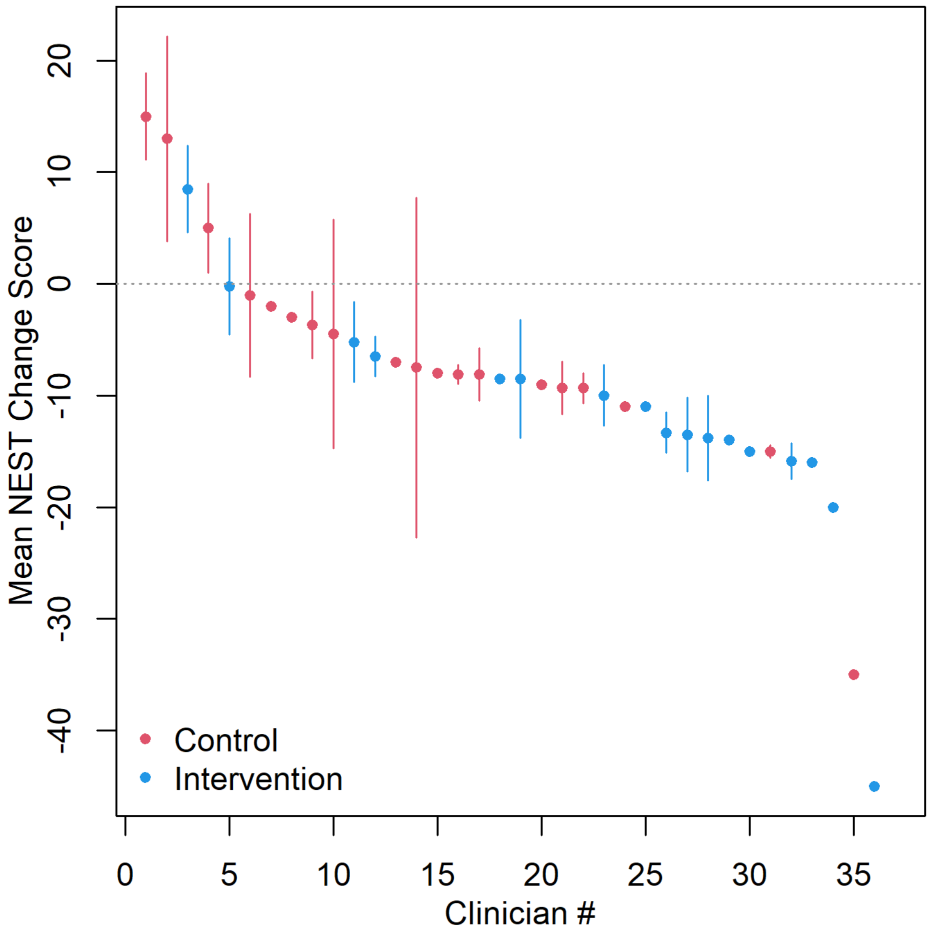

These are plots of mean change scores for the NEST between days 1 and 3 by clinician (intervention = blue, control = red) group, arranged by lowest to greatest total mean change score by clinician. Circles represent mean change scores for all patient-family member dyads enrolled under each clinician.

© 2024 Cox CE et al. *JAMA Network Open.*

eFigure 5. Graph of Mean Baseline NEST by Month of Study

Below are summary statistics and plots of baseline NEST score by calendar time, measured in quarters.

```
##          N Nmiss 0%   25%  50%   75% 100%  Mean    SD
## 2019.2  1     0 26 26.00 26.0 26.00   26 26.00    NA
## 2019.3 12     0 20 27.75 33.0 40.75   57 34.58 10.63
## 2019.4 30     0 15 23.25 32.0 50.00  102 36.87 18.88
## 2020.1 11     0 16 19.50 26.0 39.00   74 32.91 18.24
## 2020.2 12     0 18 28.25 38.5 63.75   83 43.58 21.66
## 2020.3  7     0 13 18.00 35.0 44.00   51 31.86 15.52
## 2020.4 11     0 13 19.50 27.0 47.50  104 38.36 28.60
## 2021.1  3     0 44 44.50 45.0 50.50   56 48.33  6.66
## 2021.2  5     0 15 22.00 31.0 36.00   39 28.60  9.96
## 2021.3 13     0 10 27.00 36.0 40.00   68 34.77 14.35
## 2021.4  4     0 14 31.25 47.0 58.25   62 42.50 21.86
## 2022.1  2     0 34 35.75 37.5 39.25   41 37.50  4.95
```

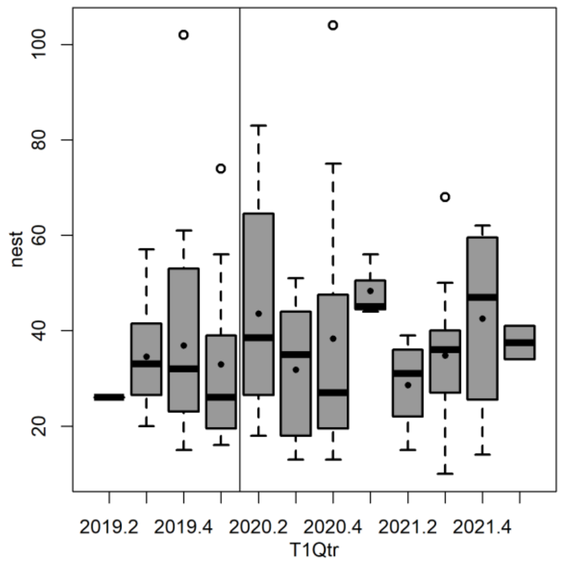

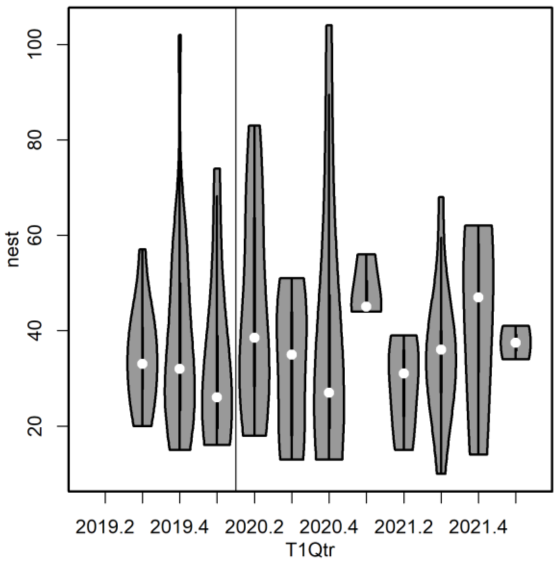

Below is a plot of the mean and median trend in baseline NEST score over time.

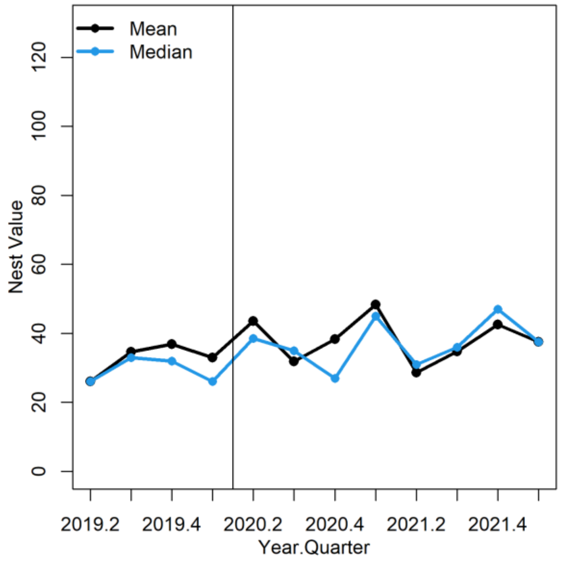

© 2024 Cox CE et al. *JAMA Network Open.*

eFigure 6. Primary Outcomes at Day 3 by Treatment Group, Race, and Physician

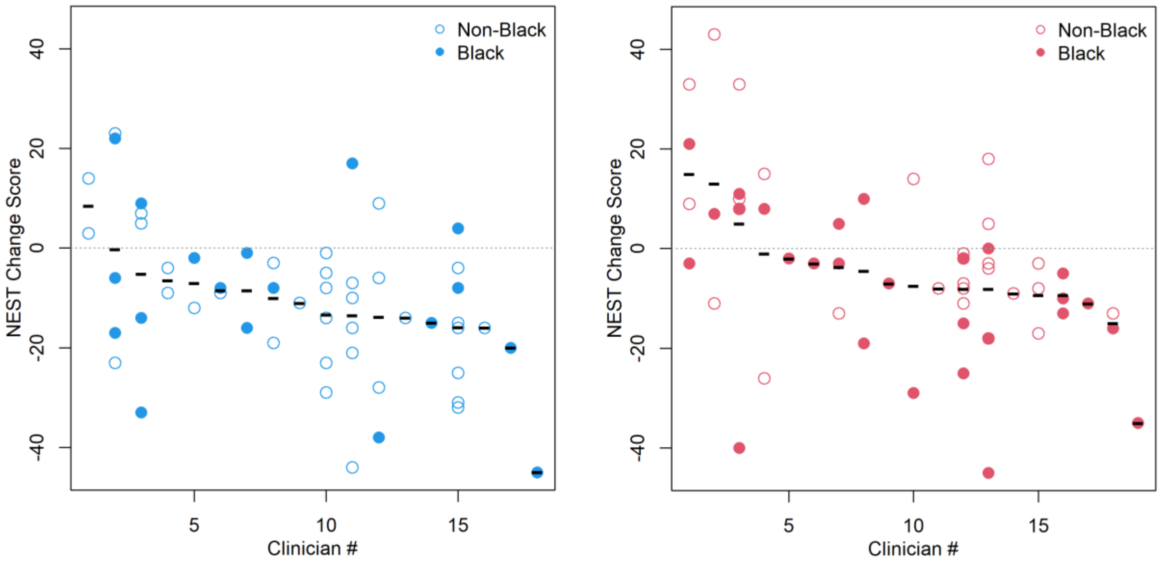

These are plots of mean change scores for the NEST between days 1 and 3 by clinician (intervention = blue, control = red) groups, arranged by lowest to greatest total mean change score for each clinician. Circles represent mean change scores for each patient-family member dyad (filled = Black participant, unfilled = White participant) assigned to each clinician during the trial. 13 clinicians only had 1 patient, 6 clinicians had 2 patients, and 1 clinician had 9 patients.

© 2024 Cox CE et al. *JAMA Network Open.*

eFigure 7. NEST Item Changes Over Time

Below are univariate plots of the change in each NEST item, numbered as Supplement part 7 above:

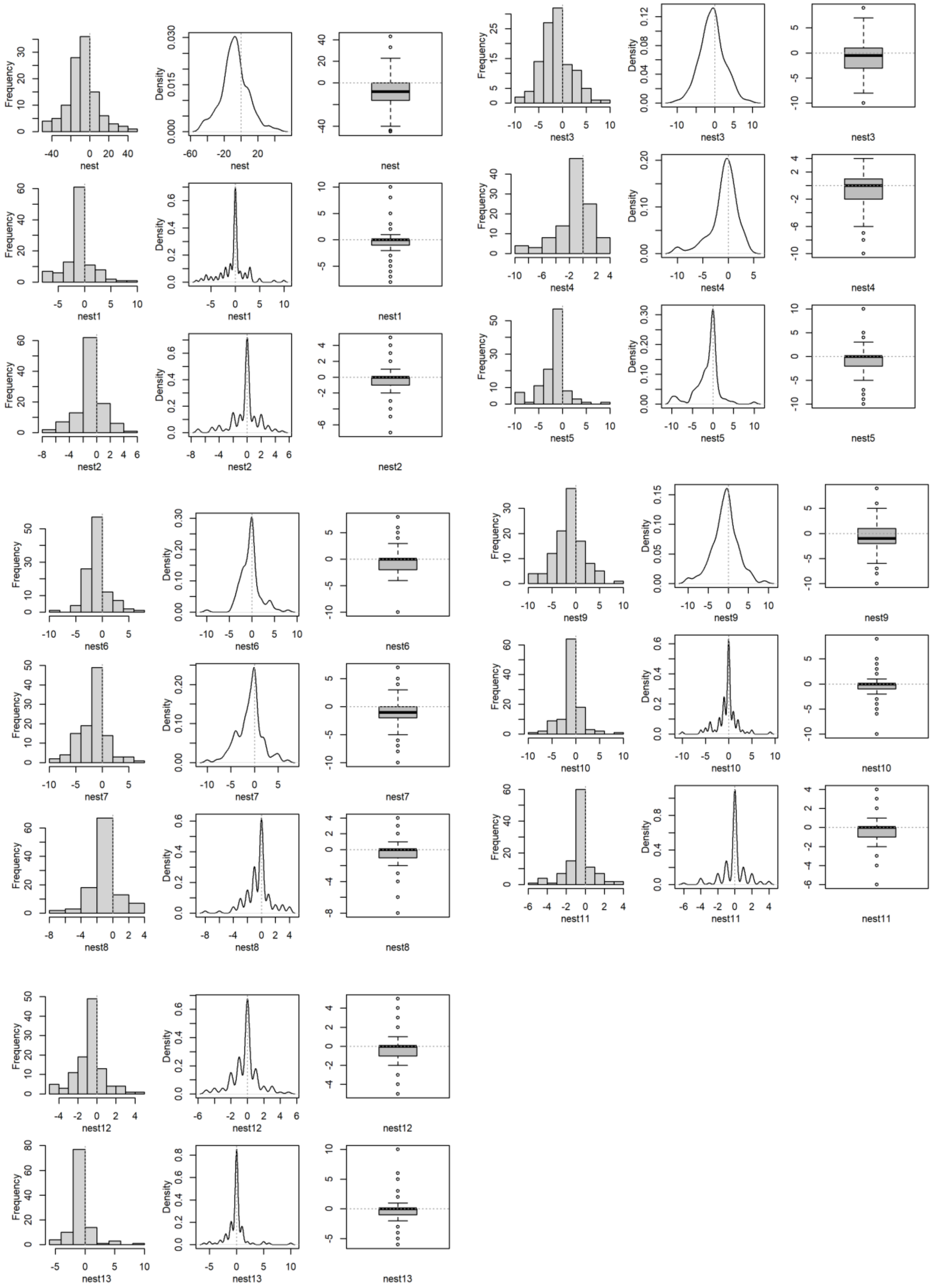

© 2024 Cox CE et al. *JAMA Network Open.*

Below are boxplots and density plots of change in each NEST item, stratified by arm and arm by race.

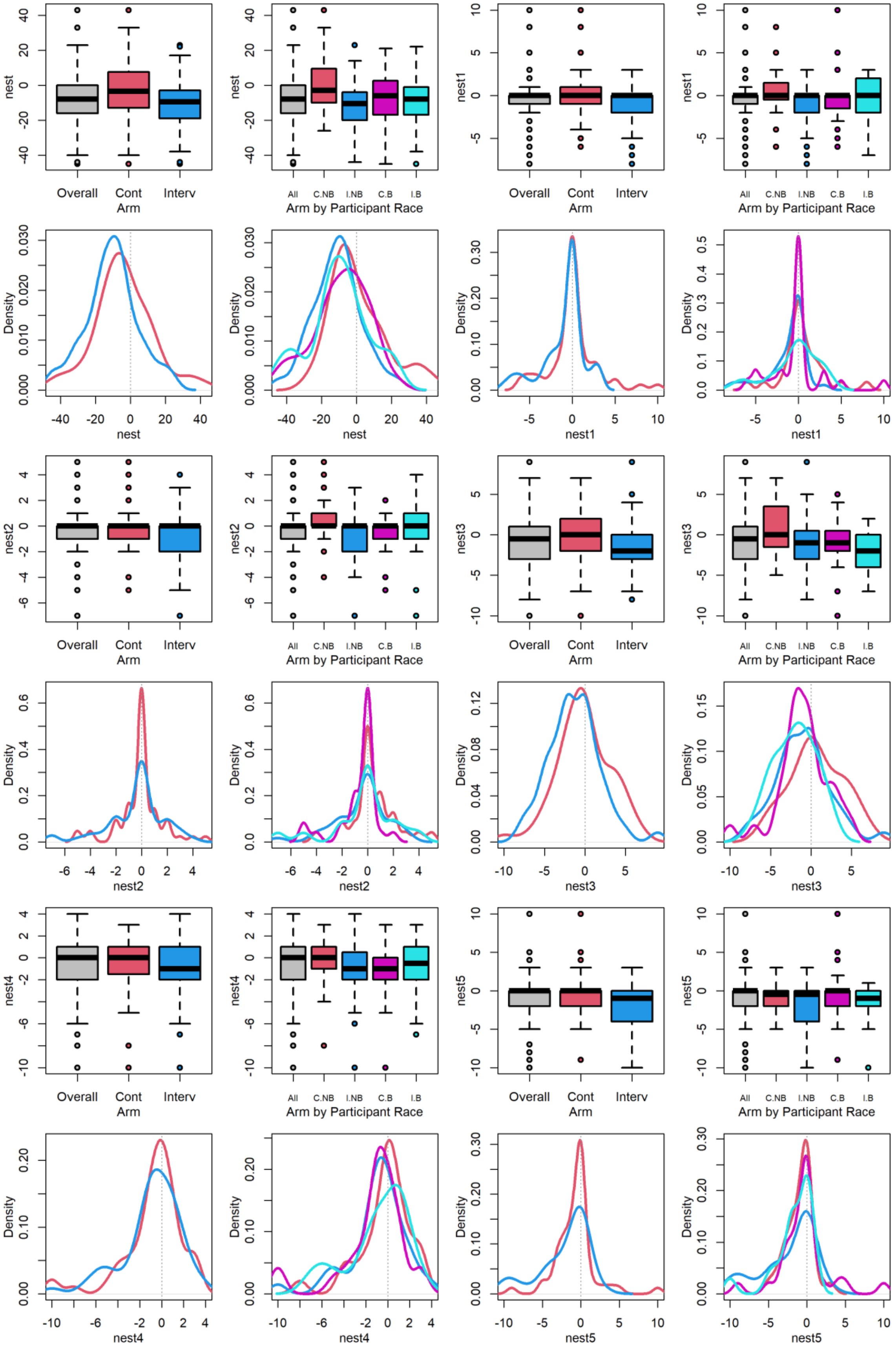

© 2024 Cox CE et al. *JAMA Network Open.*

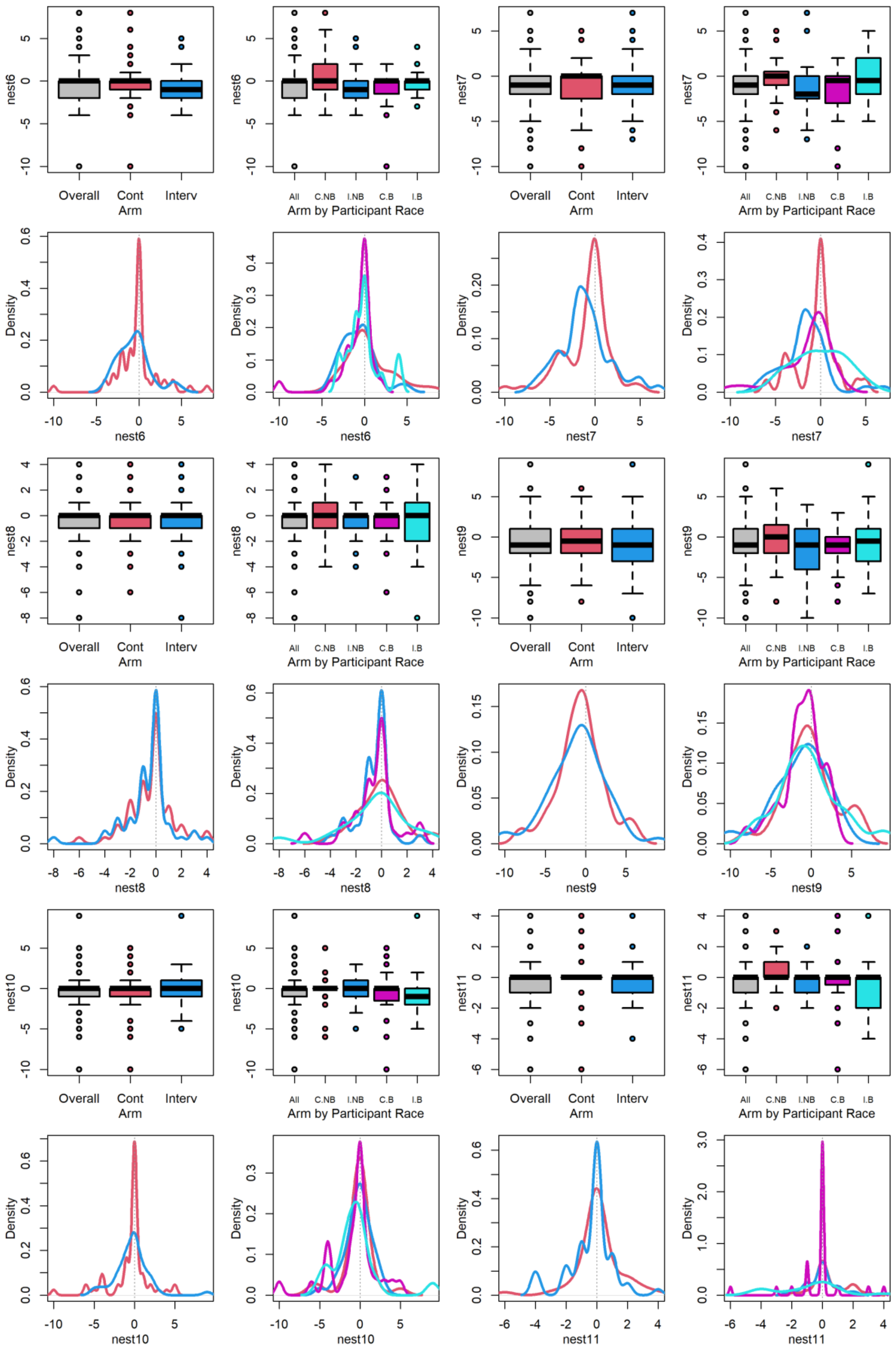

© 2024 Cox CE et al. *JAMA Network Open.*

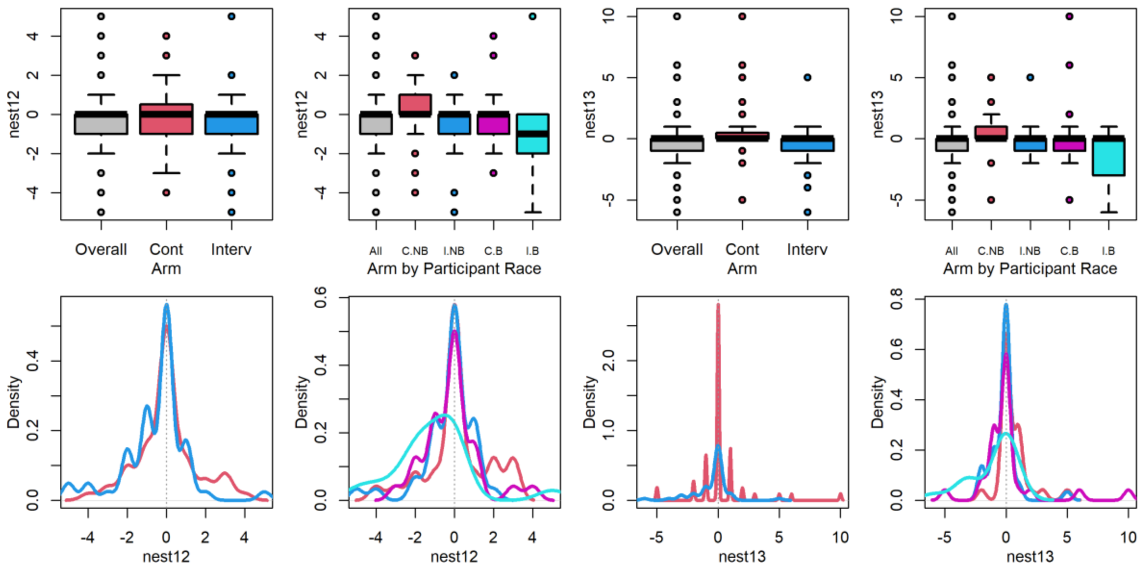

© 2024 Cox CE et al. *JAMA Network Open.*
