# Supplementary material for: Mobile Application–Based Communication Facilitation Platform for Family Members of Critically Ill Patients: A Randomized Clinical Trial
Source: JAMA Netw Open. 2024 Jan 4;7(1):e2349666. doi: 10.1001/jamanetworkopen.2023.49666 (PMC10767607; doi:10.1001/jamanetworkopen.2023.49666)
Supplement: Supplement 3. — Data Sharing Statement [file jamanetwopen-e2349666-s003.pdf]

Data Sharing Statement

Data
Data available: No

Additional Information
Explanation for why data not available: We will follow all NIH guidelines on this issue.
